# Preparation of Oxysterols by C–H Oxidation of Dibromocholestane with Ru(Bpga) Catalyst

**DOI:** 10.3390/molecules27010225

**Published:** 2021-12-30

**Authors:** Yui Fujii, Makoto Yoritate, Kana Makino, Kazunobu Igawa, Daiki Takeda, Daiki Doiuchi, Katsuhiko Tomooka, Tatsuya Uchida, Go Hirai

**Affiliations:** 1 Graduate School of Pharmaceutical Sciences, Kyushu University, 3-1-1 Maidashi, Higashi-ku, Fukuoka 812-8582, Fukuoka, Japan; ys2k_isu_tehe0120@icloud.com (Y.F.); m.yoritate@phar.kyushu-u.ac.jp (M.Y.); makino.kana.230@s.kyushu-u.ac.jp (K.M.); takeda.daiki.116@s.kyushu-u.ac.jp (D.T.); 2 Institute for Materials Chemistry and Engineering, Integrated Research Consortium on Chemical Sciences (IRCCS), Kyushu University, Kasuga 816-8580, Fukuoka, Japan; kigawa@cm.kyushu-u.ac.jp (K.I.); ktomooka@cm.kyushu-u.ac.jp (K.T.); 3 Department of Chemistry, Graduate School of Science, Kyushu University, 744 Motooka, Nishi-ku, Fukuoka 819-0395, Fukuoka, Japan; doiuchi.daiki.180@s.kyushu-u.ac.jp (D.D.); uchida.tatsuya.045@m.kyushu-u.ac.jp (T.U.)

**Keywords:** cholesterol, oxysterol, C–H oxidation, Ru (bpga), 25-hydroxycholesterol, dihydroxycholesterol

## Abstract

Seven mono- and dihydroxycholesterols were prepared by direct C–H oxidation of the cholestane skeleton with a recently developed Ru(Bpga) catalyst (Ru(Bpga) = [RuCl (bpga) (PPh_3_)] Cl; bpga = 2-(bis(pyridin-2-ylmethyl)amino)-*N*-(2,6-dimethylphenyl)acetamide)). Due to the high selectivity of the Ru(Bpga) complex for tertiary C–H, the reaction afforded a mixture of 25-, 20-, 17-, and 14-oxygenated cholesterols that could be easily separated by high-performance liquid chromatography. These results suggest that late-stage C–H oxidation could be a viable strategy for preparing candidate metabolites of biologically important molecules.

## 1. Introduction

Oxysterols are characterized by the presence of one or more hydroxyl groups or a keto group in the ring and/or in the side chain of cholesterol and have multiple functions in cells [1] including regulation of the biosynthesis of steroidal hormones such as 25-hydroxycholesterol, 25-HC, and 27-hydroxycholesterol, 27-HC) [2,3] as well as the biosynthesis of cholesterol (25-HC or others) [4]. They also serve as bile acid precursors (7α-hydroxycholesterol, 7α-HC and 27-HC) [5,6] and as ligands of nuclear receptors such as estrogen receptor (ER) and liver X receptor (LXR) [7,8]. Although oxysterols are associated with the development of several diseases, analysis of oxysterols is still difficult due to the presence of multiple molecular species in biological fluids and organs because oxysterols are ingested from the diet as well as being biosynthesized by cholesterol oxidation in vivo. In these circumstances, lipidomic analysis is important for clarifying the relationship between oxysterols and disease [9].

Organic synthesis of cholesterol derivatives is important for lipidomic analysis of oxysterols in order to provide authentic standard materials. The majority of the target oxysterols are 7- or 12-hydroxycholesterol derivatives, which are intermediates in bile acid synthesis. Analysis of oxysterols such as 20α-hydroxycholesterol, 24*S*-hydroxycholesterol, 24*S*,25-epoxycholesterol, 25-HC, 27-HC, and so on, has recently been reviewed [9]. These oxysterols are well known as metabolites; for example, 24*S*-HC is endogenously synthesized by cholesterol 24-hydroxylase (CYP46A1) in the brain and contributes to cholesterol homeostasis [10]. However, the lack of standard materials has hampered the analysis of other minor oxysterols, which might also have significant biological functions.

**Figure 1:**
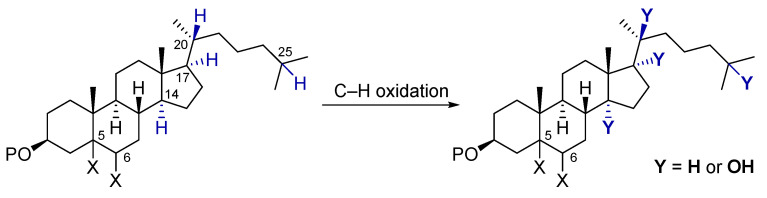
C–H oxidation strategy for the preparation of hydroxycholesterols.

There have already been many reports on the chemical synthesis of oxysterols from commercially available (but sometimes expensive) steroidal molecules. From the viewpoint of the preparation of a wide variety of oxysterols, however, catalyst-driven C–H oxidation of cholesterol or its derivatives is an attractive strategy [11]. For example, Barton and co-workers showed in 1985 that direct oxidation of 3β,5α,6β-triacetoxycholestane by use of the Gif system (iron cluster-metallic zinc-pyridine-acetic acid-oxygen) provided a variety of oxidized cholestanes, most of which were ketone derivatives generated by oxidation of the methylene group in the cholestane skeleton [12]. On the other hand, two of the present authors (Doiuchi and Uchida) recently developed an Ru (bpga)-catalyzed C–H oxidation reaction with high tertiary C–H bond selectivity and an excellent catalyst turnover number (TON = 26,000 in oxidation of adamantane) [13,14]. We envisioned that Ru (bpga)-catalyzed C–H oxidation of protected cholesterol would afford a variety of hydroxycholesterols via the oxidation of tertiary C–H (C25, C20, C17, and C14); these are not formed by oxidation using the Gif system (Figure 1). Here, we report the synthesis and separation/purification of a series of oxysterols obtained in this way, together with their NMR spectra (500 MHz) and the results of crystallographic analysis.

## 2. Results

### 2.1. C–H Oxidation of ***2-Ac*** with Ru(Bpga)

**Scheme 1:**
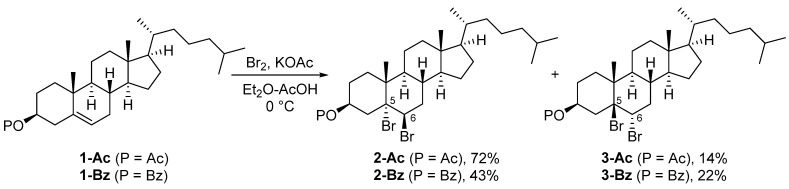
Preparation of substrates **2-Ac** and **2-Bz**.

In order to avoid oxidation of the allylic position of cholesterol, which would yield side-chain or D-ring oxidation products, we chose 5,6-dibromocholestane as a substrate for C–H oxidation. Bromination of cholesterol acetate **1-Ac** or the benzoate **1-Bz** provided 5α,6β-dibromocholestane **2-Ac** or **2-Bz** as the major isomer (Scheme 1) [15]. Separation of the corresponding 5β,6α-isomer **3-Ac** or **3-Bz** readily afforded pure **2-Ac** or **2-Bz**.

Next, we examined C–H oxidation of the acetate **2-Ac** with Ru(Bpga) (PPh_3_) Cl_2_ complex, and obtained a mixture of oxidized products. The formation of isomers through dyotropic rearrangement reaction [16,17] further complicated the analysis. Nevertheless, the 25-HC derivative **4-Ac** and its dyotropic isomer **5-Ac** were clearly separated by thin layer chromatography (TLC) from other isomers and the major products in this C–H oxidation. Thus, we optimized the reaction conditions based on the formation of **4-Ac** and **5-Ac** and low recovery of the starting material (calculated by combining **2-Ac** and **3-Ac**, because **2-Ac** was also isomerized to **3-Ac** under the reaction conditions).

First, the reaction was conducted with 2 moL% of the Ru catalyst, iodosobenzene (PhIO) as an oxidant, and trifluoroacetic acid (TFA) as an additive (Table 1, entries 1–4) [13]. Increasing the amount of TFA decreased the conversion rate (entries 1–4), and 0.1–0.2 equivalent of TFA gave a 26–28% yield of 25-OHC derivatives **4-Ac** and **5-Ac**, along with 19–22% recovery of the starting material. Increasing the concentration from 0.2 equivalent to 0.4 equivalent resulted in increased recovery of the starting materials, probably due to the low solubility of PhIO (entry 5). Use of *m*CPBA as the oxidant was not effective for this substrate (entry 6). In contrast, we found that reaction with iodobenzene diacetate (PIDA) in the presence of 2.8 equivalent of water gave 25-HC derivatives **4-Ac** and **5-Ac** in 34% yield (46% based on the recovered starting materials) together with **2-Ac** and **3-Ac** in 26% yield. We chose this condition for further studies. It should be noted that this is one of the best synthetic procedures so far reported for the direct formation of 25-HC from cholestanes or cholesterols [15,18,19,20,21]. As little as 2 moL% Ru(Bpga) catalyst gave the 25-HC derivatives in 46% yield, indicating the robustness of the catalyst under oxidative conditions.

### 2.2. Oxidation of ***2-Bz*** with Ru(Bpga) and Analysis of Products

For the structural identification of minor isomers, we decided to use **2-Bz** as a substrate and we treated the products with Zn/AcOH after C–H oxidation reaction to transform all dibromo derivatives to 5, 6-olefin derivatives. Use of the Bz group instead of Ac group facilitates detection of the products during separation by chromatography (or HPLC).

**Figure 2:**
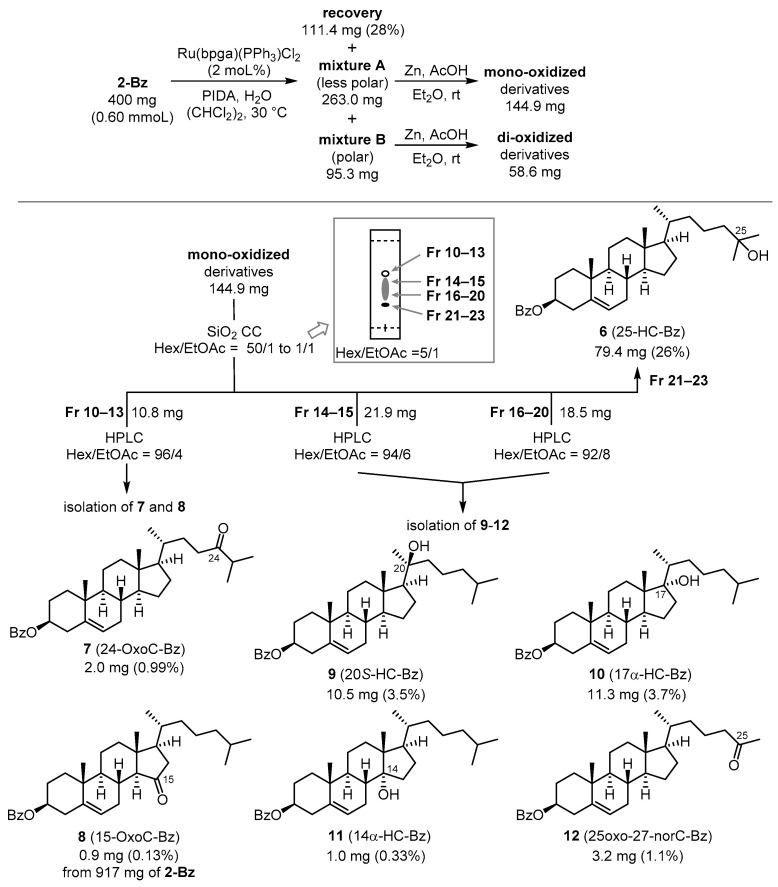
C–H oxidation of **2-Bz** with Ru(Bpga) and identification of mono-oxidized products.

Similar to the reaction of **2-Ac**, C–H oxidation of **2-Bz** produced several oxidized products (Figure 2), and 25-HC derivatives were also observed in TLC. We roughly separated the resulting mixture into three parts, starting materials **2-Bz** and **3-Bz** (28%), 25-HC derivatives and less polar materials (**mixture A**), and more polar materials (**mixture B**). **Mixture A** and **mixture B** were each treated with Zn/AcOH to give the corresponding mixture of compounds with a 5,6-olefin functionality. Mass spectrometric analysis indicated that the products in **mixture A** were mainly mono-oxidized products (hydroxy and keto derivatives), and those in **mixture B** were di-oxidized derivatives.

**Figure 3:**
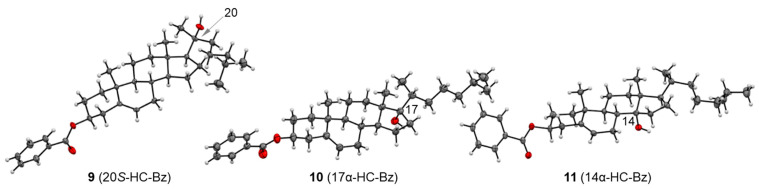
Crystal structures of **9** (CCDC Number 2123039), **10** (CCDC Number 2123035), and **11** (CCDC Number 2123036).

**Figure 4:**
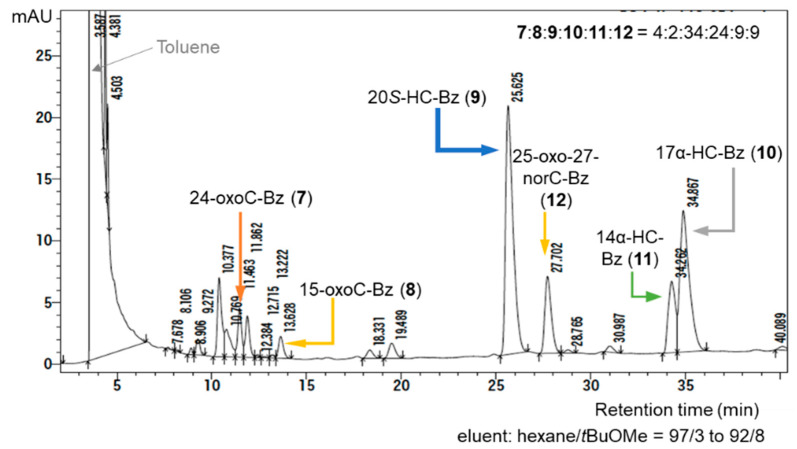
HPLC analysis of mono-oxidized derivatives (PDA: 254 nm detection).

The mono-oxidized derivatives were roughly divided into four parts by SiO_2_ column chromatography (Figure 2). The most polar fraction on TLC (Fr 21–23) afforded Bz-protected 25-HC (**6**, 25-HC-Bz) as a single product (26%, 36% brsm). HPLC analysis of Fr 10–13 showed multiple peaks (see Supplementary Materials) due to a mixture of products, among which we identified the 24-oxo derivative (**7**, 24-oxoC-Bz). Fr 14–15 contained four compounds, which were separated by HPLC (see Supplementary Materials) and characterized as 20*S*-HC-Bz (**9**), 17α-HC-Bz (**10**), 14α-HC-Bz (**11**), and a small amount of 25-oxo-27-norC-Bz (**12**). The stereochemistry of **9**–**11** was confirmed by X-ray crystallographic analysis, showing that the C–H oxidation reaction was stereo-retentive (Figure 3). Compounds **9**, **10**, and **12** were also isolated from Fr 16–20, indicating high tertiary C–H selectivity of the Ru(Bpga) catalyst. HPLC analysis of the mono-oxidized derivatives showed a product ratio of **7**:**8**:**9**:**10**:**11**:**12** = 4:2:34:24:9:9 (Figure 4).

**Figure 5:**
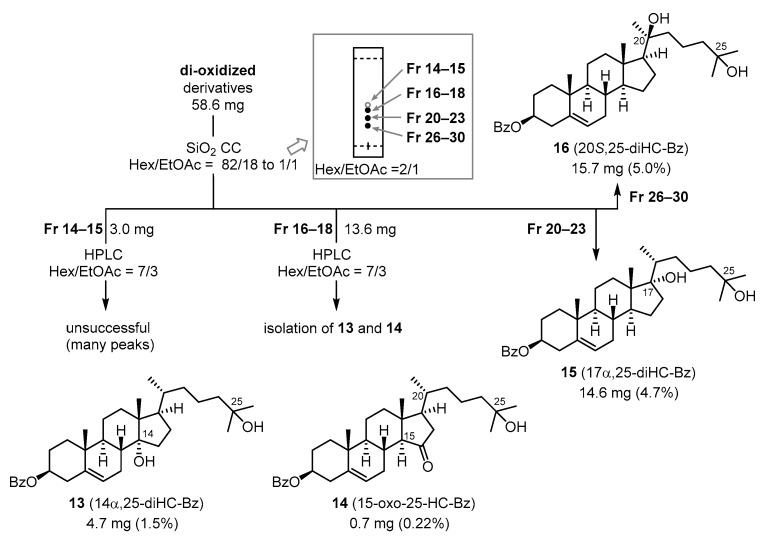
Identification of di-oxidized products.

**Figure 6:**
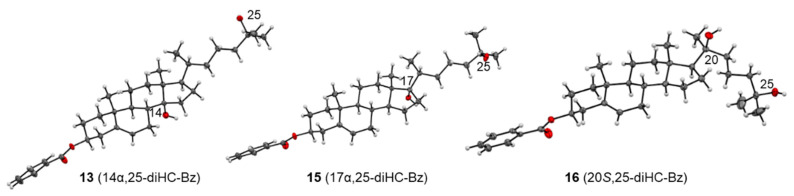
Crystal Structures of **13** (CCDC Number 2123037), **15** (CCDC Number 2123038), and **16** (CCDC Number 2123040).

**Figure 7:**
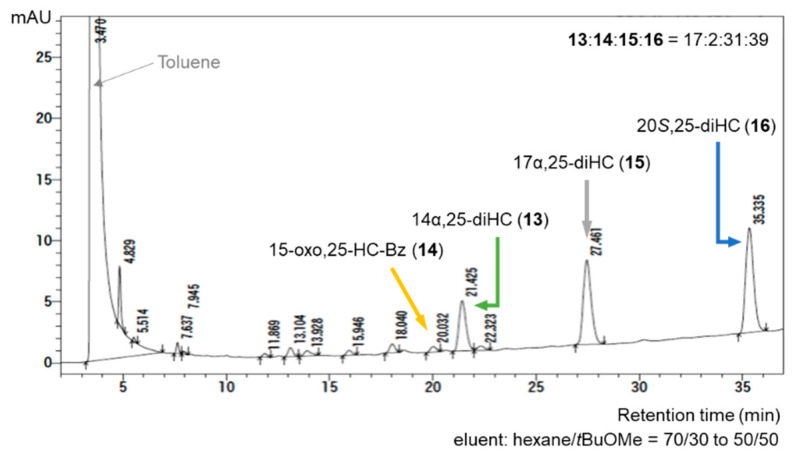
HPLC analysis of di-oxidized derivatives (PDA: 254 nm detection).

Similar separation procedures were applied to the di-oxidized derivatives (Figure 5). Although no material was isolated from Fr 14–15, Fr 16–18 contained 14α,25-diHC-Bz (**13**) and a trace amount of 15-oxo-25-HC-Bz (**14**), together with unidentified compounds. Fr 20–23 and Fr 26–30 contained almost pure 17α,25-diHC-Bz (**15**) and 20*S*,25-diHC-Bz (**16**), respectively. The stereochemistry of **13**, **15**, and **16** was confirmed by X-ray crystallographic analysis (Figure 6). The results suggested that a second C–H oxidation of the major product 25-HC-Bz (**6**) occurred in a highly tertiary C–H selective and stereo-retentive manner. Random oxidation is presumably suppressed to some extent by the high selectivity of the Ru(Bpga) catalyst, and this enabled the isolation of 10 D-ring or side-chain-oxidized cholesterol derivatives at once from only 400 mg of 5α,6β-dibromocholestane **2-Bz**. HPLC analysis of the di-oxidized derivatives confirmed successful isolation of the major products in a ratio of **13**:**14**:**15**:**16** = 17:2:31:39 (Figure 7).

### 2.3. Oxidation of ***2-Bz*** with Fe (S,S-PDP) and Analysis of Products

**Figure 8:**
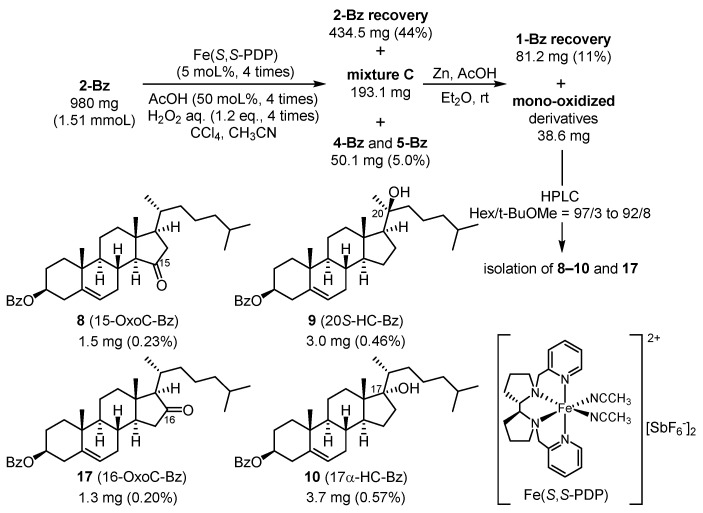
C–H oxidation of **2-Bz** with Fe (*S*,*S*-PDP) and identification of mono-oxidized products.

**Figure 9:**
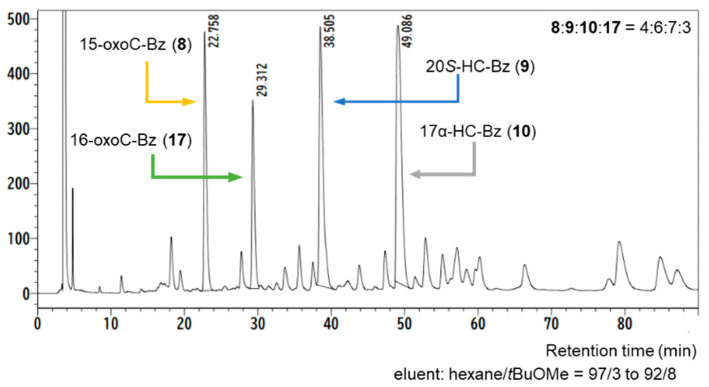
HPLC analysis of mono-oxidized derivatives (PDA: 254 nm detection).

We also examined the C–H oxidation of **2-Bz** with Fe (S,S-PDP) (White’s catalyst; Fe (*S*,*S*-PDP) = [Fe (*S*,*S*-PDP) (MeCN)_2_][SbF_6_]_2_; *S*,*S*-PDP = (2*S*,2′*S*)-1,1′-bis (pyridin-2-ylmethyl)-2,2′-bipyrrolidine) [22], which is one of the best tertiary-selective C–H oxidation catalysts available for late-stage functionalization (Figure 8) [23]. Reaction of **2-Bz** under the reported optimum conditions with a slight modification (use of CCl_4_ as a co-solvent because of the low solubility of **2-Bz** in CH_3_CN) resulted in low conversion of C–H oxidation. We added the Fe (*S*,*S*-PDP) catalyst, H_2_O_2_, and acetic acid four times in order to consume as much starting material as possible, but almost half of the **2-Bz** was recovered (44%). 25-HC-Bz derivative **4-Bz** (see Table 1) was formed as a major product (total 5.0%), as seen with the Ru(Bpga) catalyst. After reduction with Zn/AcOH, identification of minor isomers revealed, in addition to 15-oxoC-Bz (**8**, 0.23%), 20S-HC-Bz (**9**, 0.46%), and 17α-HC-Bz (**10**, 0.57%), that 16-oxoC-Bz (**17**) was formed in 0.20% yield (calculated yields). Recovered **1-Bz** (11%) might be derived from **3-Bz** generated in the C–H oxidation reaction and/or incomplete separation of **2-Bz** prior to Zn-mediated reduction. Many minor and major products of this reaction could be isolated by HPLC, and **8**:**9**:**10**:**17** were obtained in a ratio of 4:6:7:3 (Figure 9). These results indicate that changing the catalyst has the potential to provide different oxidized cholesterols in a different ratio, although oxidation of cholesterol derivatives by Fe (*S*,*S*-PDP) was not efficient under the reported conditions.

### 2.4. Preparation of Hydroxycholesterols and Dihydroxycholesterols

**Scheme 2:**
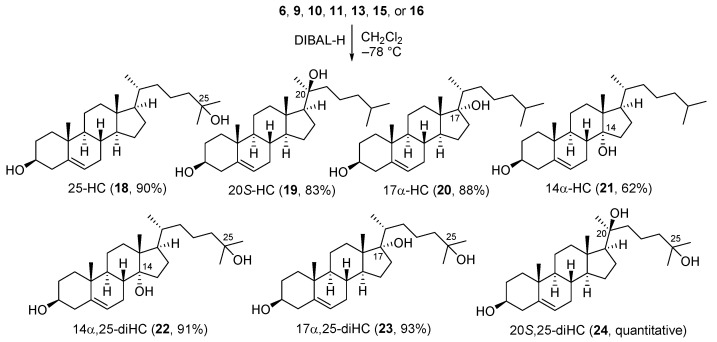
Preparation of **18**–**24**.

Removal of the benzoyl groups of **6**, **9**, **10**, **11**, **13**, **15**, and **16** was conducted with diisobutylaluminum hydride (DIBAL-H) to give 25-HC (**18**), 20*S*-HC (**19**), 17α-HC (**20**), 17α,25-diHC (**23**), and 20*S*,25-diHC (**24**) in reasonable yields (Scheme 2).

## 3. Discussion

Several hydroxycholesterols and dihydroxycholesterols such as 20*S*-HC (**19**) [24,25,26,27] and 20*S*,25-diHC (**24**) [28,29] have previously been synthesized in a target-oriented manner by means of semi-synthetic approaches from available steroidal molecules. As far as we know, however, no chemical synthesis of 17α-HC (**20**), 14α-HC (**21**), 14α,25-diHC (**22**), and 17α,25-diHC (**23**) has yet been reported, though **20** was shown to be produced from cholesterol by P450l7α (CYP17A1) [30]. These D-ring-modified cholesterols are not easy to access directly by semi-synthesis from abundant steroidal molecules, highlighting the usefulness of the C–H oxidation strategy.

The Ru (bpga)-catalyzed oxysterol synthesis by C–H oxidation offers two advantages. First, the catalyst loading can be suppressed to 2 moL%, although approximately 30% of the starting material was recovered. In addition, high tertiary C–H selectivity suppressed the formation of oxo-cholesterol derivatives. These features enabled facile separation of the obtained mixtures as well as selective formation of the targeted hydroxycholesterols. We did not investigate in detail the isolation of dihydroxycholesterols in the case of the Fe (*S*,*S*-PDP) catalytic system with a high catalyst loading, because of contamination with decomposition products probably derived from the Fe-complex. However, Fe-catalyzed C–H oxidation afforded 16-oxo cholesterol derivatives that was not formed in the Ru-catalyzed reaction, suggesting that other C–H oxidation catalysts might also produce different sets of oxysterols.

## 4. Materials and Methods

### Oxidation of ***2-Bz***

To a 10 mL screw-cap vial was added **2-Bz** (400 mg, 615 µmol, 1.0 equiv.), iodobenzene diacetate (PIDA, 198 mg, 615 µmol, 1.0 equiv.), H_2_O (30 μL), (CHCl_2_)_2_ (1.5 mL), and a magnetic stir bar. The vial was placed on an aluminum block at 30 °C, added [RuCl(bpga)(PPh_3_)]Cl (9.6 mg, 12 μmol, 2 mol%), and sealed with a screw cap. After stirring for 24 h, the suspended brown mixture was turned to be clear brown solution. Then PIDA (198 mg, 1.0 equiv.) was added to the mixture. After stirring for 41 h, the mixture was filtered through a pad of Celite^®^ and concentrated. The residue was purified by silica gel column chromatography (eluent: hexane/EtOAc 50/1 to 1/2). to give less polar **mixture A** (263 mg), polar **mixture B** (95.3 mg), and a mixture of recovered **2-Bz** and **3-Bz** (111.4 mg, 28%, **2-Bz**:**3-Bz** = 1:3).

## 5. Conclusions

Our C–H oxidation strategy using Ru(Bpga) catalyst afforded a set of four hydroxycholesterols and three dihydroxycholesterols that were readily separable. These oxysterols are expected to be useful as standard materials for lipidomics analysis as well as in bioactivity assay systems [31].

## Figures and Tables

**Table 1:**
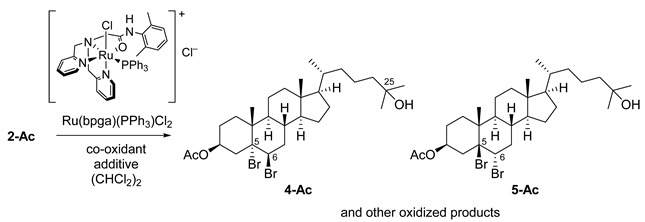
Oxidation of **2-Ac** with the [RuCl (bpga) (PPh_3_)] Cl catalyst and yields of 25-HC derivatives.

Entry	Oxidant	Additive (Equivalent)	Time (h)	Yields		
				4-Ac	5-Ac	Recovery
1 ^1^	PhIO ^2^	TFA (0.1)	70	9	17	19
2 ^1^	PhIO ^2^	TFA (0.2)	43.5	13	15	22
3 ^1^	PhIO ^2^	TFA (0.4)	43.5	10	15	28
4 ^1^	PhIO ^2^	TFA (1.0)	21.5	7	9	47
5 ^3^	PhIO ^2^	TFA (0.1)	94.5	8	14	37
6 ^3^	*m*CPBA	None	92	4	9	58
7 ^3^	PIDA	H_2_O (2.8)	92	10	24	26

^1^ Substrate concentration 0.2 M; ^2^ PhIO (1 equivalent) was added twice (total 2 equivalent); ^3^ Substrate concentration 0.4 M.

## Data Availability

The data presented in this study are available in supplementary material.

## Supplementary Materials

The following supporting information can be downloaded online. Experimental procedure; Figure S1: Purification of oxidized cholesterols (part 1); Figure S2: Purification of oxidized cholesterols (part 2); Figure S3: Characteristic HMBC correlation for structural determination of **7**; Figure S4: Characteristic HMBC and COSY correlations for structural determination of **8**; Figure S5: Characteristic HMBC correlation for structural determination of **9**; Figure S6: Characteristic HMBC correlations for structural determination of **10**; Figure S7: Characteristic HMBC correlations for structural determination of **11**; Figure S8: Characteristic HMBC correlations for structural determination of **12**; Figure S9: Purification of oxidized cholesterols (part 3); Figure S10: Characteristic HMBC correlations for structural determination **13**; Figure S11: Characteristic HMBC and COSY correlations for structural determination of **14**; Figure S12: Characteristic HMBC correlations for structural determination of **15**; Figure S13: Characteristic HMBC correlations for structural determination of **16**; Figure S14: Characteristic HMBC and COSY correlations for structural determination of **17**; NMR spectra.

## Sample Availability

Some of the compounds described in this article are available from the authors.
