# Supplementary material for: Preparation of Oxysterols by C–H Oxidation of Dibromocholestane with Ru(Bpga) Catalyst
Source: Molecules. 2021 Dec 30;27(1):225. doi: 10.3390/molecules27010225 (PMC8746986; doi:10.3390/molecules27010225)
Supplement: Supplementary file 1 [file molecules-27-00225-s001.zip › molecules-1524546-supplementary.pdf]

Supporting Information

Preparation of Oxysterols by C-H Oxidation of Dibromocholestane with Ru(bpga) Catalyst

Yui Fujii [1], Makoto Yoritate [1], Kana Makino [1], Kazunobu Igawa [2], Daiki Takeda [1], Daiki Doiuchi [3], Katsuhiko Tomooka [2], Tatsuya Uchida [3], and Go Hirai [1,*]

[1] *Graduate School of Pharmaceutical Sciences, Kyushu University, 3-1-1 Maidashi, Higashi-ku, Fukuoka 812-8582, Japan; gohirai@phar.kyushu-u.ac.jp*

[2] *Institute for Materials Chemistry and Engineering, IRCCS, Kyushu University, Kasuga, Fukuoka 816-8580, Japan*

[3] *Department of Chemistry, Graduate School of Science, Kyushu University, 744 Motooka, Nishi-ku, Fukuoka 819-0395, Japan*

* *Correspondence: gohirai@phar.kyushu-u.ac.jp*

Table of Contents

1. General Remarks **S2**

2. Experimental Procedure

2-1: Preparation and oxidation of 3-Ac-cholesterols **S3~S5**

2-2: Preparation and oxidation of 3-Bz-cholesterols **S6~S17**

2-3: Deprotection of 3-Bz-cholesterols **S18~S20**

3. NMR Spectra **S21~S51**

1. General Remarks

Experiment and Reagents;

Chemistry: In general, reactions were carried out under a nitrogen atmosphere, unless noted otherwise. Reagent grade solvents (pyridine) was distilled prior to use. Reactions were monitored by thin layer chromatography using Merck Silica Gel 60 F254 plates. Flash chromatography was performed using flash silica gel 60N (spherical neutral, particle size 40−50 μm) purchased from Kanto Chemical Co. Ltd., unless noted otherwise. Zn powder was purchased from TCI, cat. No. Z0015. White's catalyst [Fe(*S*,S-PDP)$(MeCN)_2$][SbF_6]$_2$; *S*,*S*-PDP = (2*S*,2'*S*)-1,1'-bis(pyridin-2-ylmethyl)-2,2'-bipyrrolidine) was prepared according to the reported method; White, M. C. *et al.*, *Science* **2007**, *318*, 783-787. [RuCl(bpga)(PPh_3)]Cl complex (bpga = 2-(bis(pyridin-2-ylmethyl)amino)-*N*-(2,6-dimethylphenyl)acetamide) was prepared according to our previously reported method; Uchida, T. *et al.*, *Chem. Asian J.* **2020**, *15*, 762-765.

Instrumentation;

Chemistry: NMR spectra were recorded 500 MHz Bruker Advance III, operating at 500 MHz for ^{1}H NMR, and 126 MHz for ^{13}C NMR. Chemical shifts were reported in the scale relative to $CHCl_3$ (δ 7.26 ppm for ^{1}H NMR, δ 77.16 ppm for ^{13}C NMR) as an internal reference. Splitting patterns are designated as s: singlet, d: doublet, t: triplet, br: broad, and m: multiplet. High-resolution electron splay ionization ESI-MS was obtained with Bruker MicrOTOF II. HPLC analysis was performed on HITACHI HPLC system consisting of the followings: pump, L6250; detector, L-3350 RI monitor; column, Senshu-Pak PEGASIL silica SP100; mobile phase, hexane/ethyl acetate, and JASCO HPLC system consisting of the followings: pump, PU-2087; detector, UV-2070 UVdetector; column, Senshu-Pak PEGASIL silica SP100; mobile phase, hexane/EtOAc.

2. Experimental Procedure

2-1: Preparation and oxidation of 3-Ac-cholesterol

Compound **1-Ac**

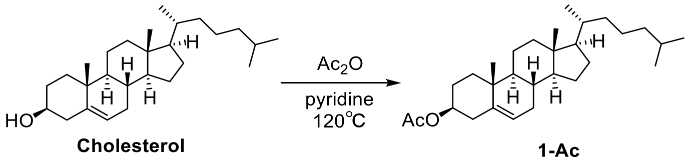

To a solution of cholesterol (3.01 g, 7.76 mmol, 1.0 equiv.) in pyridine (45 mL, 0.17 M) was added Ac_2O (33 mL, 351 mmol, 45 equiv.) at room temperature. The mixture was stirred for 2 h at 120 ºC, turning the color of solution from colorless to brown. The mixture was quenched with $NaHCO_3$ at 0 ºC and extracted with hexane (3 x 30 mL). The combined organic layers were washed with aqueous saturated $NaHCO_3$ and the separated water layer was extracted with hexane. The combined organic layers were dried over Na_2SO_4, filtered, and concentrated. The residue was purified by silica gel column chromatography (eluent: hexane/EtOAc 50/1 to 9/1) to give **1-Ac** (3.11 g, 7.26 mmol, 94%) as a white solid.

^{1}H NMR (500 MHz, CDCl$_3$): δ 5.37 (d, J = 4.9 Hz, 1H), 4.63–4.57 (m, 1H), 2.32 (d, J = 7.0 Hz, 2H), 2.03 (s, 3H), 2.01–1.94 (m, 2H), 1.88–1.79 (m, 3H), 1.62–1.54 (m, 8H), 1.53–1.43 (m, 3H), 1.42–1.04 (m, 8H), 1.02 (s, 3H), 1.01–0.94 (m, 3H), 0.91 (d, J = 6.4 Hz, 3H), 0.86 (dd, J = 6.7, 2.1 Hz, 6H), 0.68 (s, 3H)

Compound **2-Ac** and **3-Ac**

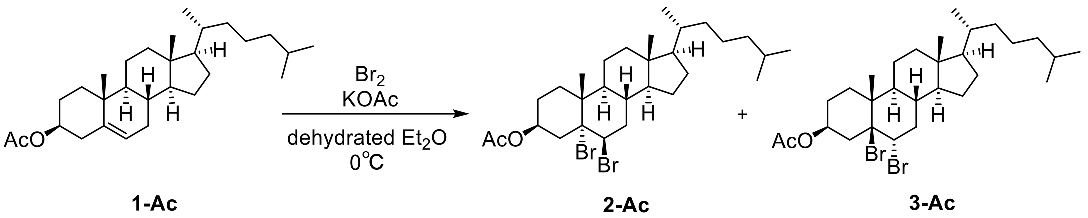

To a solution of **1-Ac** (3.05 g, 7.11 mmol. 1.0 equiv.) in anhydrous Et_2O (86 mL, 82.5 mM) was added a solution of KOAc (5.40 g, 55 mmol, 7.7 equiv.) in glacial acetic acid (54 mL) at 0°C, and then added a solution of Br_2 (3.64 mL, 71.1 mmol, 10 equiv.) in glacial acetic acid (27 mL) at 0°C. After stirring for 3 h at 0°C, the reaction was allowed to worm to room temperature and stirred overnight. The resulting mixture was quenched with aqueous 1 M $Na_2S_2O_3$ at room temperature and extracted with diethyl ether (3 x 30 mL). The combined organic layer was washed with aqueous 1 M $Na_2S_2O_3$ (30 mL), followed by aqueous 1 M K_2CO_3 (150 mL), dried over Na_2SO_4, filtered, and concentrated. The residue was purified by MPLC (Yamazen, Ultrapack 40D eluent: hexane/EtOAc 100/0 to 84/16) to give dibromide **2-Ac** (3.00 g, 5.1 mmol, 72%) and the isomer **3-Ac** (573 mg, 1.0 mmol, 14%).

Compound **2-Ac**:

^{1}H NMR (500 MHz, CDCl$_3$): δ 5.54–5.47 (m, 1H), 4.85 (q, J = 2.1 Hz, 1H), 2.72–2.66 (m, 1H), 2.61 (dd, J = 14.0, 10.4 Hz, 1H), 2.29 (ddd, J = 14.0, 5.5, 1.5 Hz, 1H), 2.07 (s, 3H), 2.10–1.83 (m, 5H), 1.79–1.59 (m, 5H), 1.57–1.52 (m, 1H), 1.49 (s, 3H), 1.47–1.08 (m, 13H), 1.02 (q, J = 9.1 Hz, 1H), 0.93 (d, J = 6.7 Hz, 3H), 0.91–0.88 (dd, J = 6.4, 2.4 Hz, 6H), 0.73 (s, 3H).

^{13}C NMR (126 MHz, CDCl$_3$): δ 170.5, 88.3, 72.2, 56.3, 56.3, 55.3, 47.4, 42.8, 42.1, 42.0, 39.7, 39.7, 37.4, 36.6, 36.3, 35.9, 31.0, 28.3, 28.2, 26.3, 24.2, 24.0, 23.0, 22.7, 21.5, 21.4, 20.3, 18.8, 12.4.

Compound **3-Ac**:

^{1}H NMR (500 MHz, CDCl$_3$): δ 5.14 (s, 1H), 4.86 (dd, *J* = 12.7, 5.0 Hz, 1H), 2.86 (d, *J* = 16.8 Hz, 1H), 2.37 (dd, *J* = 16.8, 4.3 Hz, 1H), 2.25–2.20 (m, 1H), 2.06 (s, 3H), 2.12–2.04 (m, 1H), 2.00 (d, *J* = 12.8 Hz, 1H), 1.87–1.80 (m, 1H), 1.71–1.46 (m, 7H), 1.41–1.25 (m, 7H), 1.23 (s, 3H), 1.17–0.94 (m, 8H), 0.89–0.88 (m, 3H), 0.85 (dd, *J* = 6.7, 2.4 Hz, 6H), 0.64 (s, 3H).

^{13}C NMR (126 MHz, CDCl$_3$): δ 170.6, 79.1, 67.8, 64.2, 56.0 (2C), 44.9, 42.6, 41.7, 41.6, 39.7, 39.5, 36.4, 36.2, 35.7, 34.4, 29.2, 28.2, 28.1, 24.2, 24.1, 23.9, 22.9, 22.6, 22.5, 21.8, 21.3, 18.7, 12.0.

[Oxidation of compound 2-Ac using Ru catalyst]

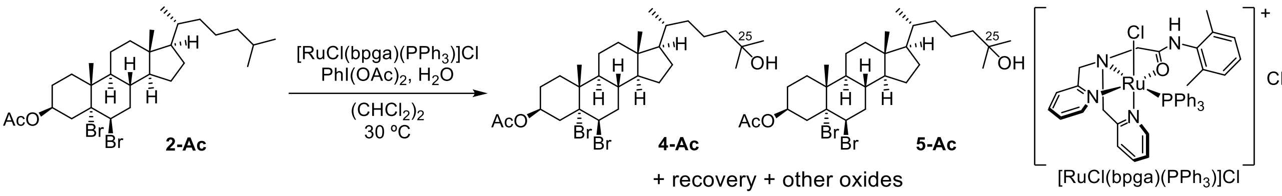

To a 10 mL screw-cap vial was added **2-Ac** (100 mg, 170 μmol, 1.0 equiv.), iodobenzene diacetate (PIDA, 55 mg, 170 μmol, 1.0 equiv.), H_2O (8.5 μL), $(CHCl_2)_2$ (430 μL), and a magnetic stir bar. The vial was placed on an aluminum block at 30 °C, added [RuCl(bpga)(PPh$_3$)]Cl (2.7 mg, 3.4 μmol, 2 mol%), and sealed with a screw cap. After stirring for 24 h, the suspended brown mixture was turned to be clear brown solution. Then PIDA (55 mg, 1.0 equiv.) was added to the mixture. After stirring for 48 h, the mixture was filtered through a pad of Celite® and concentrated. The residue was purified by silica gel column chromatography (eluent: hexane/EtOAc 50/1 to 1/2). The result of column chromatography is described below.

<Isolation scheme>

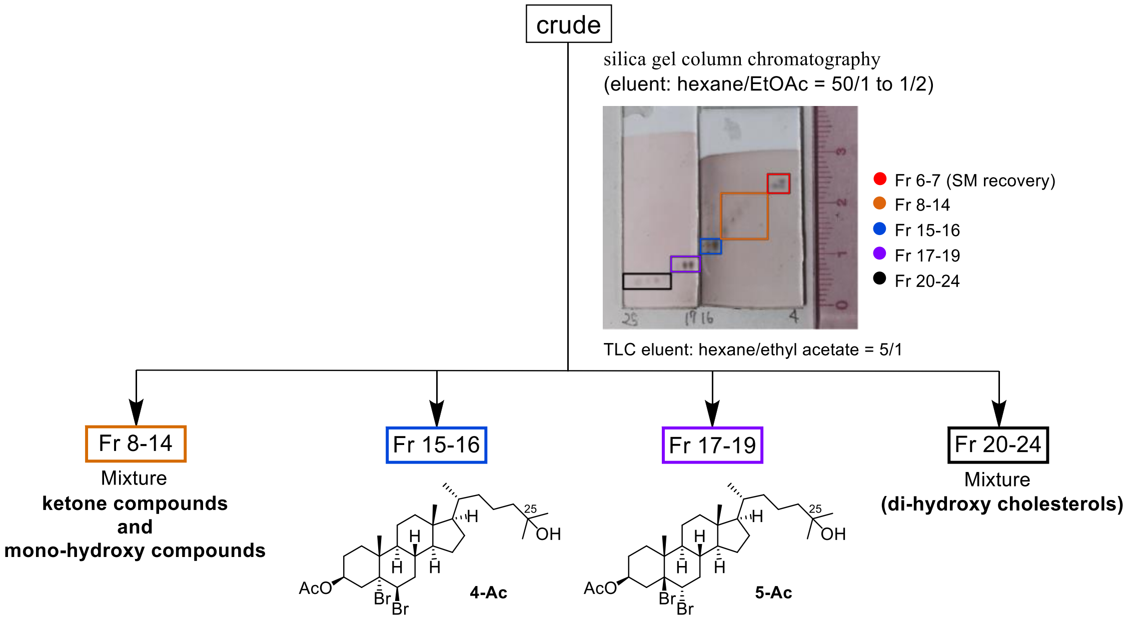

Figure S1. Purification of oxidized cholesterols (part 1)

Compound **4-Ac**:

^{1}H NMR (500 MHz, CDCl$_3$): δ 5.51–5.45 (m, 1H), 4.83 (d, *J* = 2.4 Hz, 1H), 2.70–2.64 (m, 1H), 2.59 (dd, *J* = 13.9, 10.5 Hz, 1H), 2.27 (ddd, *J* = 14.2, 5.4, 1.5 Hz, 1H), 2.04 (s, 3H), 2.07–1.81 (m, 5H), 1.73–1.55 (m, 6H), 1.46 (s, 3H), 1.44–1.23 (m, 9H), 1.21 (s, 6H), 1.18–1.01 (m, 4H), 0.93 (d, *J* = 6.3 Hz, 3H), 0.88 (m, 1H), 0.71 (s, 3H).

Compound **5-Ac**:

^{1}H NMR (500 MHz, CDCl$_3$): δ 5.15 (brs, 1H), 4.86 (dd, *J* = 12.7, 4.9 Hz, 1H), 2.86 (brd, *J* = 17.1 Hz, 1H), 2.37 (dd, *J* = 17.1, 4.4 Hz, 1H), 2.23 (ddd, *J* = 13.2, 5.4, 3.9 Hz, 1H), 2.11 (dd, *J* = 14.2, 4.4 Hz, 1H), 2.07 (s, 3H), 2.01 (m, 1H), 1.87–1.81 (m, 1H), 1.71–1.27 (m, 21H), 1.24 (s, 3H), 1.21 (s, 6H), 1.18-1.02 (m, 6H), 0.91 (d, *J* = 6.3 Hz, 3H), 0.89–0.85 (m, 1H), 0.65 (s, 3H)

[Debromination using Zinc Powder]

General Procedure A: To a solution of oxidized cholesterol (1.0 equiv.) and AcOH (3.6 equiv.) in Et_2O (56 mM) was added activated zinc powder (9 equiv.) After stirring vigorously for 4.5 h at room temperature, the mixture was filtered through a pad of Celite® and washed with saturated aqueous $NaHCO_3$ and the separated water layer was extracted with Et_2O (3 x 20 mL). The combined organic layers were dried over Na_2SO_4, filtered, and concentrated. The residue was purified by silica gel column chromatography to give de-dibrominated oxidized cholesterol.

Compound **25**

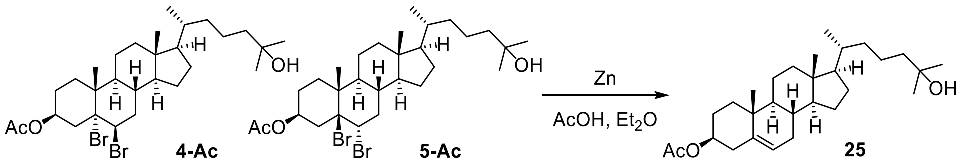

General Procedure A was followed for compound **4-Ac** (17 mg, 28 µmol) and **5-Ac** (11 mg, 18 µmol), which was converted to **25** (12 mg, 93%) and **25** (5.3 mg, 66%), respectively.

^{1}H NMR (500 MHz, CDCl$_3$): δ 5.37 (d, *J* = 4.9 Hz, 1H), 4.64–4.57 (m, 1H), 2.34–2.28 (m, 2H), 2.05–1.93 (m, 5H), 1.89–1.78 (m, 3H), 1.63–1.32 (m, 10Hs), 1.30–1.22 (m, 2H), 1.21 (s, 6H), 1.20–1.03 (m, 7H), 1.02 (s, 3H), 1.00–0.95 (m, 1H), 0.93 (d, *J* = 6.8 Hz, 3H), 0.91–0.86 (m, 1H), 0.68 (s, 3H)

^{13}C NMR (126 MHz, CDCl$_3$): δ 170.7, 139.8, 122.8, 74.1, 71.3, 56.8, 56.2, 50.2, 44.6, 42.5, 39.9, 38.3, 37.1, 36.7, 36.6, 35.9, 32.0, 32.0, 29.5, 29.4, 28.4, 27.9, 24.4, 21.6, 21.2, 20.9, 19.5, 18.8, 12.0

MS-ESI (m/z): $[M+Na]^+$ calcd for $C_{29}H_{48}O_3Na$ 467.3496, found 467.3565.

2-2: Preparation and oxidation of 3-Bz-cholesterols

Compound **1-Bz**

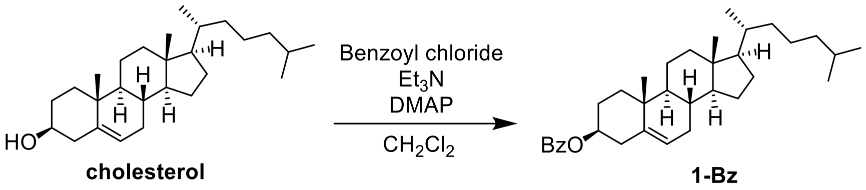

To a solution of cholesterol (3.00 g, 7.76 mmol, 1.0 equiv.) and DMAP (94.8 mg, 0.776 mmol, 0.1 equiv.) in CH_2Cl_2 (39 mL, 0.2 M) was added Et_3N (3.3 mL, 23.3 mmol, 3.0 equiv.) and benzoyl chloride (1.4 mL, 11.6 mmol, 1.5 equiv.) at 0 ºC. After the mixture was stirred for 4.5 h at room temperature, more DMAP (94.8 mg, 0.776 mmol, 0.1 equiv.) was added. The mixture was stirred for total 23.5 h. The mixture was quenched with saturated aqueous $NaHCO_3$ at 0°C and extracted with CH_2Cl_2 (3 x 30 mL). The combined organic layers were washed with aqueous 1 N HCl and aqueous saturated $NaHCO_3$ and the separated water layer was extracted with CH_2Cl_2. The combined organic layers were dried over Na_2SO_4, filtered, and concentrated. The residue was purified by silica gel column chromatography (eluent: hexane/EtOAc 50/1 to 5/1) to give **1-Bz** (3.74 g, 7.62 mmol, 98%) as a white solid.

^{1}H NMR (500 MHz,CDCl$_3$): δ 8.04 (dd, *J* = 8.4, 1.4 Hz, 2H), 7.54 (tt, *J* = 7.4, 1.4 Hz, 1H), 7.43 (t, *J* = 7.6 Hz, 2H), 5.42 (d, *J* = 3.7 Hz, 1H), 4.86 (m , 1H), 2.47 (d, *J* = 7.6 Hz, 2H), 2.04–1.97 (m, 3H), 1.92 (dt, *J* = 13.3, 3.4 Hz, 1H), 1.88–1.80 (m, 1H), 1.78–1.69 (m, 1H), 1.62–1.44 (m, 7H), 1.41–1.30 (m, 3H), 1.28–1.06 (m, 7H), 1.07 (s, 3H), 1.05–0.97 (m, 3H), 0.92 (d, *J* = 6.7 Hz, 3H), 0.87 (dd, *J* = 6.6, 2.3 Hz, 6H), 0.69 (s, 3H).

Compound **2-Bz** and **3-Bz**

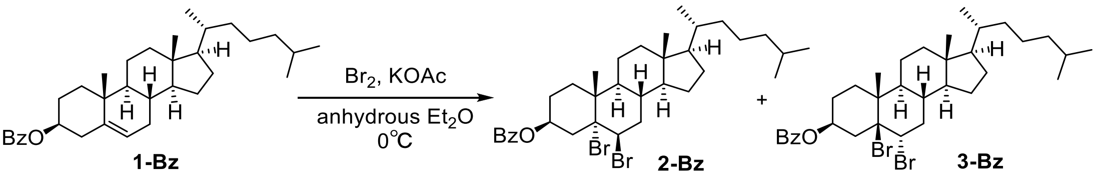

To a solution of **1-Bz** (3.63 g, 7.40 mmol, equiv.) in anhydrous Et_2O (90 mL, 82.5 mM) was added a solution of KOAc (5.6 g) in glacial acetic acid (56 mL) at 0°C, and then added a solution of Br_2 (3.8 mL, 74 mmol, 10 equiv.) in glacial acetic acid (28 mL) at 0°C. After stirring for 3 h at 0°C, the reaction was allowed to worm to room temperature and stirred overnight. The resulting mixture was quenched with aqueous 1 M $Na_2S_2O_3$ at room temperature and extracted with diethyl ether (3 x 30 mL). The combined organic layer was washed with aqueous 1 M $Na_2S_2O_3$ (30 mL), followed by aqueous 1 M K_2CO_3 (3 x 50 mL), dried over Na_2SO_4, filtered, and concentrated. The residue was purified by MPLC (Yamazen, Ultrapack 40D eluent: hexane/EtOAc = 98/2) to give dibromide **2-Bz** (2.06 mg, 3.2 mmol, 43%) and the isomer **3-Bz** (1.03 mg, 1.6 mmol, 22%).

Compound **2-Bz**:
^{1}H NMR (500 MHz, CDCl$_3$): δ 8.06 (dd, *J* = 8.4, 1.4 Hz, 2H), 7.56 (tt, *J* = 7.4, 1.5 Hz, 1H),7.44 (t, *J* = 7.6 Hz, 2H), 5.75 (m, 1H), 4.87 (d, *J* = 2.4 Hz, 1H), 2.76–2.66 (m, 2H), 2.42 (ddd, *J* = 14.0, 5.3, 1.6 Hz, 1H), 2.12–2.00 (m, 3H), 1.94–1.77 (m, 4H), 1.69–1.62 (m, 3H), 1.52 (s, 3H), 1.50–1.46 (m, 1H), 1.38–1.20 (m, 8H), 1.17–1.00 (m, 6H), 0.92 (d, *J* = 6.4 Hz, 3H), 0.87 (dd, *J* = 6.6, 2.3 Hz, 6H), 0.72 (s, 3H).

Compound **3-Bz**:
¹H NMR (500 MHz,CDCl₃): δ 8.18 (dd, *J* = 8.2, 1.2 Hz, 2H), 7.54 (tt, *J* = 7.4, 1.4 Hz, 1H), 7.43 (t, *J* = 7.8 Hz, 2H), 5.48 (d, *J* = 2.4 Hz, 1H), 4.92 (dd, *J* = 12.7, 5.0 Hz, 1H), 2.95 (d, *J* = 17.1 Hz, 1H), 2.55 (dd, *J* = 16.8, 4.6 Hz, 1H), 2.29–2.18 (m, 2H), 2.07–2.01 (m, 1H), 1.87 (s, 1H), 1.79–1.69 (m, 3H), 1.62–1.56 (m, 3H), 1.52–1.43 (m, 5H), 1.41–1.27 (m, 5H), 1.28 (s, 3H), 1.18–0.99 (m, 9H), 0.90 (d, *J* = 6.7 Hz, 3H), 0.87 (dd, *J* = 6.6, 2.3 Hz, 6H), 0.66 (s, 3H).

[Ru-catalyzed oxidation of compound 2-Bz followed by debromination]

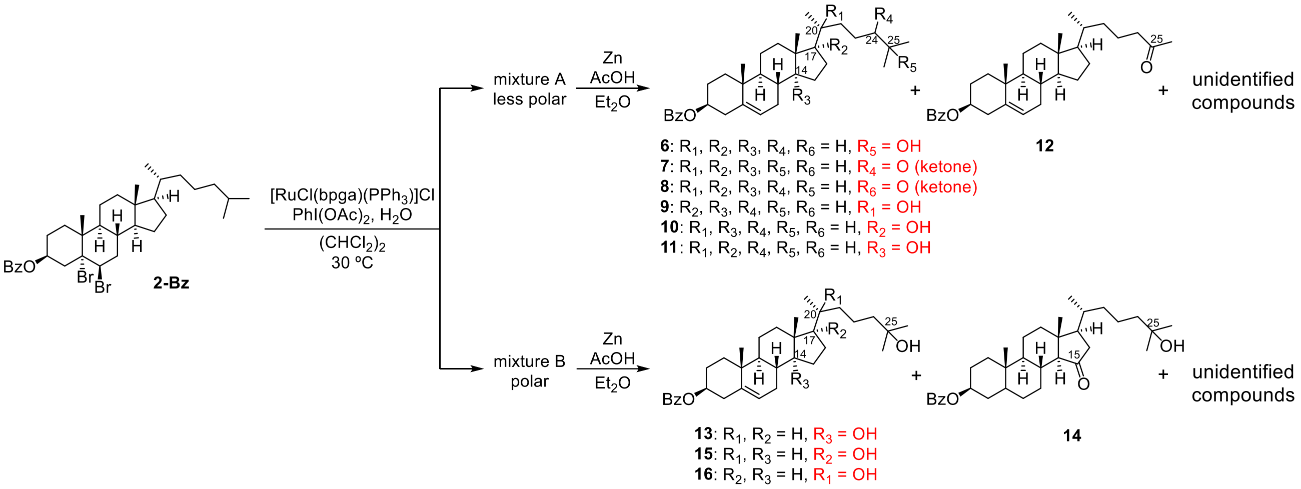

To a 10 mL screw-cap vial was added **2-Bz** (400 mg, 615 µmol, 1.0 equiv.), iodobenzene diacetate (PIDA, 198 mg, 615 µmol, 1.0 equiv.), H_2O (30 µL), $(CHCl_2)_2$ (1.5 mL), and a magnetic stir bar. The vial was placed on an aluminum block at 30 °C, added [RuCl(bpga)(PPh_3)]Cl (9.6 mg, 12 µmol, 2 mol%), and sealed with a screw cap. After stirring for 24 h, the suspended brown mixture was turned to be clear brown solution. Then PIDA (198 mg, 1.0 equiv.) was added to the mixture. After stirring for 41 h, the mixture was filtered through a pad of Celite® and concentrated. The residue was purified by silica gel column chromatography (eluent: hexane/EtOAc 50/1 to 1/2). to give less polar mixture **A** (263 mg), polar mixture **B** (95.3 mg), and a mixture of recovered **2-Bz** and **3-Bz** (111.4 mg, 28%, **2-Bz**:**3-Bz** = 1:3).

This reaction was performed in 917 mg scale and gave **4-Bz** (101 mg, 0.15 mmol, 11%), the isomer **5-Bz** (220 mg, 0.33 mmol, 24%), less polar mixture **A'** (200 mg), polar mixture **B'** (215 mg), and recovered **2-Bz** (204 mg, 0.31 mmol, 22%)

Compound **4-Bz**:

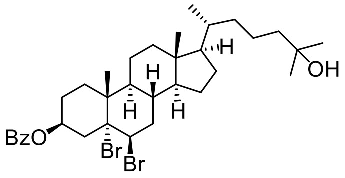

4-Bz

¹H NMR (500 MHz,CDCl₃): δ 8.06 (d, *J* = 8.3 Hz, 2H), 7.56 (t, *J* = 7.1 Hz, 1H), 7.44 (t, *J* = 7.6 Hz, 2H), 5.78–5.72 (m, 1H), 4.87 (s, 1H), 2.76–2.67 (m, 2H), 2.43 (dd, J = 14.2, 4.9 Hz, 1H), 2.11–2.01 (m, 3H), 1.94–1.77 (m, 4H), 1.68–1.55 (m, 4H), 1.52 (s, 3H), 1.50–1.28 (m, 9H), 1.22 (s, 6H), 1.19–1.02 (m, 4H), 0.94 (d, *J* = 6.3 Hz, 3H), 0.73 (s, 3H).
¹³C NMR (126 MHz,CDCl₃): δ 166.1, 133.0, 130.7, 129.8 (2C), 128.5 (2C), 88.3, 72.9, 71.2, 56.3, 56.2, 55.3, 47.4, 44.6, 42.9, 42.2, 42.1, 39.8, 37.4, 36.7, 36.5, 35.9, 31.0, 29.5, 29.4, 28.3, 26.5, 24.2, 21.5, 21.0, 20.4, 18.8, 12.4.

Compound **5-Bz**:

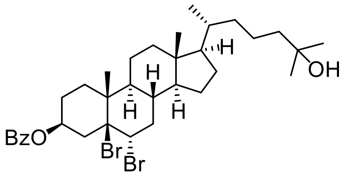

5-Bz

^{1}H NMR (500 MHz,CDCl$_3$): δ 8.18 (d, *J* = 7.8 Hz, 2H), 7.54 (t, *J* = 7.3 Hz, 1H), 7.43 (t, *J* = 7.6 Hz, 2H), 5.48 (s, 1H), 4.92 (dd, *J* = 12.7, 4.9 Hz, 1H), 2.95 (d, *J* = 17.1 Hz, 1H), 2.55 (dd, *J* = 16.8, 4.1 Hz, 1H), 2.28–2.17 (m, 2H), 2.03 (d, *J* = 13.7 Hz, 1H), 1.87–1.82 (m, 1H), 1.79–1.69 (m, 3H), 1.64–1.54 (m, 3H), 1.50–1.35 (m, 8H), 1.29 (s, 3H), 1.29–1.24 (m, 2H), 1.22 (s, 6H), 1.19–1.04 (m, 5H), 0.93 (d, *J* = 6.3 Hz, 3H), 0.67 (s, 3H).

^{13}C NMR (126 MHz,CDCl$_3$): δ 166.2, 132.9, 130.8, 130.2 (2C), 128.4 (2C), 79.0, 71.2, 68.4, 64.2, 56.2, 56.1, 45.1, 44.5, 42.7, 41.8, 41.8, 39.8, 36.6, 36.5, 35.8, 35.1, 29.5, 29.4, 29.3, 28.3, 24.4, 24.2, 22.6, 21.4, 20.9, 18.8, 12.0.

General Procedure A was followed for the less polar mixture **A** (189 mg, 280 μmol, 1.0 equiv.) and the polar mixture **B** (190 mg, 280 μmol) to give 126 mg of crude mixture and 129 mg of crude mixture, respectively.

The crude material derived from mixture **A** was purified by silica gel column chromatography (eluent: hexane/EtOAc 50/1 to 1/1) (For further purification, see Figure S2).

The crude material derived from mixture **B** was purified by silica gel column chromatography (eluent: hexane/EtOAc 82/18 to 50/50) (For further purification, see Figure S9).

<Isolation scheme>

・Oxidized compounds derived from the crude mixture A (less polar)

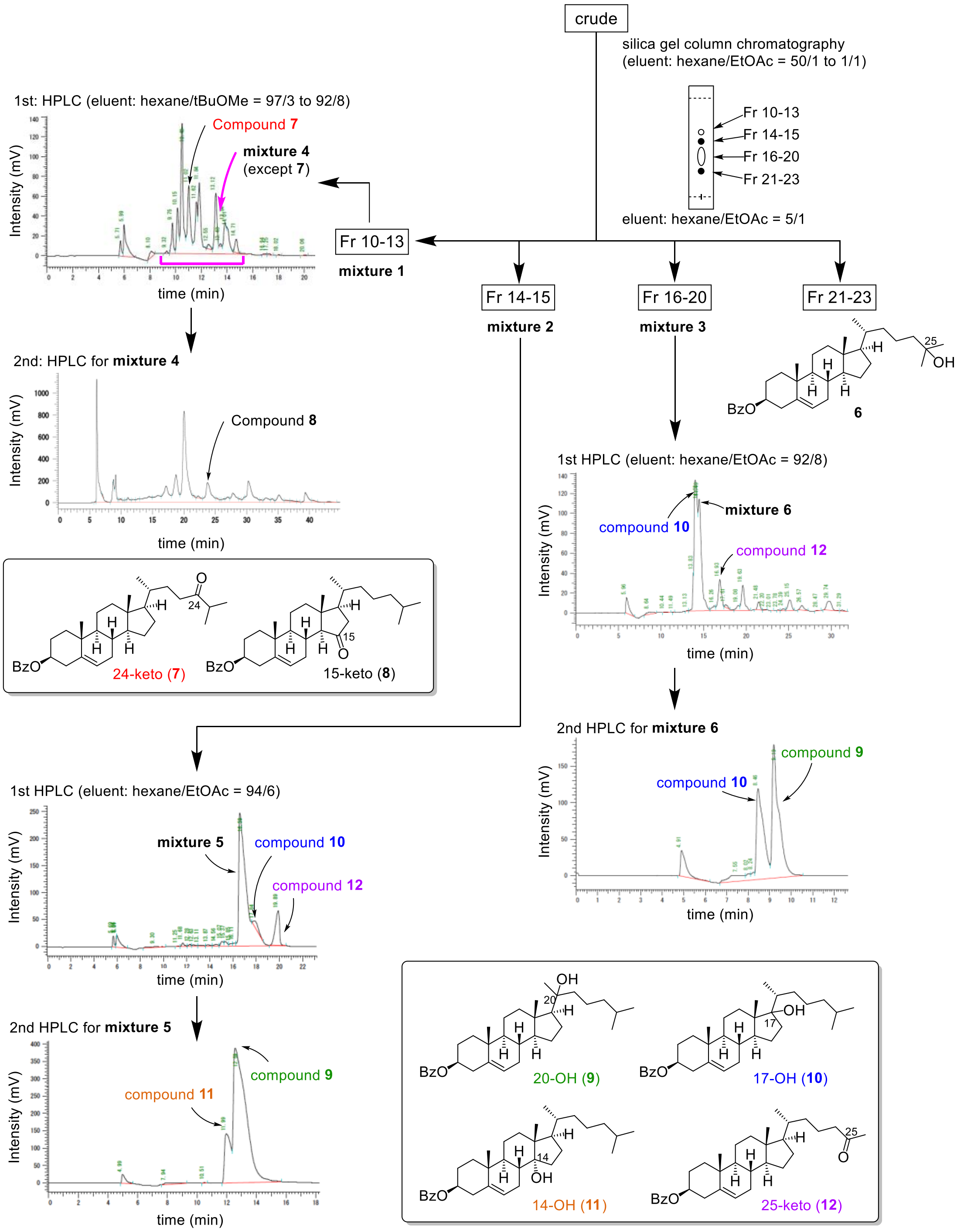

Figure S2. Purification of oxidized cholesterols (part 2)

After the silica gel column chromatography, the combined fractions 21-23 were concentrated to give **6** (79.4 mg, 26%). For fractions 10-13, further purification was carried out by HPLC (Figure S2) to give **7** (2.0 mg, 0.99%). For fractions 14-15, further purification was carried out by HPLC to give **9** (10.5 mg, 3.5%), **10** (11.3 mg, 3.7%), **11** (1.0 mg, 0.33%), and **12** (3.2 mg, 1.1%).

Compound **6** (3-*O*-Bz-25-hydroxy cholesterol)

^{1}H NMR (500 MHz, $CDCl_3$): δ 8.04 (m, 2H), 7.54 m, 1H), 7.43 (m, 2H), 5.42 (d, *J* = 3.9 Hz, 1H), 4.90–4.83 (m, 1H), 2.47 (d, *J* = 7.8 Hz, 2H), 2.04–1.98 (m, 3H), 1.92 (dt, *J* = 13.7, 3.4 Hz, 1H), 1.86–1.80 (m, 1H), 1.78–1.70 (m, 1H), 1.60–1.36 (m, 10H), 1.30–1.22 (m, 4H), 1.22 (s, 6H), 1.19–1.15 (m, 1H), 1.07 (s, 3H), 1.13–0.97 (m, 4H), 0.94 (d, *J* = 6.3 Hz, 3H), 0.69 (s, 3H).

^{13}C NMR (125 MHz, $CDCl_3$): δ 166.2, 139.8, 132.9, 131.0, 129.7 (2C), 128.4 (2C), 122.9, 74.7, 71.3, 56.8, 56.2, 50.2, 44.6, 42.5, 39.9, 38.4, 37.2, 36.8, 36.6, 35.9, 32.1, 32.1, 29.5, 29.4, 28.4, 28.0, 24.4, 21.2, 21.0, 19.5, 18.9, 12.0.

MS-ESI (m/z): $[M+Na]^+$ calcd for $C_{34}H_{50}O_3Na$ 529.3652, found 529.3558.

Compound **7**:

^{1}H NMR (500 MHz, $CDCl_3$): δ 8.04 (m, 2H), 7.54 (m, 1H), 7.43 (m, 2H), 5.42 (d, *J* = 3.7 Hz, 1H), 4.89–4.83 (m, 1H), 2.64–2.58 (m, 1H), 2.51–2.44 (m, 3H), 2.39–2.33 (m, 1H), 2.03–1.99 (m, 3H), 1.94–1.89 (m, 1H), 1.88–1.84 (m, 1H), 1.78–1.70 (m, 2H), 1.63–1.59 (m, 1H), 1.54–1.46 (m, 4H), 1.44–1.16 (m, 5H), 1.09 (d, *J* = 7.0 Hz, 6H), 1.07 (s, 3H), 1.14–0.96 (m, 4H), 0.92 (d, *J* = 6.4 Hz, 3H), 0.69 (s, 3H).

^{13}C NMR (125 MHz, $CDCl_3$): δ 215.7, 166.2, 139.8, 132.9, 131.0, 129.7 (2C), 128.4 (2C), 122.9, 77.4, 77.2, 76.9, 74.7, 56.8, 56.0, 50.2, 42.5, 41.0, 39.9, 38.4, 37.4, 37.2, 36.8, 35.5, 32.1, 32.0, 30.0, 28.3, 28.0, 24.4, 21.2, 19.5, 18.7, 18.5, 18.5, 12.0.

MS-ESI (m/z): $[M+Na]^+$ calcd for $C_{34}H_{48}O_3Na$ 527.3496, found 527.3441.

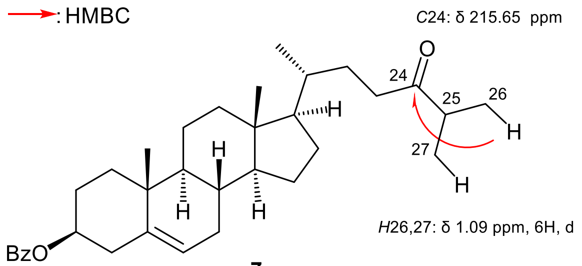

7

Figure S3. Characteristic HMBC correlation for structural determination of 7

Compound **8**:

^{1}H NMR (500 MHz,$CDCl_3$): δ 8.06–8.02 (m, 2H), 7.57–7.52 (m, 1H), 7.46–7.41 (m, 2H), 5.43 (brd, *J* = 4.9 Hz, 1H), 4.89–4.81 (m, 1H), 2.86 (ddd, *J* = 17.7, 4.6, 4.6 Hz, 1H), 2.49–2.41 (m, 3H), 2.19–2.14 (m, 1H), 2.05–1.98 (m, 1H), 1.92 (ddd, *J* = 13.3, 3.4, 3.4 Hz, 1H), 1.87–1.68 (m, 4H), 1.64–0.98 (m, 15H), 1.07 (s, 3H), 1.01 (d, *J* = 6.7 Hz, 3H), 0.872 (d, *J* = 6.7 Hz, 3H), 0.868 (d, *J* = 6.7 Hz, 3H), 0.78 (s, 3H).

^{13}C NMR (125 MHz, $CDCl_3$): δ 216.2, 166.2, 139.1, 132.9, 130.9, 129.7 (2C), 128.4 (2C), 122.8, 74.6, 66.8, 51.8, 49.7, 42.3, 41.9, 39.8, 39.5, 38.2, 37.2, 36.5, 36.2, 35.5, 31.3, 28.13, 28.10, 28.0, 23.9, 22.9, 22.7, 20.9, 19.3, 19.2, 12.9.

MS-ESI (m/z): $[M+Na]^+$ calcd for $C_{34}H_{48}O_3Na$ 527.3496, found 527.3421.

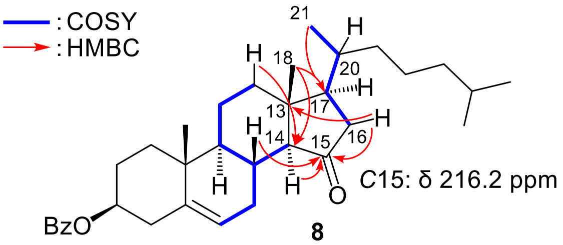

Figure S4. Characteristic HMBC and COSY correlations for structural determination of 8

Compound **9**:

^{1}H NMR (500 MHz, CDCl$_3$): δ 8.04 (m, 2H), 7.54 (m, 1H), 7.43 (m, 2H), 5.42 (d, *J* = 3.7 Hz, 1H), 4.90–4.82 (m, 1H), 2.47 (d, *J* = 7.6 Hz, 2H), 2.11 (dt, *J* = 12.3, 3.4 Hz, 1H), 2.02–1.98 (m, 2H), 1.92 (dt, *J* = 13.5, 3.5 Hz, 1H), 1.80–1.61 (m, 5H), 1.57–1.39 (m, 6H), 1.28 (s, 3H), 1.34–1.12 (m, 8H), 1.07 (d, *J* = 5.5 Hz, 3H), 1.05–0.96 (m, 2H), 0.88 (s, 3H), 0.88 (d, *J* = 6.7 Hz, 6H).

^{13}C NMR (125 MHz, CDCl$_3$): δ 166.2, 139.8, 132.9, 131.0, 129.7 (2C), 128.4 (2C), 122.8, 75.4, 74.7, 57.8, 57.0, 50.1, 44.4, 42.8, 40.2, 39.8, 38.4, 37.2, 36.8, 32.0, 31.5, 28.1, 28.0, 26.6, 23.9, 22.9, 22.7, 22.5, 22.2, 21.1, 19.5, 13.8.

MS-ESI (m/z): $[M+Na]^+$ calcd for $C_{34}H_{50}O_3Na$ 529.3652, found 529.3597.

$[\alpha]_D^{27}$ –27.780 (c = 2.7, CHCl$_3$)

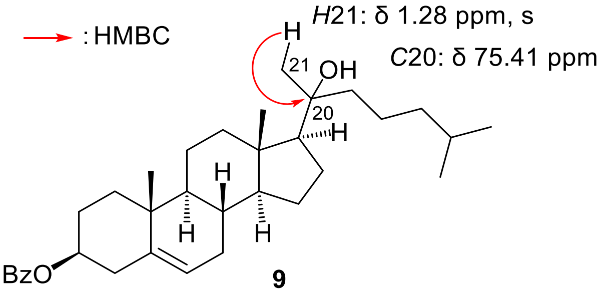

Figure S5. Characteristic HMBC correlation for structural determination of 9

Compound **10**:

^{1}H NMR (500 MHz, CDCl$_3$): δ 8.04 (m, 2H), 7.54 (m, 1H), 7.43 (m, 2H), 5.43 (d, *J* = 4.9 Hz, 1H), 4.87–4.83 (m, 1H), 2.47 (d, *J* = 7.8 Hz, 2H), 2.02–1.99 (m, 2H), 1.92 (dt, *J* = 13.3, 3.7 Hz, 1H), 1.85–1.59 (m, 9H), 1.54–1.43 (m, 5H), 1.28–1.09 (m, 7H), 1.08 (s, 3H), 1.03 (td, *J* = 11.6, 4.6 Hz, 1H), 0.91 (d, *J* = 6.8 Hz, 3H), 0.87 (dd, *J* = 6.8, 3.0 Hz, 6H), 0.79 (s, 3H).

^{13}C NMR (125 MHz, CDCl$_3$): δ 166.1, 139.7, 132.9, 131.0, 129.7 (2C), 128.4 (2C), 122.9, 86.6, 74.7, 51.3, 49.8, 47.4, 39.9, 39.5, 38.4, 38.3, 37.2, 36.8, 32.5, 32.5, 32.4, 32.1, 28.1, 28.0, 25.8, 23.9, 23.0, 22.7, 21.0, 19.5, 14.5, 14.2.

MS-ESI (m/z): $[M+Na]^+$ calcd for $C_{34}H_{50}O_3Na$ 529.3652, found 529.3597

$[\alpha]_D^{24}$ –13.105 (c = 1.1, CHCl$_3$).

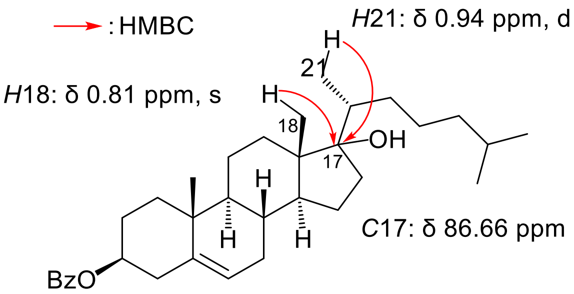

10

Figure S6. Characteristic HMBC correlations for structural determination of 10

Compound **11**:

^{1}H NMR (500 MHz, CDCl$_3$): δ 8.04 (m, 2H), 7.54 (m, 1H), 7.43 (m, 2H), 5.46 (d, *J* = 2.9 Hz, 1H), 4.89–4.82 (m, 1H), 2.49–2.46 (m, 2H), 2.17–2.11 (m, 1H), 2.02–1.96 (m, 2H), 1.95-1.83 (m, 3H), 1.79–1.69 (m, 4H), 1.54–1.23 (m, 11H), 1.19–1.12 (m, 3H), 1.09 (s, 3H), 1.06–1.00 (m, 1H), 0.90 (d, *J* = 6.4 Hz, 3H), 0.87 (dd, *J* = 6.7, 2.1 Hz, 6H), 0.84 (s, 3H).

^{13}C NMR (125 MHz, CDCl$_3$): δ 166.1, 139.3, 132.9, 131.0, 129.7 (2C), 128.4 (2C), 122.9, 85.3, 74.6, 51.3, 46.6, 43.4, 39.7, 38.3, 37.2, 37.1, 36.5, 35.8, 34.9, 32.8, 31.9, 28.2, 28.0, 27.3, 26.1, 24.2, 23.0, 22.7, 20.0, 19.4, 18.8, 15.6.

MS-ESI (m/z): [M+Na]$^+$ calcd for $C_{34}H_{50}O_3Na$ 529.3652, found 529.3597.

[α]$_D^{24}$ 7.783 (c = 0.81, CHCl$_3$)

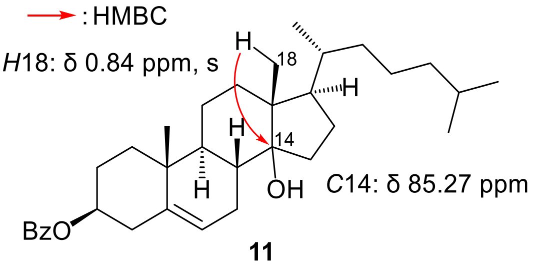

11

Figure S7. Characteristic HMBC correlations for structural determination of 11

Compound **12**:

^{1}H NMR (500 MHz, CDCl$_3$): δ 8.04 (m, 2H), 7.54 (m, 1H), 7.43 (m, 2H), 5.42 (d, *J* = 3.7 Hz, 1H), 4.89–4.83 (m, 1H), 2.46 (d, *J* = 7.6 Hz, 2H), 2.43–2.33 (m, 2H), 2.13 (s, 3H), 2.03–1.96 (m, 3H), 1.92 (dt, *J* = 13.4, 3.5 Hz, 1H), 1.87–1.77 (m, 1H), 1.75–1.72 (m, 1H), 1.70–1.32 (m, 10H), 1.28–0.93 (m, 7H), 1.07 (s, 3H), 0.94 (d, *J* = 6.7 Hz, 3H), 0.69 (s, 3H).

^{13}C NMR (125 MHz, CDCl$_3$): δ 209.6, 166.2, 139.8, 132.9, 131.0, 129.7 (2C), 128.4 (2C), 122.9, 77.4, 77.2, 76.9, 74.7, 56.8, 56.0, 50.2, 44.4, 42.5, 39.9, 38.4, 37.2, 36.8, 35.8, 35.6, 32.1, 32.0, 30.0, 28.4, 28.0, 24.4, 21.2, 20.6, 19.5, 18.8, 12.0.

MS-ESI (m/z): [M+Na]$^+$ calcd for $C_{33}H_{46}O_3Na$ 513.3339, found 513.3392.

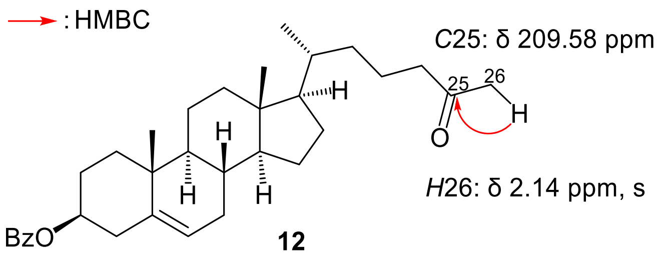

Figure S8. Characteristic HMBC correlations for structural determination of 12

<Isolation scheme>

・Oxidized compounds derived from mixture B (polar)

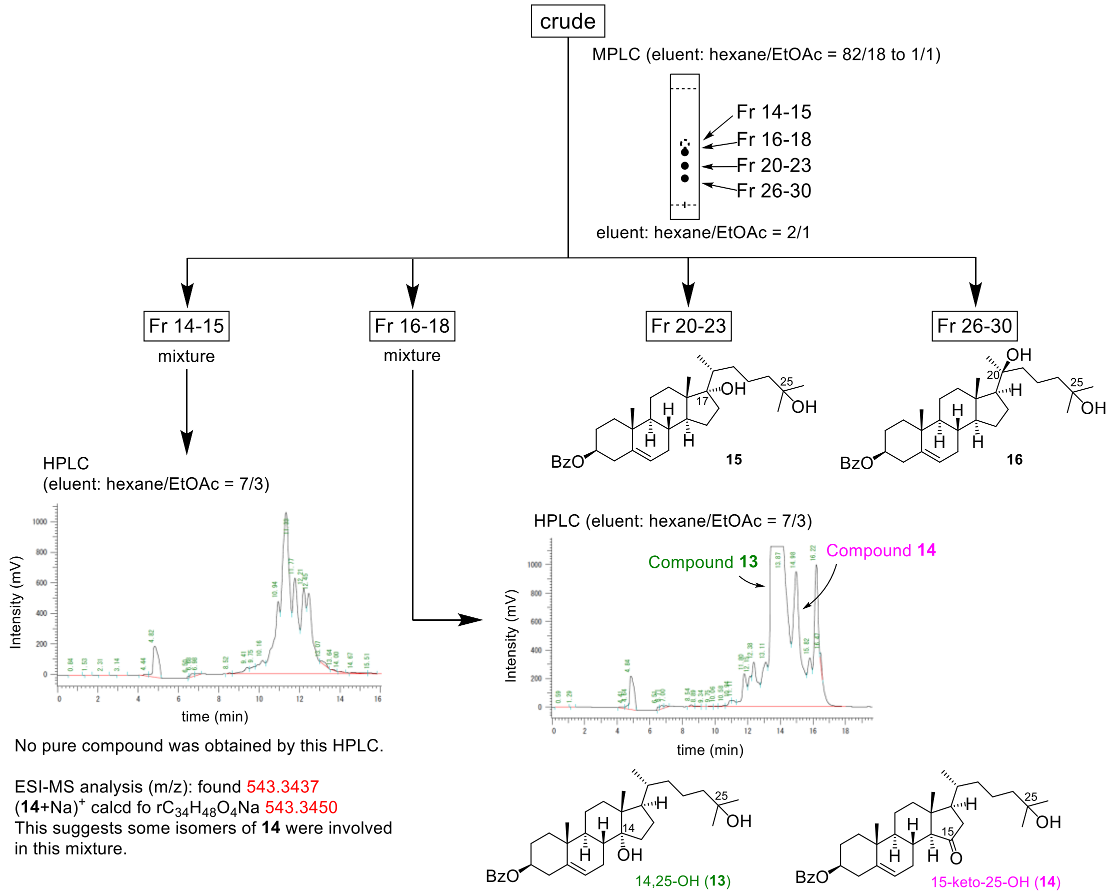

Figure S9. Purification of oxidized cholesterols (part 3)

After silica gel column chromatography, the combined fractions 20-23 were concentrated to give **15** (14.6 mg, 4.7%). The combined fractions 26-30 were concentrated to give **16** (15.7 mg, 5.0%). Further purification was carried out for the fractions 16-18 by HPLC (Senshu-Pak, PEGASIL, ø10-250, 3 mL/min, hexane/EtOAc 7/3) to give **13** (4.7 mg, 1.5%) and **14** (0.7 mg, 0.22%).

Compound **13**:
^{1}H NMR (500 MHz, CDCl$_3$): δ 8.04 (m, 2H), 7.54 (m, 1H), 7.43 (m, 2H), 5.46 (d, *J* = 2.7 Hz, 1H), 4.88–4.83 (m, 1H), 2.49–2.46 (m, 2H), 2.17–2.11 (m, 1H), 2.04–1.96 (m, 2H), 1.95–1.69 (m, 7H), 1.54–1.33 (m, 11H), 1.30–1.22 (m, 2H), 1.22 (s, 6H), 1.09 (s, 3H), 1.10–1.07 (m, 1H), 0.92 (d, *J* = 6.7 Hz, 3H), 0.84 (s, 3H).

^{13}C NMR (125 MHz, CDCl$_3$): δ 166.1, 139.3, 132.9, 131.0, 129.7 (2C), 128.4 (2C), 122.9, 85.3, 74.6, 71.3, 51.3, 46.7, 44.5, 43.4, 38.3, 37.2, 37.1, 36.8, 35.8, 34.9, 32.8, 31.9, 29.5, 29.4, 28.0, 27.3, 26.1, 21.2, 20.0, 19.4, 18.8, 15.6.
MS-ESI (m/z): [M+Na]$^+$ calcd for $C_{34}H_{50}O_4Na$ 545.3601, found 545.3642.
[α]D^{26} 7.959 (c = 0.44, $CHCl_3$)

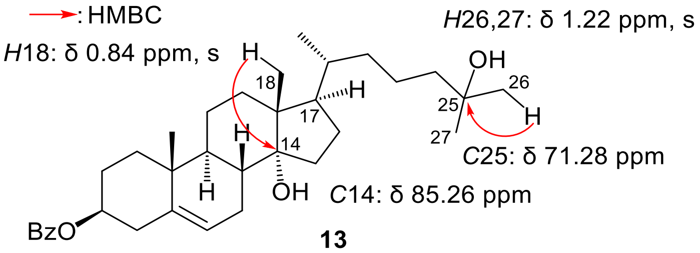

13

Figure S10. Characteristic HMBC correlations for structural determination 13

Compound **14**:
^{1}H NMR (500 MHz, CDCl$_3$): δ 8.04 (m, 2H), 7.54 (m, 1H), 7.43 (m, 2H), 5.43 (d, J = 4.0 Hz, 1H), 4.89–4.82 (m, 1H), 2.86 (dt, J = 17.7, 4.9 Hz, 1H), 2.47 (m, 3H), 2.18–2.16 (m, 1H), 2.04–2.00 (m, 1H), 1.92 (dt, J = 13.2, 3.2 Hz, 1H), 1.83–1.72 (m, 4H), 1.63–1.59 (m, 3H), 1.54–1.28 (m, 7H), 1.28–1.17 (m, 2H), 1.22 (s, 6H), 1.07 (s, 3H), 1.12–1.07 (m, 1H), 1.03 (d, J =6.4 Hz, 3H), 1.05–1.02 (m, 1H), 0.78 (s, 3H).
^{13}C NMR (125 MHz, CDCl$_3$): δ 216.0, 166.2, 139.1, 132.9, 130.9, 129.7 (2C), 128.4 (2C), 122.8, 74.6, 71.2, 66.8, 51.8, 49.6, 44.3, 42.3, 41.9, 39.8, 38.2, 37.2, 36.5, 36.4, 35.5, 31.3, 29.6, 29.4, 28.1, 28.0, 20.9, 20.8, 19.3, 19.1, 12.9.

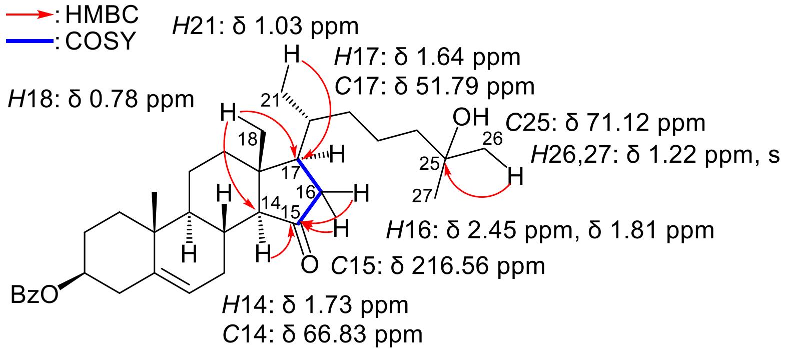

14

Figure S11. Characteristic HMBC and COSY correlations for structural determination of 14

Compound **15**:
^{1}H NMR (500 MHz, CDCl$_3$): δ 8.04 (m, 2H), 7.54 (m, 1H), 7.43 (m, 2H), 5.42 (d, J = 4.6 Hz, 1H), 4.89–4.83 (m, 1H), 2.47 (d, J = 7.6 Hz, 2H), 2.12–2.07 (m, 1H), 2.01–1.97 (m, 2H), 1.91 (dt, J = 13.4, 3.4 Hz, 1H), 1.77–1.35 (m, 15H), 1.30 (s, 3H), 1.23 (s, 6H), 1.23–1.10 (m, 3H), 1.07 (s, 3H), 1.05–0.96 (m, 2H), 0.87 (s, 3H).

^{13}C NMR (125 MHz, CDCl$_3$): δ 166.2, 139.8, 132.9, 131.0, 129.7 (2C), 128.4 (2C), 122.8, 75.4, 74.7, 71.2, 58.0, 57.0, 50.1, 44.6, 44.4, 42.8, 40.2, 38.3, 37.2, 36.8, 31.9, 31.5, 29.6, 29.4, 28.0, 26.5, 23.9, 22.6, 21.0, 19.5, 19.1, 13.8.
HRMS-ESI (m/z): [M+Na]$^+$ calcd for $C_{34}H_{50}O_4Na$ 545.3601, found 545.3628.
[α]D^{26} –25.639 (c = 3.48, $CHCl_3$)

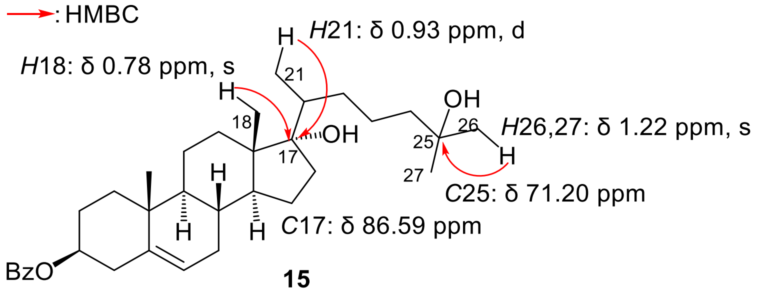

Figure S12. Characteristic HMBC correlations for structural determination of 15

Compound **16**:
^{1}H NMR (500 MHz, CDCl$_3$): δ 8.04 (m, 2H), 7.54 (m, 1H), 7.43 (m, 2H), 5.43 (d, J = 4.6 Hz, 1H), 4.90–4.83 (m, 1H), 2.47 (d, J = 7.8 Hz, 2H), 2.02–1.98 (m, 2H), 1.92 (dt, J = 13.4, 3.5 Hz, 1H), 1.85–1.77 (m, 1H), 1.75–1.67 (m, 6H), 1.65–1.38 (m, 9H), 1.25–1.20 (m, 3H), 1.22 (s, 6H), 1.13 (dd, J = 21.2, 17.2 Hz, 1H), 1.08 (s, 3H), 1.06–1.00 (m, 1H), 0.93 (d, J = 6.6 Hz, 3H), 0.79 (s, 3H).
^{13}C NMR (125 MHz, CDCl$_3$):δ 166.1, 139.7, 132.9, 131.0, 129.7 (2C), 128.4 (2C), 122.9, 86.6, 74.7, 71.2, 51.3, 49.8, 47.5, 44.4, 39.9, 38.4, 38.2, 37.2, 36.8, 32.8, 32.5, 32.4, 32.1, 29.5, 29.4, 28.0, 23.9, 22.7, 21.0, 19.5, 14.5, 14.1.
HRMS-ESI (m/z): [M+Na]$^+$ calcd for $C_{34}H_{50}O_4Na$ 545.3601, found 545.3602.
[α]D^{26} –12.662 (c = 2.61, $CHCl_3$)

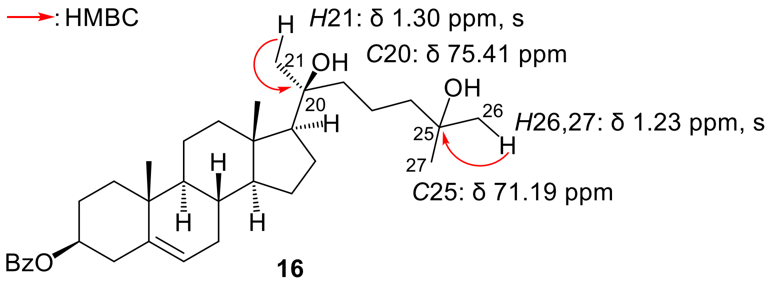

Figure S13. Characteristic HMBC correlations for structural determination of 16

[Oxidation of compound 2-Bz using Fe catalyst]

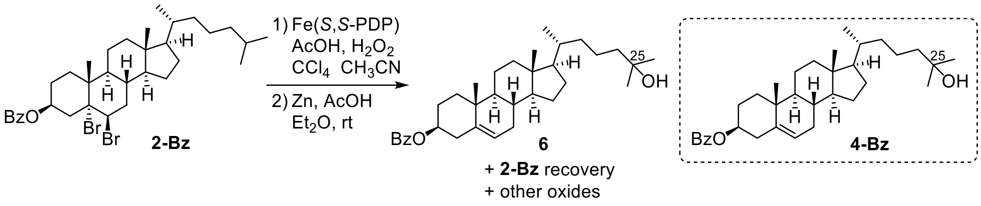

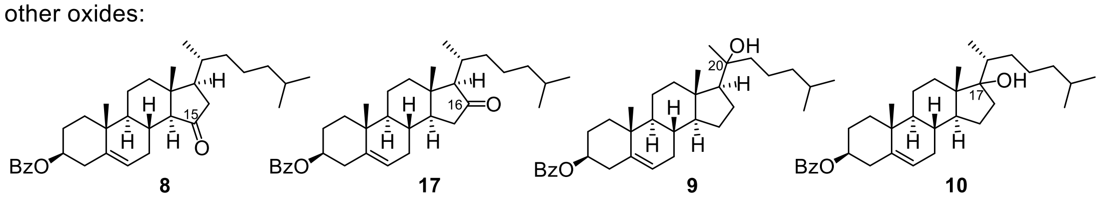

A recovery flask was charged with the following Fe(*S*,*S*-PDP) (5 mol%), **2-Bz** (980 mg, 1.51 mmol, 1.0 equiv.), CCl_4 (3.5 mL), and AcOH (43 μL, 755 μmol, 50 mol%) and a magnetic stir bar. The flask was placed on a stir plate and stirred vigorously at room temperature. A solution of H_2O_2 (50 wt% in H_2O, 56 μL, 1.8 mmol, 1.2 equiv.) in CH_3CN (1.6 mL) was added dropwise via syringe over about 120 seconds. After stirring for 10 minutes, a solution of Fe(*S*,*S*-PDP) (5 mol%) and AcOH (50 mol%) in CH_3CN (0.2 M) was added via pipette, and then H_2O_2 (50 wt.%, 1.2 equiv.) in CH_3CN (0.6 M) via syringe over about 120 seconds. Third and fourth addition was done in the same manner. After each addition, the mixture was stirred for 10 minutes. (The total reaction time was 40 minutes) The reaction was quenched with saturated aqueous $NaHCO_3$. The aqueous layer was extracted with Et_2O (3 x 20 mL). The combined organic layers were dried over Na_2SO_4, filtered, and concentrated. The residue was purified by silica gel column chromatography (eluent: hexane/EtOAc 97/3 to 75/25) to give **4-Bz** (50.1 mg, 75.2 μmol, 5%) and a mixture of other oxides and **2-Bz**. The mixture was subjected to de-bromination with Zn powder.

To a solution of the mixture of oxy-cholesterols (176 mg, 264 μmol, 1.0 equiv.) and AcOH (54 μL, 950 μmol, 3.6 equiv.) in Et_2O (4.4 mL) was added Zn powder (173 mg, 2.64 mmol, 10 equiv.). After stirring vigorously for 1 h at room temperature, AcOH (54 μL, 950 μmol, 3.6 equiv.) and Zn powder (173 mg, 2.64 mmol, 10 equiv.) was added to the mixture. After further stirring for 2 h, the mixture was filtered through a pad of Celite®. The filtrate was washed with saturated aqueous $NaHCO_3$ and the separated water layer was extracted with Et_2O (3 x 15 mL). The combined organic layers were dried over Na_2SO_4, filtered, and concentrated to give 120 mg of crude mixture. The mixture was purified by silica gel column chromatography (eluent: hexane/EtOAc 97/3 to 2/1) to give a recovery of **1-Bz** (81.2 mg, 165 μmol, 63%) and a mixture of oxidized cholesterols including **8**, **17**, **9**, and **10** (38.6 mg).

The mixture was purified by HPLC (Senshu-Pak, PEGASIL, ø10-250, 3 mL/min, hexane/*t*BuOMe 97/3 to 92/8) to give **8** (t_R 34 min, 1.8 mg, 0.24%), **17** (t_R 43 min, 1.5 mg, 0.20%), **9** (t_R 51 min, 3.5 mg, 0.46%), **10** (t_R 62 min, 4.4 mg, 0.57%).

Compound **17**:

^{1}H NMR (500 MHz,CDCl$_3$): δ 8.06–8.03 (m, 2H), 7.57–7.53 (m, 1H), 7.46–7.41 (m, 2H), 5.43 (d, *J* = 4.9 Hz, 1H), 4.91–4.83 (m, 1H), 2.50–2.46 (m, 2H), 2.22 (dd, *J* = 18.0, 7.3 Hz, 1H), 2.14–2.09 (m, 1H), 2.06–1.09 (m, 22H), 1.10 (s, 3H), 0.98 (d, *J* = 6.4 Hz, 3H), 0.872 (d, *J* = 6.7 Hz, 3H), 0.867 (d, *J* = 6.7 Hz, 3H), 0.85 (s, 3H)

^{13}C NMR (125 MHz, $CDCl_3$): δ 206.9, 166.0, 139.9, 132.8, 130.8, 129.5 (2C), 128.3 (2C), 122.1, 74.2, 67.9, 50.9, 49.9, 43.2, 39.2, 38.9, 38.0, 36.8, 35.9, 31.8, 31.3, 30.9, 30.8, 29.7, 27.9, 27.8, 25.0, 22.8, 22.6, 20.5, 19.4, 18.7, 13.7

HRMS-ESI (m/z): $[M+Na]^+$ calcd for $C_{34}H_{48}O_3Na$ 527.3496, found 527.3502.

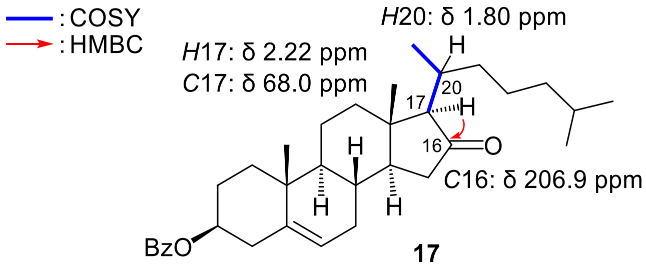

Figure S14. Characteristic HMBC and COSY correlations for structural determination of 17

2-3: Deprotection of 3-Bz-cholesterols

Compound **18** (25-hydroxy cholesterol)

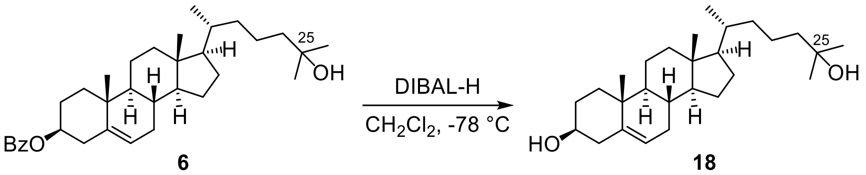

General Procedure B (Removal of Bz group with DIBAL-H): To a solution of **6** (9.2 mg, 18.2 μmol, 1.0 equiv.) in CH_2Cl_2 (360 μL) was added 1 M DIBAL-H solution in toluene (90 μL, 90 μmol, 4 equiv.) at –78 °C. After stirring for 1 h, the solution was treated with aqueous saturated Rochelle salt (3 mL) and stirred for 30 min at room temperature. The water layer was extracted with AcOEt (2 x 5 mL). The combined organic layers were dried over Na_2SO_4, filtered, and concentrated. The residue was purified by silica gel column chromatography (eluent: hexane/EtOAc 5/1 to 1/1) to give **18** (6.6 mg, 90%).

^{1}H NMR (500 MHz, $CDCl_3$): δ 5.35 (d, J = 5.2 Hz, 1H), 3.55–3.49 (m, 1H), 2.31–2.20 (m, 2H), 2.04–1.94 (m, 2H), 1.86–1.79 (m, 3H), 1.61–1.33 (m, 12H), 1.30–1.18 (m, 2H), 1.21 (s, 6H), 1.16–0.97 (m, 5H), 1.01 (s, 3H), 0.93 (d, J = 6.7 Hz, 3H), 0.95–0.90 (m, 1H), 0.68 (s, 3H)
^{13}C NMR (125 MHz, $CDCl_3$):δ 140.9, 121.8, 72.0, 71.3, 56.9, 56.2, 50.3, 44.6, 42.5, 42.5, 39.9, 37.4, 36.7, 36.6, 35.9, 32.1 (2C), 31.8, 29.5, 29.3, 28.4, 24.4, 21.2, 20.9, 19.6, 18.8, 12.0

Compound **19** (20-hydroxy cholesterol)

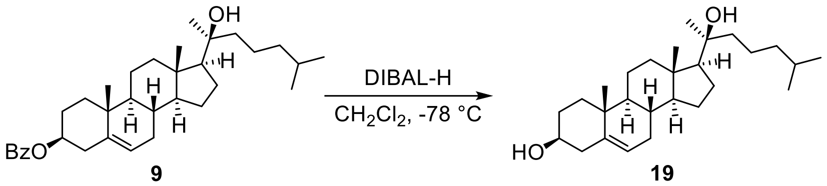

General Procedure B was followed for compound **9** (10.9 mg, 21.5 μmol). The crude mixture was purified by silica gel column chromatography (eluent: hexane/EtOAc 2/1) followed by GPC (eluent: chloroform) to give **19** (7.2 mg, 83%).

^{1}H NMR (500 MHz, $CDCl_3$): δ 5.35 (d, J = 5.4 Hz, 1H), 3.53 (s, 1H), 2.32–2.23 (m, 2H), 2.09 (d, J = 12.2 Hz, 1H), 1.98 (d, J = 17.1 Hz, 1H), 1.86–1.83 (m, 2H), 1.77–1.62 (m, 3H), 1.52–1.40 (m, 7H), 1.27 (s, 3H), 1.34–1.07 (m, 9H), 1.05–0.97 (m, 1H), 1.01 (s, 3H), 0.95–0.90 (m, 1H), 0.87 (d, J = 6.8 Hz, 6H), 0.87(s, 3H)
^{13}C NMR (126 MHz, $CDCl_3$): δ 140.9, 121.8, 75.4, 71.9, 57.9, 57.0, 50.2, 44.3, 42.8, 42.4, 40.3, 39.8, 37.4, 36.7, 31.9, 31.8, 31.5, 28.1, 26.6, 23.9, 22.9, 22.7, 22.5, 22.2, 21.1, 19.5, 13.8

Compound **20** (17-hydroxy cholesterol)

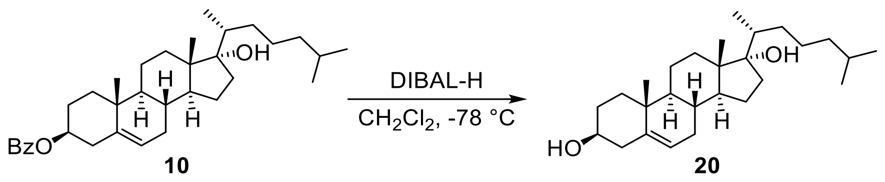

General Procedure B was followed for compound **10** (4.0 mg, 7.9 μmol). The crude mixture was purified by silica gel column chromatography (eluent: hexane/EtOAc 5/1 to 0/1) to give **20** (2.8 mg, 88%).

^{1}H NMR (500 MHz, CDCl$_3$): δ 5.35 (d, *J* = 5.2 Hz, 1H), 3.53 (m, 1H), 2.31–2.23 (m, 2H), 1.98 (dd, *J* = 17.4, 2.7 Hz, 1H), 1.87–1.79 (m, 3H), 1.72–1.60 (m, 7H), 1.54–1.40 (m, 6H), 1.19–1.06 (m, 6H), 1.02 (s, 3H), 0.96 (td, *J* = 11.6, 4.4 Hz, 1H), 0.90 (d, *J* = 6.7 Hz, 3H), 0.87 (dd, *J* = 6.7, 2.7 Hz, 6H), 0.77 (s, 3H)
^{13}C NMR (126 MHz, CDCl$_3$): δ 140.8, 121.9, 86.7, 71.9, 51.3, 49.9, 47.4, 42.4, 39.9, 39.5, 38.2, 37.4, 36.6, 32.5, 32.5, 32.4, 32.1, 31.8, 28.1, 25.8, 23.9, 22.9, 22.7, 21.0, 19.5, 14.5, 14.2

Compound **21** (14-hydroxy cholesterol)

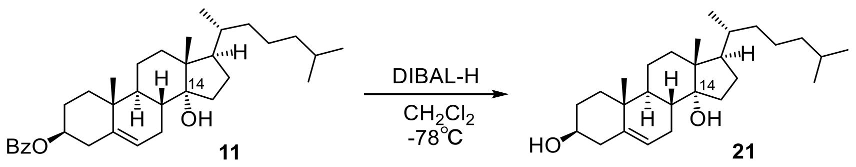

General Procedure B was followed for compound **11** (8.1 mg, 16.0 μmol). The crude mixture was purified by silica gel column chromatography (eluent: hexane/EtOAc 5/1 to 1/1) to give **20** (4.0 mg, 62%).

^{1}H NMR (500 MHz,CDCl$_3$):δ 5.38 (s, 1H), 3.53–3.50 (m, 1H), 2.32–2.20 (m, 2H), 2.13–2.07 (m, 1H), 2.01–1.93 (m, 1H), 1.89–1.83 (m, 4H), 1.73 (dd, *J* = 22.0, 8.8 Hz, 3H), 1.56–1.32 (m, 12H), 1.15–1.12 (m, 4H), 1.03 (s, 3H), 0.90 (d, *J* = 6.8 Hz, 3H), 0.87 (dd, *J* = 6.8, 2.0 Hz, 6H), 0.83 (s, 3H)
^{13}C NMR (125 MHz, CDCl$_3$): δ 140.3, 121.9, 85.3, 71.8, 51.3, 46.6, 43.5, 42.4, 39.7, 37.5, 36.9, 36.5, 35.8, 34.9, 32.7, 31.9, 31.8, 28.2, 27.3, 26.0, 24.2, 23.0, 22.7, 20.0, 19.4, 18.8, 15.6

Compound **22** (14,25-dihydroxy cholesterol)

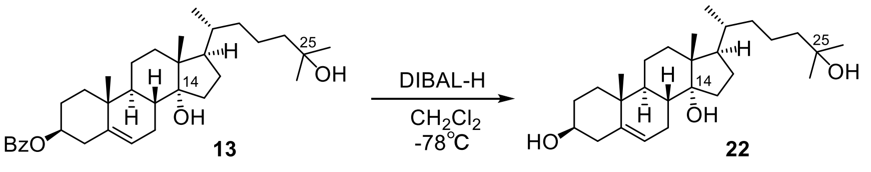

General Procedure B was followed for compound **13** (8.8 mg, 16.8 μmol). The crude mixture was purified by silica gel column chromatography (eluent: hexane/EtOAc 3/1 to 0/1) to give **22** (6.5 mg, 91%).

^{1}H NMR (500 MHz,CDCl$_3$): δ 5.38 (s, 1H), 3.54–3.50 (m, 1H), 2.32–2.20 (m, 2H), 2.10–2.06 (m, 1H), 1.99–1.95 (m, 1H), 1.89–1.79 (m, 4H), 1.78–1.70 (m, 3H), 1.51–1.32 (m, 12H), 1.21 (s, 6H), 1.27–1.07 (m, 3H), 1.03 (s, 3H), 0.91 (d, *J* = 6.3 Hz, 3H), 0.83 (s, 3H)
^{13}C NMR (125 MHz, CDCl$_3$): δ 140.3, 121.9, 85.3, 71.8, 71.3, 51.3, 46.7, 44.5, 43.5, 42.4, 37.5, 36.9, 36.8, 35.8, 34.9, 32.7, 31.9, 31.8, 29.5, 29.4, 27.3, 26.0, 21.2, 20.0, 19.4, 18.8, 15.6

Compound **23** (17,25-dihydroxy cholesterol)

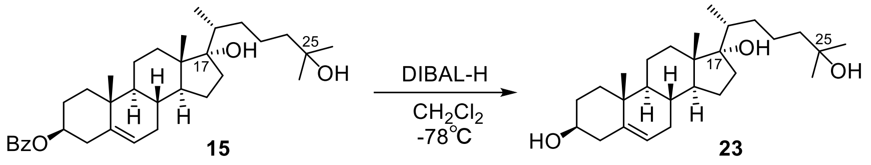

General Procedure B was followed for compound **15** (7.4 mg, 14.2 µmol). The crude mixture was purified by silica gel column chromatography (eluent: hexane/EtOAc 3/1 to 1/2) to give **22** (5.6 mg, 93%).

^{1}H NMR (500 MHz,CDCl$_3$): δ 5.35 (d, *J* = 5.4 Hz, 1H), 3.55–3.50 (m, 1H), 2.31–2.20 (m, 2H), 2.00–1.96 (m, 1H), 1.86–1.79 (m, 3H), 1.71–1.65 (m, 5H), 1.63–1.58 (m, 3H), 1.51–1.38 (m, 7H), 1.27–1.17 (m, 3H), 1.21 (s, 6H), 1.15–1.05 (m, 2H), 1.02 (s, 3H), 0.99–0.94 (m, 1H), 0.92 (d, *J* = 6.8 Hz, 3H), 0.77 (s, 3H)
^{13}C NMR (125 MHz, CDCl$_3$): δ 140.8, 121.8, 86.6, 71 1 .9, 71.2, 51.3, 49.8, 47.4, 44.3, 42.4, 39.8, 38.2, 37.4, 36.6, 32.8, 32.5, 32.4, 32.0, 31.8, 29.5, 29.4, 23.9, 22.7, 21.0, 19.5, 14.5, 14.1

Compound **24** (20,25-dihydroxy cholesterol)

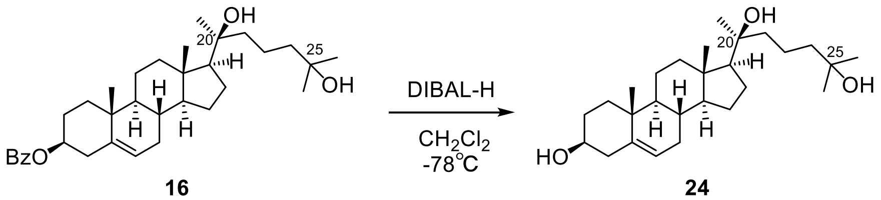

General Procedure B was followed for compound **16** (7.3 mg, 14.0 µmol). The crude mixture was purified by silica gel column chromatography (eluent: hexane/EtOAc 2/1 to 0/1) to give **24** (4.9 mg, 84%).

^{1}H NMR (500 MHz,CDCl$_3$): δ 5.35 (d, *J* = 5.4 Hz, 1H), 3.54–3.50 (m, 1H), 2.31–2.23 (m, 2H), 2.09 (d, *J* = 12.7 Hz, 1H), 1.98 (d, *J* = 17.1 Hz, 1H), 1.86–1.83 (m, 2H), 1.77–1.42 (m, 13H), 1.40–1.34 (m, 2H), 1.29 (s, 3H), 1.22–1.19 (m, 2H), 1.22 (s, 6H), 1.16–1.04 (m, 2H), 1.01 (s, 3H), 0.95–0.88 (m, 1H), 0.86 (s, 3H)
^{13}C NMR (125 MHz, CDCl$_3$): δ 140.9, 121.7, 75.4, 71.9, 71.2, 58.0, 57.0, 50.2, 44.6, 44.3, 42.8, 42.4, 40.3, 37.4, 36.7, 31.9, 31.8, 31.5, 29.7, 29.4, 26.5, 23.9, 22.6, 21.1, 19.5, 19.1, 13.8

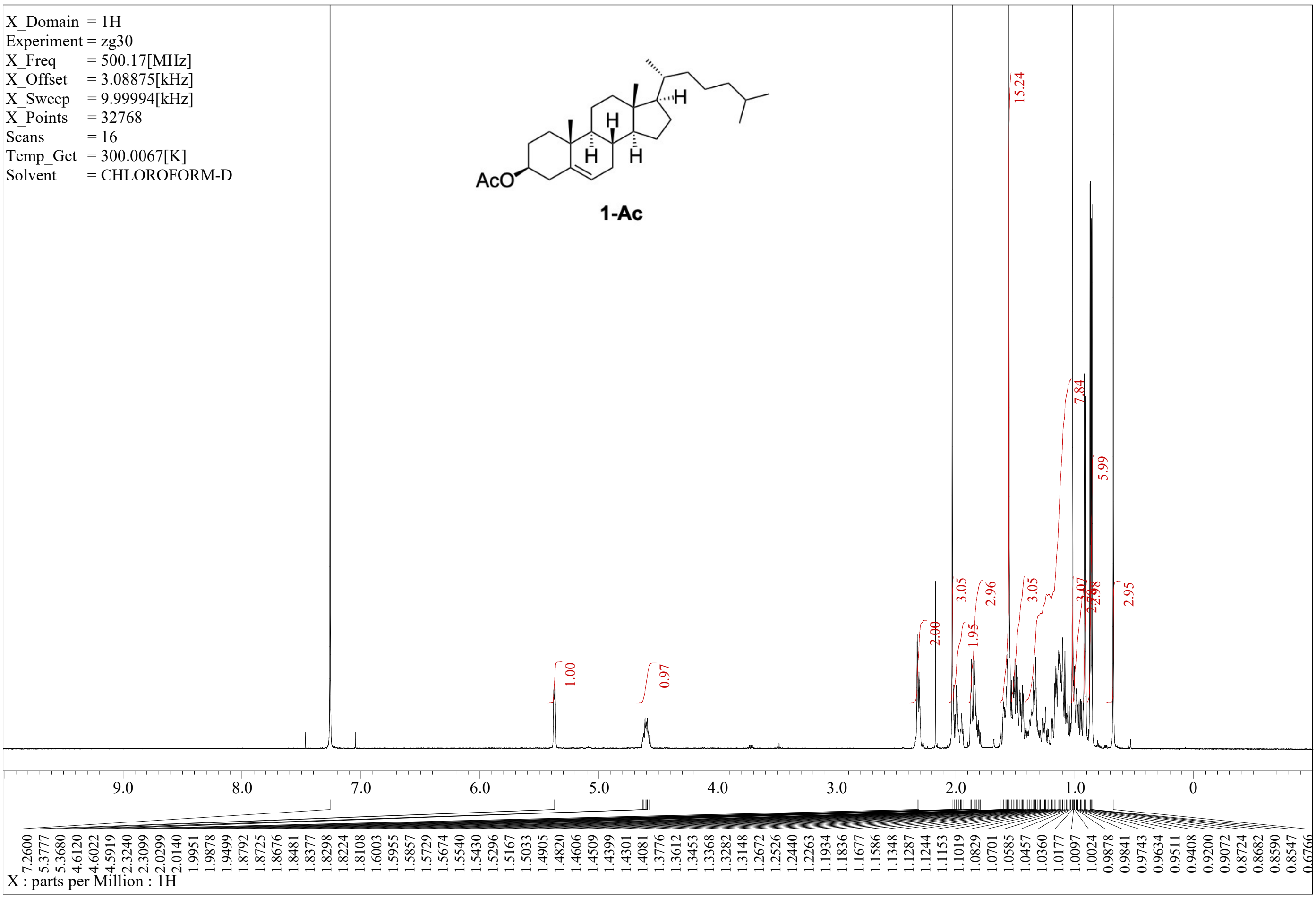
X_Domain = 1H
Experiment = zg30
X_Freq = 500.17[MHz]
X_Offset = 3.08875[kHz]
X_Sweep = 9.99994[kHz]
X_Points = 32768
Scans = 16
Temp_Get = 300.0067[K]
Solvent = CHLOROFORM-D
AcO
1-Ac
7.2600 5.3777 5.3680 4.6120 4.6022 4.5919 2.3240 2.3099 2.0299 2.0140 1.9951 1.9878 1.9499 1.8792 1.8725 1.8676 1.8481 1.8377 1.8298 1.8224 1.8108 1.6003 1.5955 1.5857 1.5729 1.5674 1.5540 1.5430 1.5296 1.5167 1.5033 1.4905 1.4820 1.4606 1.4509 1.4399 1.4301 1.4081 1.3776 1.3612 1.3453 1.3368 1.3282 1.3148 1.2672 1.2526 1.2440 1.2263 1.1934 1.1836 1.1677 1.1586 1.1348 1.1287 1.1244 1.1153 1.1019 1.0829 1.0701 1.0585 1.0457 1.0360 1.0177 1.0097 1.0024 0.9878 0.9841 0.9743 0.9634 0.9511 0.9408 0.9200 0.9072 0.8724 0.8682 0.8590 0.8547 0.6766
X : parts per Million : 1H

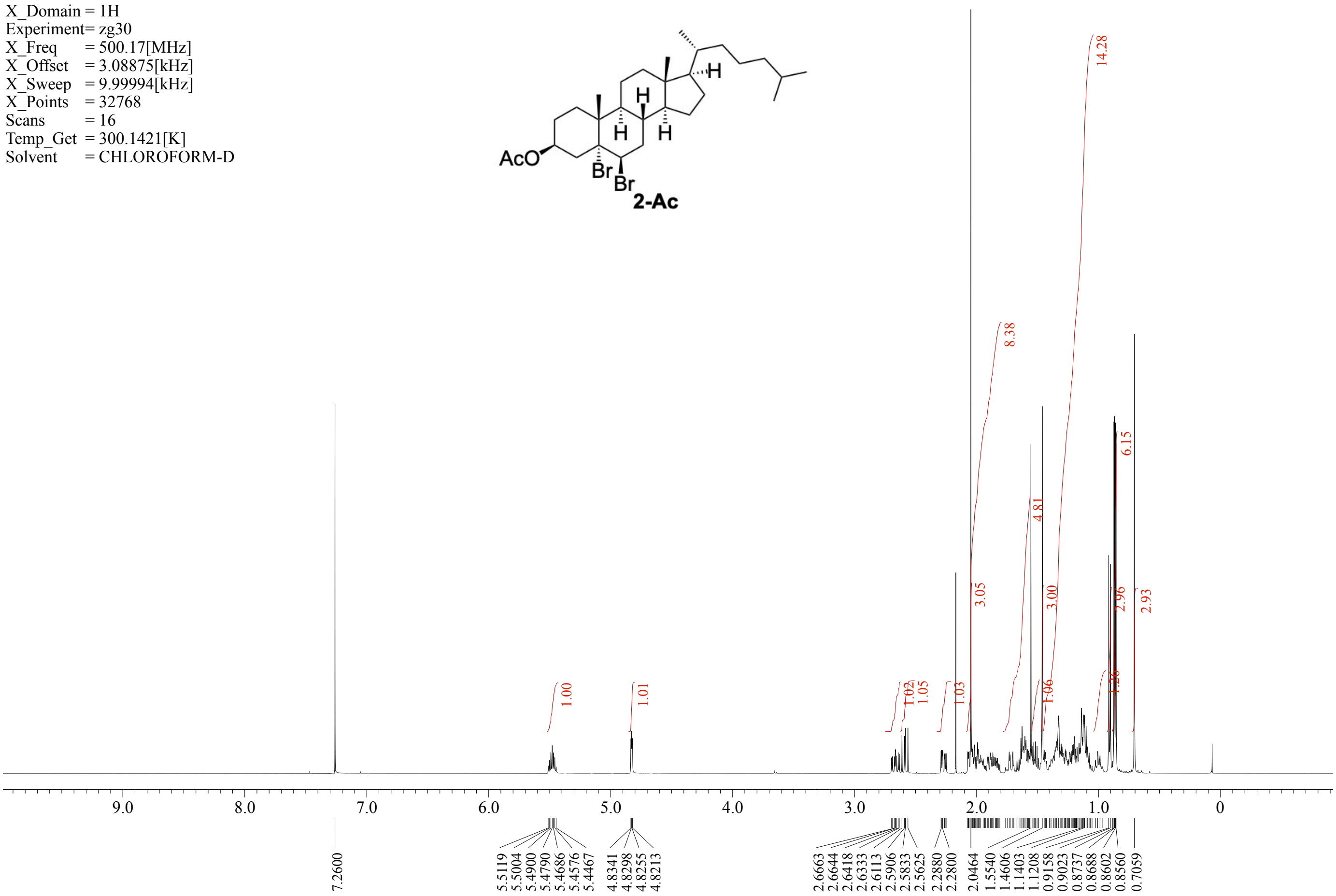
X_Domain = 1H
Experiment= zg30
X_Freq = 500.17[MHz]
X_Offset = 3.08875[kHz]
X_Sweep = 9.99994[kHz]
X_Points = 32768
Scans = 16
Temp_Get = 300.1421[K]
Solvent = CHLOROFORM-D
AcO
Br
Br
2-Ac
7.2600
5.5119 5.5004 5.4900 5.4790 5.4686 5.4576 5.4467
4.8341 4.8298 4.8255 4.8213
2.6663 2.6644 2.6418 2.6333 2.6113 2.5906 2.5833 2.5625 2.2880 2.2800
2.0464 1.5540 1.4606 1.1403 1.1208 0.9158 0.9023 0.8737 0.8688 0.8602 0.8560 0.7059

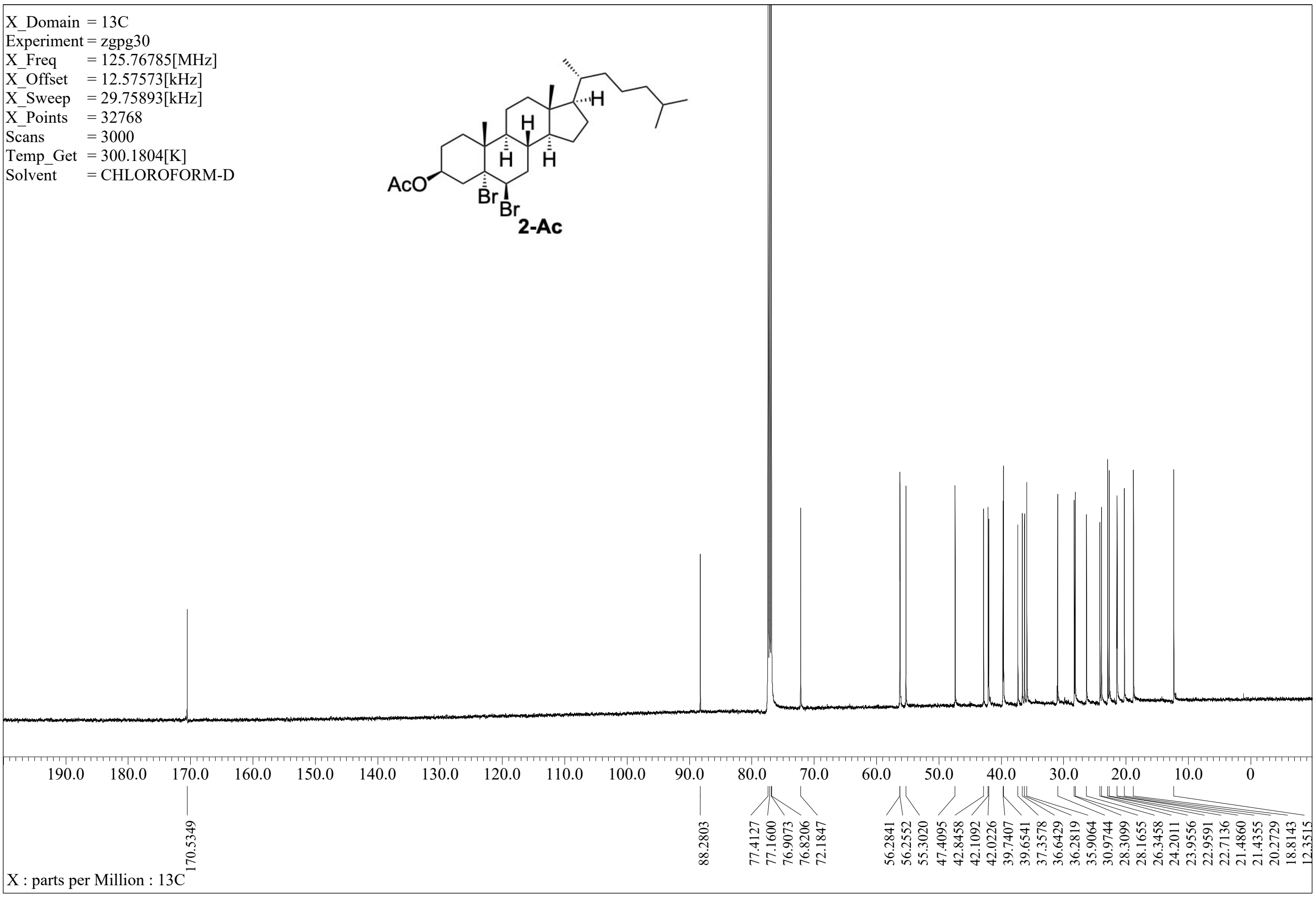
X_Domain = 13C
Experiment = zgpg30
X_Freq = 125.76785[MHz]
X_Offset = 12.57573[kHz]
X_Sweep = 29.75893[kHz]
X_Points = 32768
Scans = 3000
Temp_Get = 300.1804[K]
Solvent = CHLOROFORM-D
AcO
Br
Br
H
H
H
H
2-Ac
190.0 180.0 170.0 160.0 150.0 140.0 130.0 120.0 110.0 100.0 90.0 80.0 70.0 60.0 50.0 40.0 30.0 20.0 10.0 0
170.5349
88.2803
77.4127
77.1600
76.9073
76.8206
72.1847
56.2841
56.2552
55.3020
47.4095
42.8458
42.1092
42.0226
39.7407
39.6541
37.3578
36.6429
36.2819
35.9064
30.9744
28.3099
28.1655
26.3458
24.2011
23.9556
22.9591
22.7136
21.4860
21.4355
20.2729
18.8143
12.3515
X : parts per Million : 13C

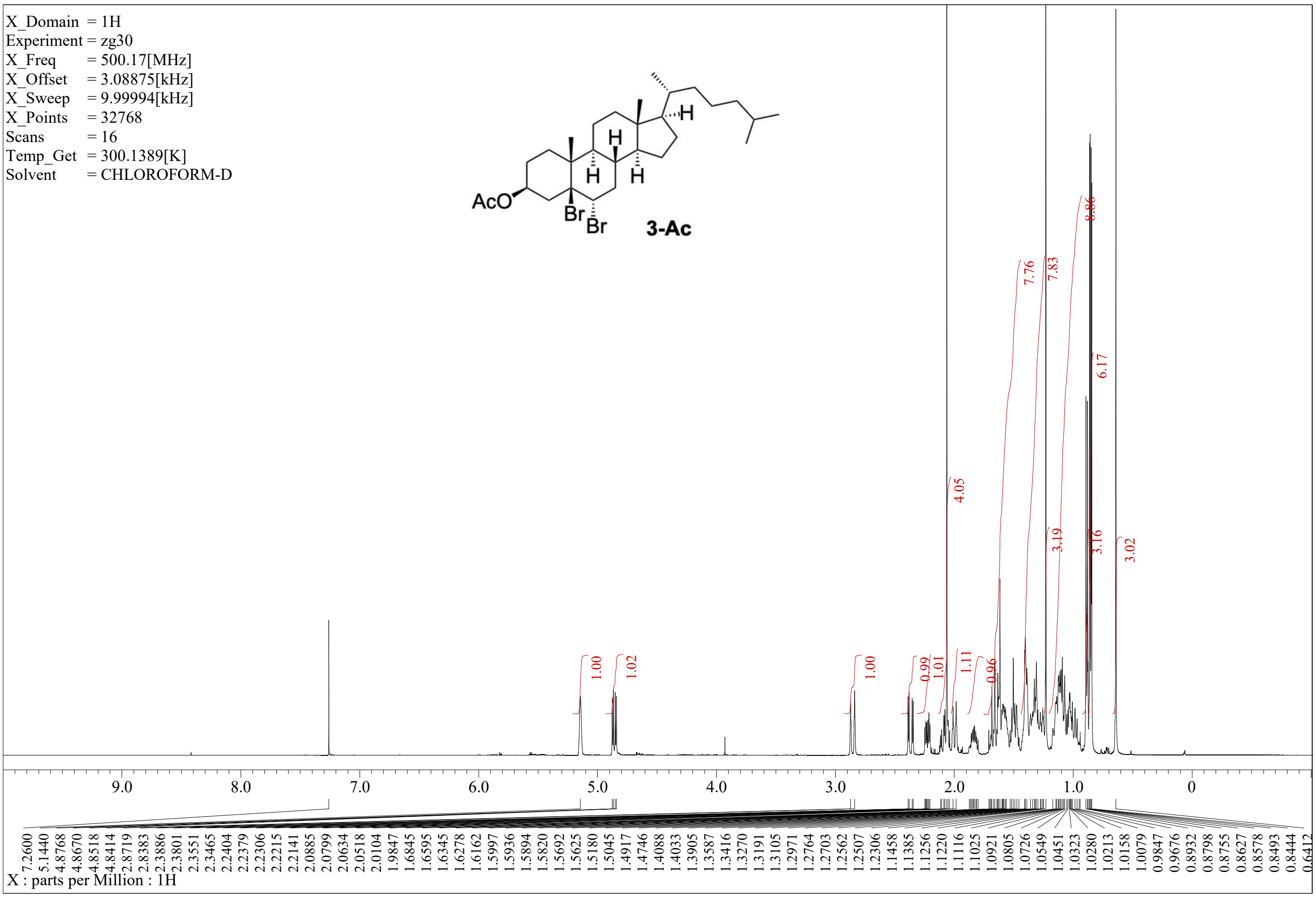
X_Domain = 1H
Experiment = zg30
X_Freq = 500.17[MHz]
X_Offset = 3.08875[kHz]
X_Sweep = 9.99994[kHz]
X_Points = 32768
Scans = 16
Temp_Get = 300.1389[K]
Solvent = CHLOROFORM-D
AcO
Br
Br
H
H
H
H
3-Ac
1.00
1.02
1.00
0.99
1.01
1.11
0.96
4.05
7.76
7.83
3.19
8.86
6.17
3.16
3.02
9.0 8.0 7.0 6.0 5.0 4.0 3.0 2.0 1.0 0
7.2600
5.1440
4.8768
4.8670
4.8518
4.8414
2.8719
2.8383
2.3886
2.3801
2.3551
2.3465
2.2404
2.2379
2.2306
2.2215
2.2141
2.0885
2.0799
2.0634
2.0518
2.0104
1.9847
1.6845
1.6595
1.6345
1.6278
1.6162
1.5997
1.5936
1.5894
1.5820
1.5692
1.5625
1.5180
1.5045
1.4917
1.4746
1.4088
1.4033
1.3905
1.3587
1.3416
1.3270
1.3191
1.3105
1.2971
1.2764
1.2703
1.2562
1.2507
1.2306
1.1458
1.1385
1.1256
1.1220
1.1116
1.1025
1.0921
1.0805
1.0726
1.0549
1.0451
1.0323
1.0280
1.0213
1.0158
1.0079
0.9847
0.9676
0.8932
0.8798
0.8755
0.8627
0.8578
0.8493
0.8444
0.6412
X : parts per Million : 1H

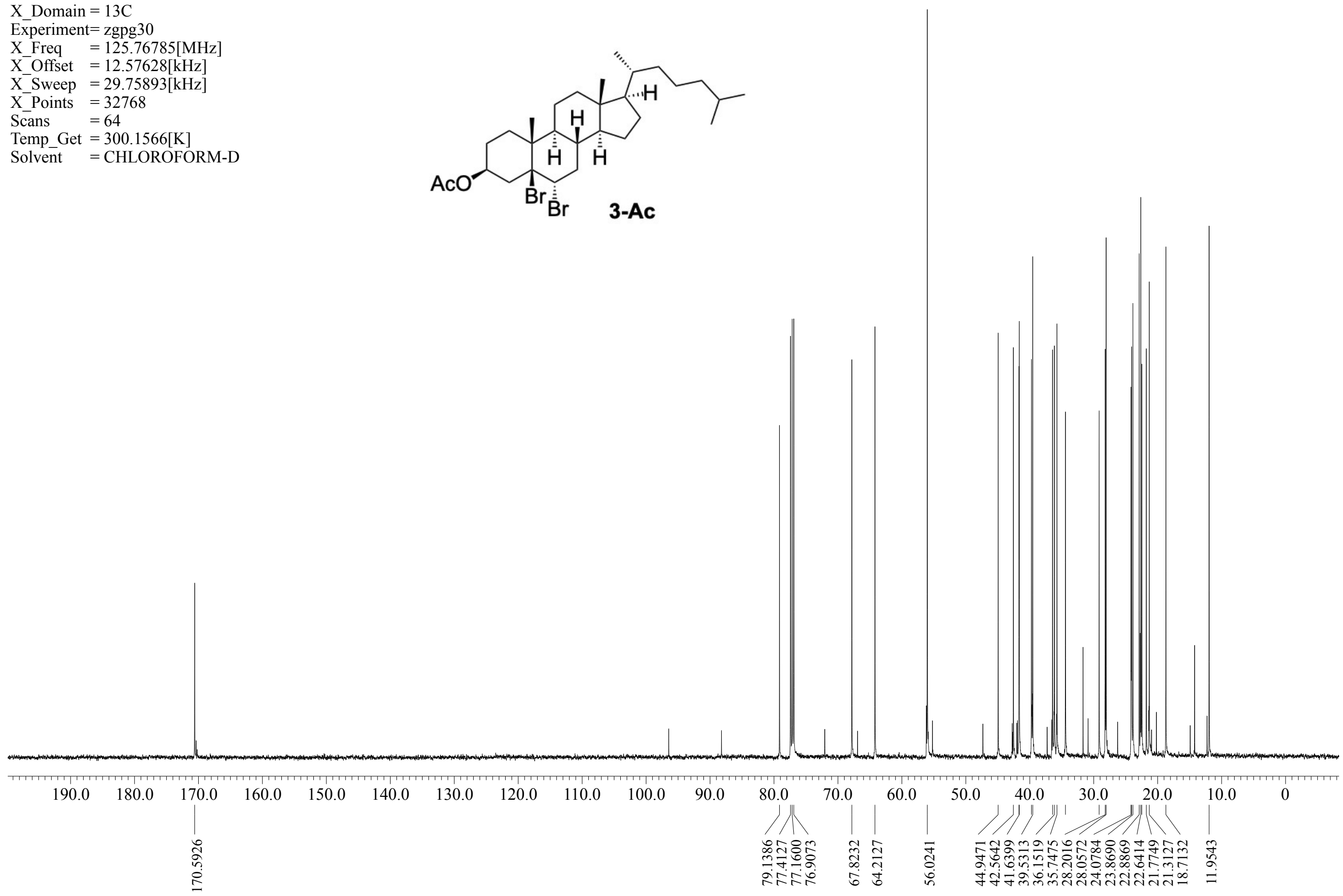
X_Domain = 13C
Experiment= zgpg30
X_Freq = 125.76785[MHz]
X_Offset = 12.57628[kHz]
X_Sweep = 29.75893[kHz]
X_Points = 32768
Scans = 64
Temp_Get = 300.1566[K]
Solvent = CHLOROFORM-D
AcO
Br
Br
H
H
H
H
3-Ac
190.0 180.0 170.0 160.0 150.0 140.0 130.0 120.0 110.0 100.0 90.0 80.0 70.0 60.0 50.0 40.0 30.0 20.0 10.0 0
170.5926
79.1386
77.4127
77.1600
76.9073
67.8232
64.2127
56.0241
44.9471
42.5642
41.6399
39.5313
36.1519
35.7475
28.2016
28.0572
24.0784
23.8690
22.8869
22.6414
21.7749
21.3127
18.7132
11.9543

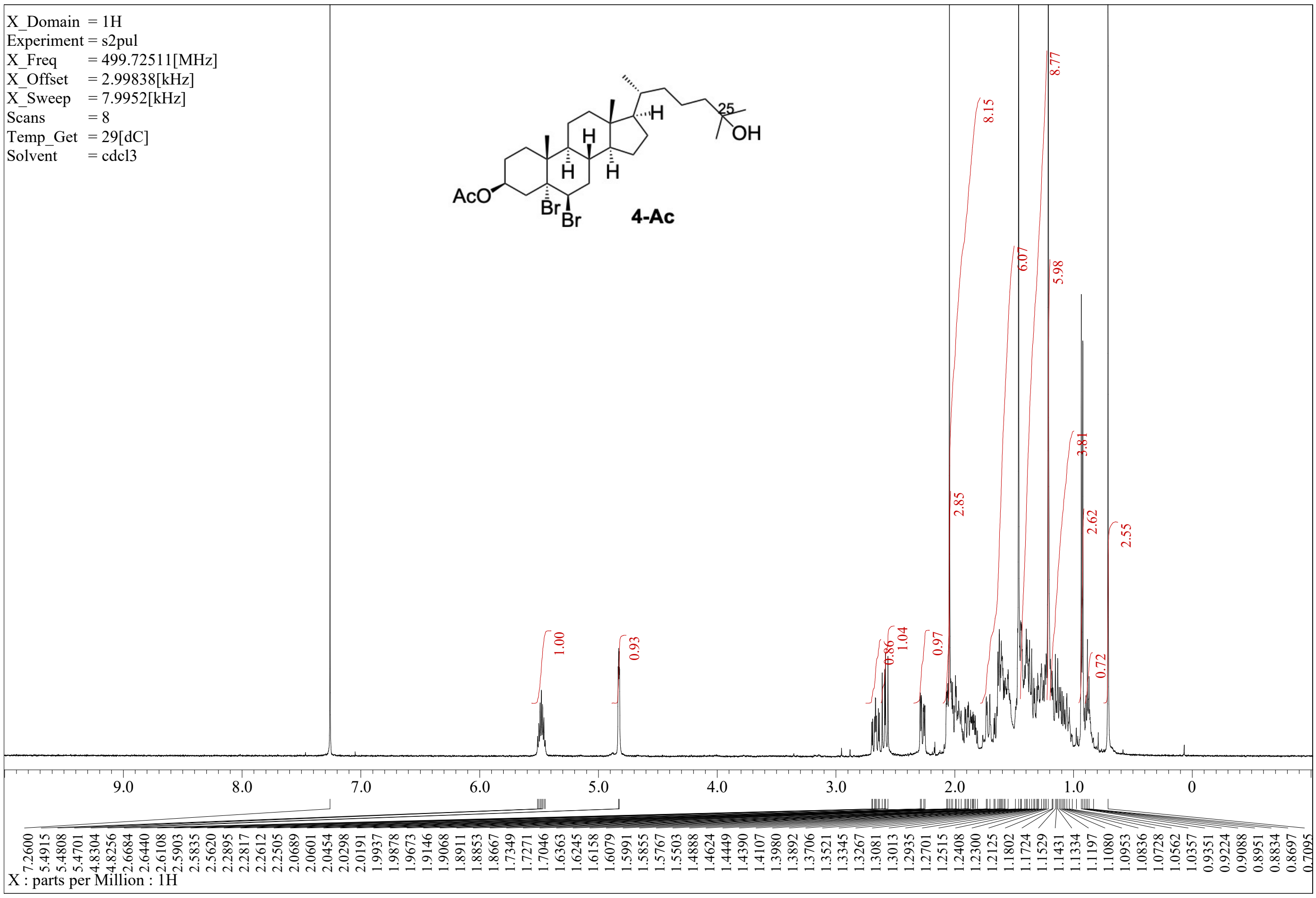
X_Domain = 1H
Experiment = s2pul
X_Freq = 499.72511[MHz]
X_Offset = 2.99838[kHz]
X_Sweep = 7.9952[kHz]
Scans = 8
Temp_Get = 29[dC]
Solvent = cdcl3
AcO
Br
Br
H
H
H
H
25
OH
4-Ac
8.77
8.15
6.07
5.98
3.81
2.85
2.62
2.55
1.00
0.93
0.86
1.04
0.97
0.72
9.0 8.0 7.0 6.0 5.0 4.0 3.0 2.0 1.0 0
7.2600
5.4915
5.4808
5.4701
4.8304
4.8256
2.6684
2.6440
2.6108
2.5903
2.5835
2.5620
2.2895
2.2817
2.2612
2.2505
2.0689
2.0601
2.0454
2.0298
2.0191
1.9937
1.9878
1.9673
1.9146
1.9068
1.8911
1.8853
1.8667
1.7349
1.7271
1.7046
1.6363
1.6245
1.6158
1.6079
1.5991
1.5855
1.5767
1.5503
1.4888
1.4624
1.4449
1.4390
1.4107
1.3980
1.3892
1.3706
1.3521
1.3345
1.3267
1.3081
1.3013
1.2935
1.2701
1.2515
1.2408
1.2300
1.2125
1.1802
1.1724
1.1529
1.1431
1.1334
1.1197
1.1080
1.0953
1.0836
1.0728
1.0562
1.0357
0.9351
0.9224
0.9088
0.8951
0.8834
0.8697
0.7095
X : parts per Million : 1H

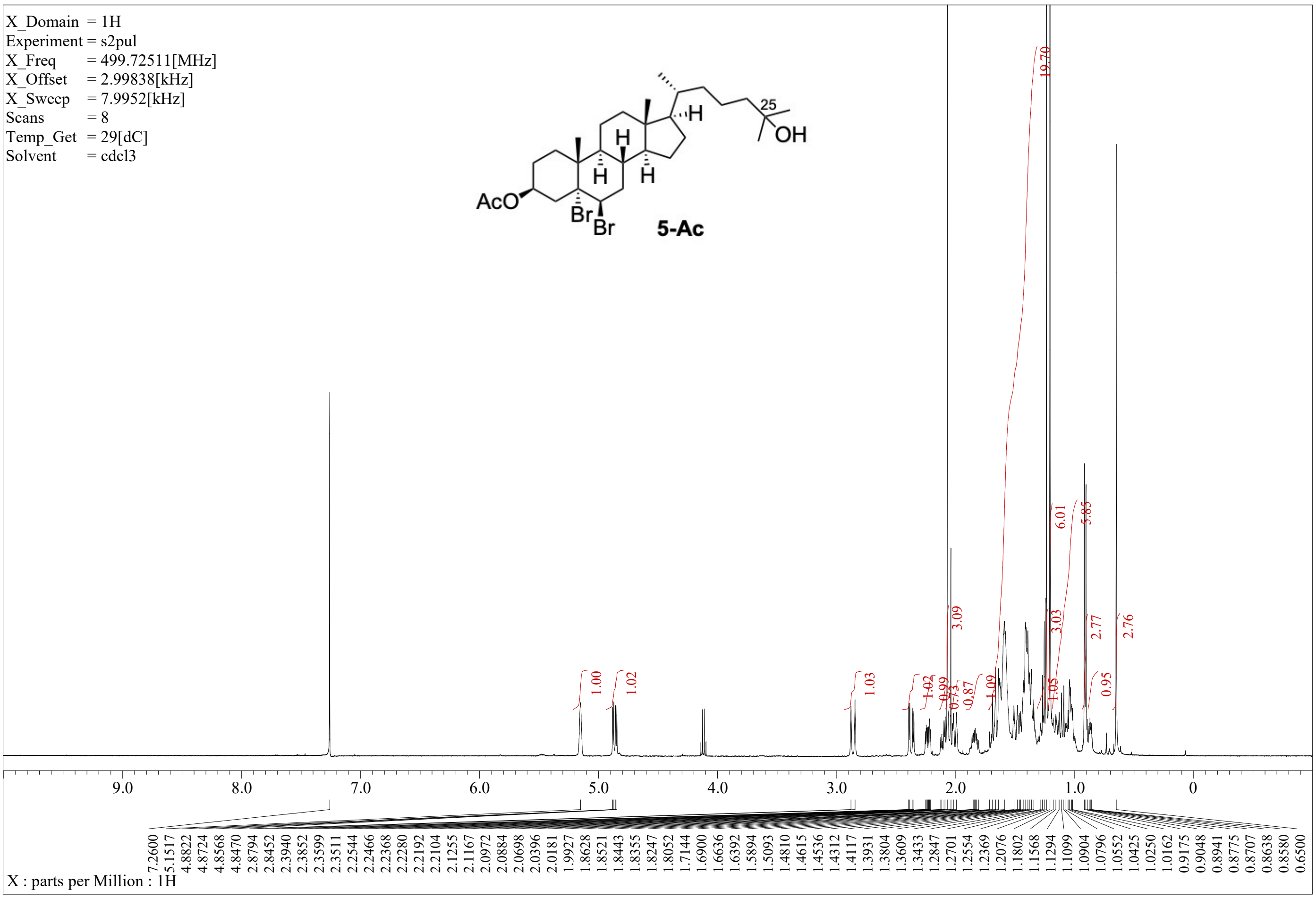
X_Domain = 1H
Experiment = s2pul
X_Freq = 499.72511[MHz]
X_Offset = 2.99838[kHz]
X_Sweep = 7.9952[kHz]
Scans = 8
Temp_Get = 29[dC]
Solvent = cdcl3
AcO
Br
Br
H
H
H
H
25
OH
5-Ac
9.0 8.0 7.0 6.0 5.0 4.0 3.0 2.0 1.0 0
7.2600 5.1517 4.8822 4.8724 4.8568 4.8470 2.8794 2.8452 2.3940 2.3852 2.3599 2.3511 2.2544 2.2466 2.2368 2.2280 2.2192 2.2104 2.1255 2.1167 2.0972 2.0884 2.0698 2.0396 2.0181 1.9927 1.8628 1.8521 1.8443 1.8355 1.8247 1.8052 1.7144 1.6900 1.6636 1.6392 1.5894 1.5093 1.4810 1.4615 1.4536 1.4312 1.4117 1.3931 1.3804 1.3609 1.3433 1.2847 1.2701 1.2554 1.2369 1.2076 1.1802 1.1568 1.1294 1.1099 1.0904 1.0796 1.0552 1.0425 1.0250 1.0162 0.9175 0.9048 0.8941 0.8775 0.8707 0.8638 0.8580 0.6500
X : parts per Million : 1H

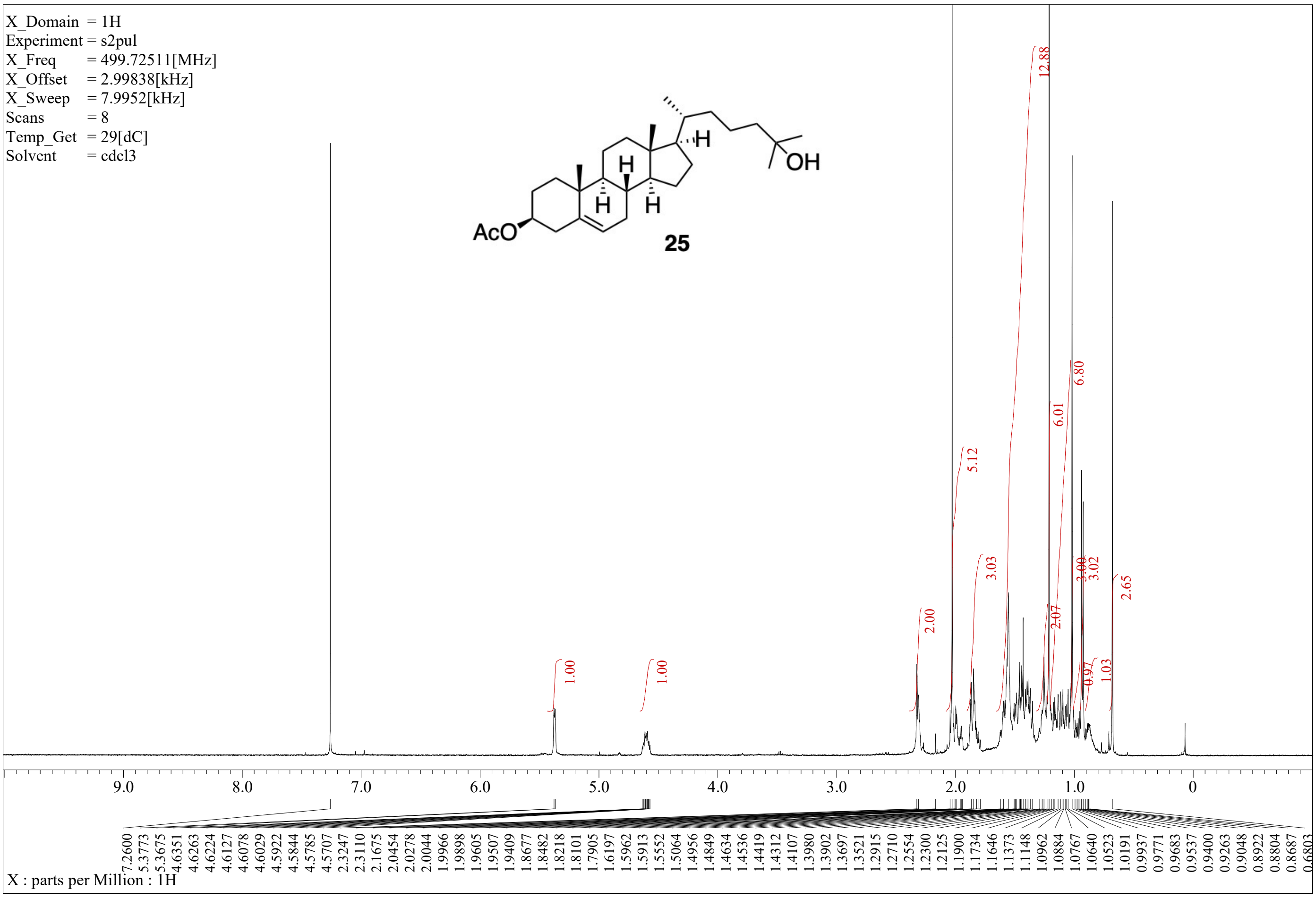
X_Domain = 1H
Experiment = s2pul
X_Freq = 499.72511[MHz]
X_Offset = 2.99838[kHz]
X_Sweep = 7.9952[kHz]
Scans = 8
Temp_Get = 29[dC]
Solvent = cdcl3
AcO
H
H
H
H
OH
25
9.0 8.0 7.0 6.0 5.0 4.0 3.0 2.0 1.0 0
7.2600 5.3773 5.3675 4.6351 4.6263 4.6224 4.6127 4.6078 4.6029 4.5922 4.5844 4.5785 4.5707 2.3247 2.3110 2.1675 2.0454 2.0278 2.0044 1.9966 1.9898 1.9605 1.9507 1.9409 1.8677 1.8482 1.8218 1.8101 1.7905 1.6197 1.5962 1.5913 1.5552 1.5064 1.4956 1.4849 1.4634 1.4536 1.4419 1.4312 1.4107 1.3980 1.3902 1.3697 1.3521 1.2915 1.2710 1.2554 1.2300 1.2125 1.1900 1.1734 1.1646 1.1373 1.1148 1.0962 1.0884 1.0767 1.0640 1.0523 1.0191 0.9937 0.9771 0.9683 0.9537 0.9400 0.9263 0.9048 0.8922 0.8804 0.8687 0.6803
X : parts per Million : 1H

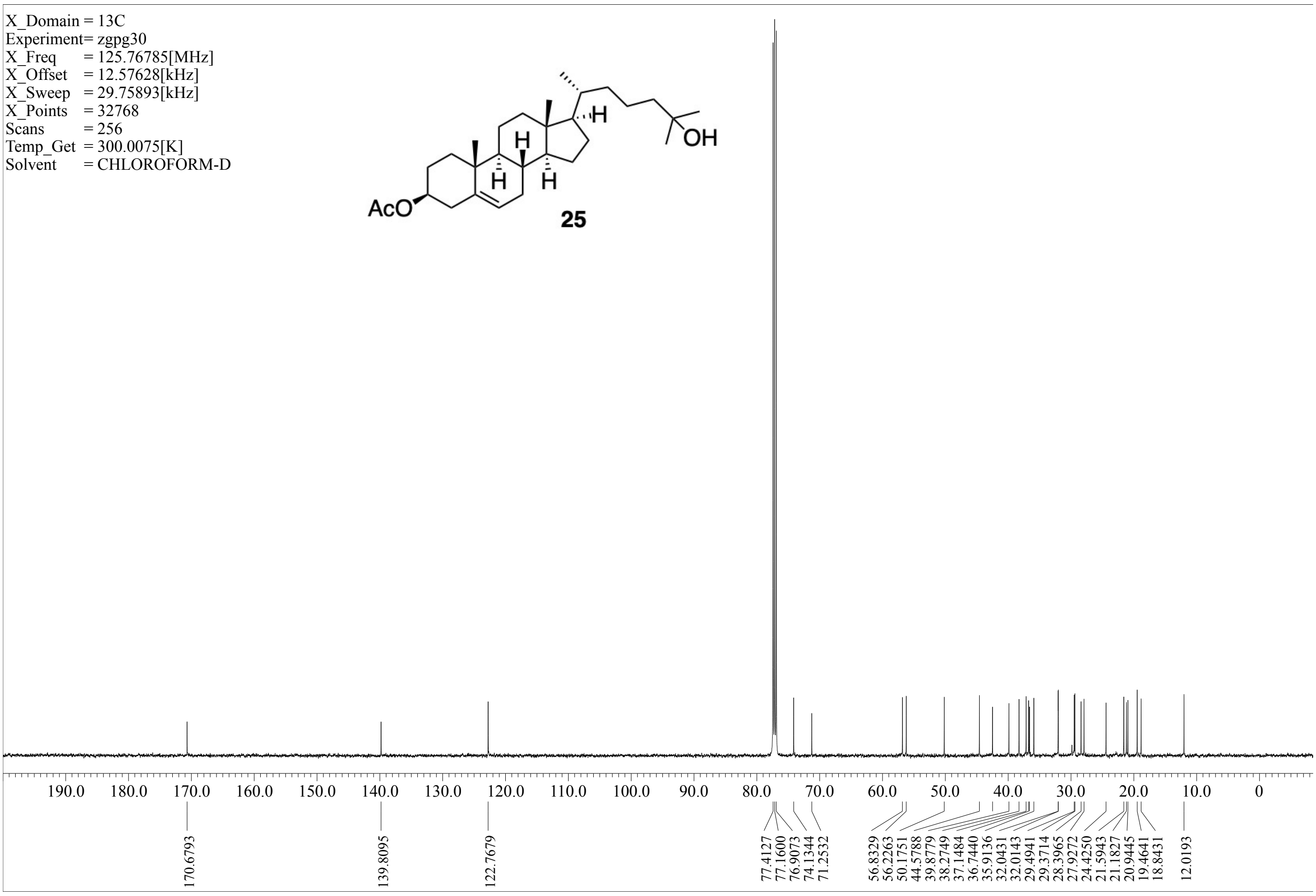
X_Domain = 13C
Experiment= zgpg30
X_Freq = 125.76785[MHz]
X_Offset = 12.57628[kHz]
X_Sweep = 29.75893[kHz]
X_Points = 32768
Scans = 256
Temp_Get = 300.0075[K]
Solvent = CHLOROFORM-D
AcO
H
OH
25
190.0 180.0 170.0 160.0 150.0 140.0 130.0 120.0 110.0 100.0 90.0 80.0 70.0 60.0 50.0 40.0 30.0 20.0 10.0 0
170.6793
139.8095
122.7679
77.4127
77.1600
76.9073
74.1344
71.2532
56.8329
56.2263
50.1751
44.5788
39.8779
38.2749
37.1484
36.7440
35.9136
32.0431
32.0143
29.4941
29.3714
28.3965
27.9272
24.4250
21.5943
21.1827
20.9445
19.4641
18.8431
12.0193

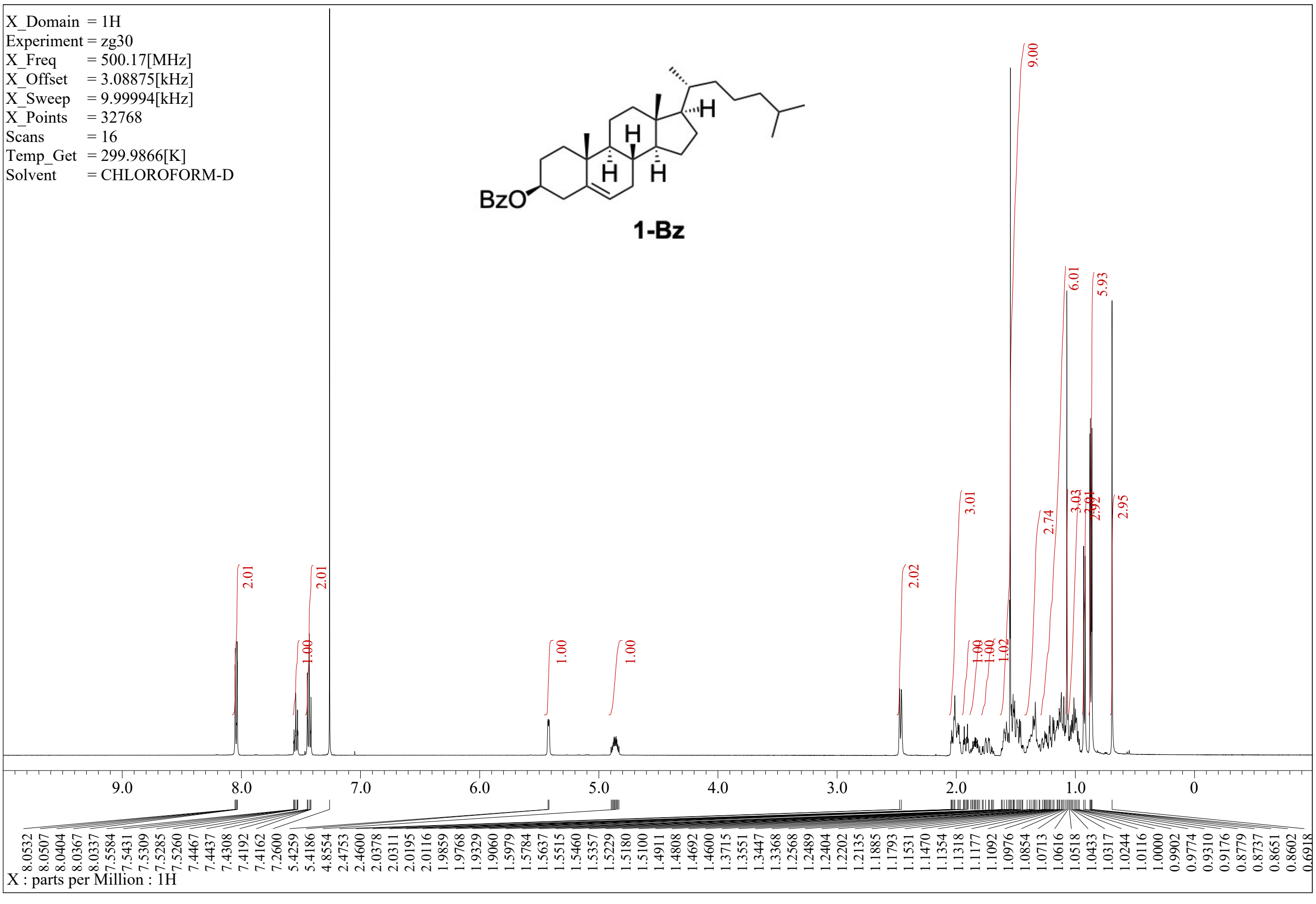
X_Domain = 1H
Experiment = zg30
X_Freq = 500.17[MHz]
X_Offset = 3.08875[kHz]
X_Sweep = 9.99994[kHz]
X_Points = 32768
Scans = 16
Temp_Get = 299.9866[K]
Solvent = CHLOROFORM-D
BzO
H
1-Bz
2.01
1.00
2.01
1.00
1.00
2.02
3.01
1.00
1.00
1.02
9.00
2.74
6.01
3.03
3.01
2.92
5.93
2.95
9.0 8.0 7.0 6.0 5.0 4.0 3.0 2.0 1.0 0
8.0532
8.0507
8.0404
8.0367
8.0337
7.5584
7.5431
7.5309
7.5285
7.5260
7.4467
7.4437
7.4308
7.4192
7.4162
7.2600
5.4259
5.4186
4.8554
2.4753
2.4600
2.0378
2.0311
2.0195
2.0116
1.9859
1.9768
1.9329
1.9060
1.5979
1.5784
1.5637
1.5515
1.5460
1.5357
1.5229
1.5180
1.5100
1.4911
1.4808
1.4692
1.4600
1.3715
1.3551
1.3447
1.3368
1.2568
1.2489
1.2404
1.2202
1.2135
1.1885
1.1793
1.1531
1.1470
1.1354
1.1318
1.1177
1.1092
1.0976
1.0854
1.0713
1.0616
1.0518
1.0433
1.0317
1.0244
1.0116
1.0000
0.9902
0.9774
0.9310
0.9176
0.8779
0.8737
0.8651
0.8602
0.6918
X : parts per Million : 1H

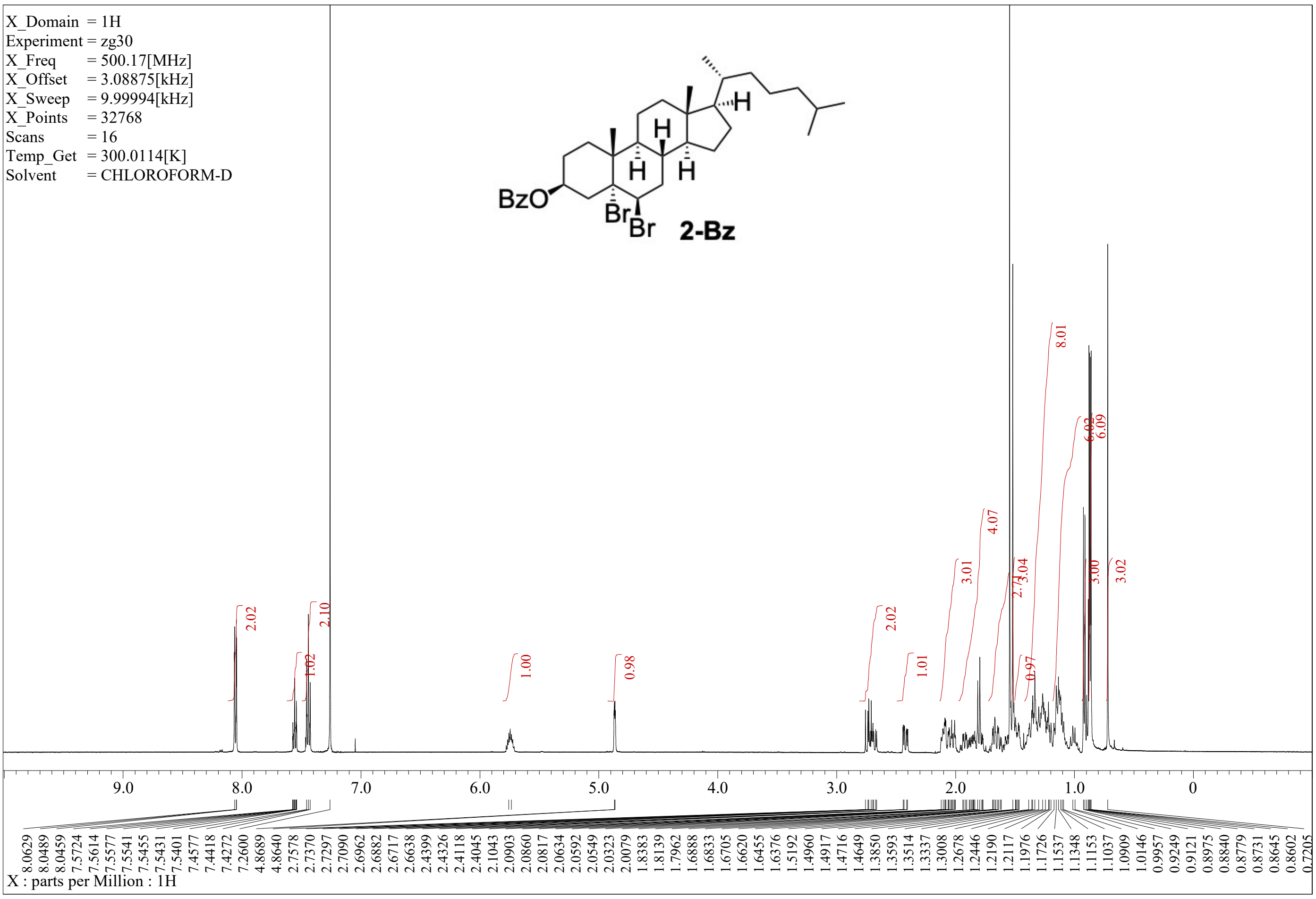
X_Domain = 1H
Experiment = zg30
X_Freq = 500.17[MHz]
X_Offset = 3.08875[kHz]
X_Sweep = 9.99994[kHz]
X_Points = 32768
Scans = 16
Temp_Get = 300.0114[K]
Solvent = CHLOROFORM-D
BzO
Br
Br
H
H
H
H
2-Bz
2.02
1.02
2.10
1.00
0.98
2.02
1.01
3.01
4.07
3.04
0.97
8.01
6.02
6.09
3.00
3.02
9.0
8.0
7.0
6.0
5.0
4.0
3.0
2.0
1.0
0
8.0629 8.0489 8.0459 7.5724 7.5614 7.5577 7.5541 7.5455 7.5431 7.5401 7.4577 7.4418 7.4272 7.2600 4.8689 4.8640 2.7578 2.7370 2.7297 2.7090 2.6962 2.6882 2.6717 2.6638 2.4399 2.4326 2.4118 2.4045 2.1043 2.0903 2.0860 2.0817 2.0634 2.0592 2.0549 2.0323 2.0079 1.8383 1.8139 1.7962 1.6888 1.6833 1.6705 1.6620 1.6455 1.6376 1.5192 1.4960 1.4917 1.4716 1.4649 1.3850 1.3593 1.3514 1.3337 1.3008 1.2678 1.2446 1.2190 1.2117 1.1976 1.1726 1.1537 1.1348 1.1153 1.1103 1.0909 1.0146 0.9957 0.9249 0.9121 0.8975 0.8840 0.8779 0.8731 0.8645 0.8602 0.7205
X : parts per Million : 1H

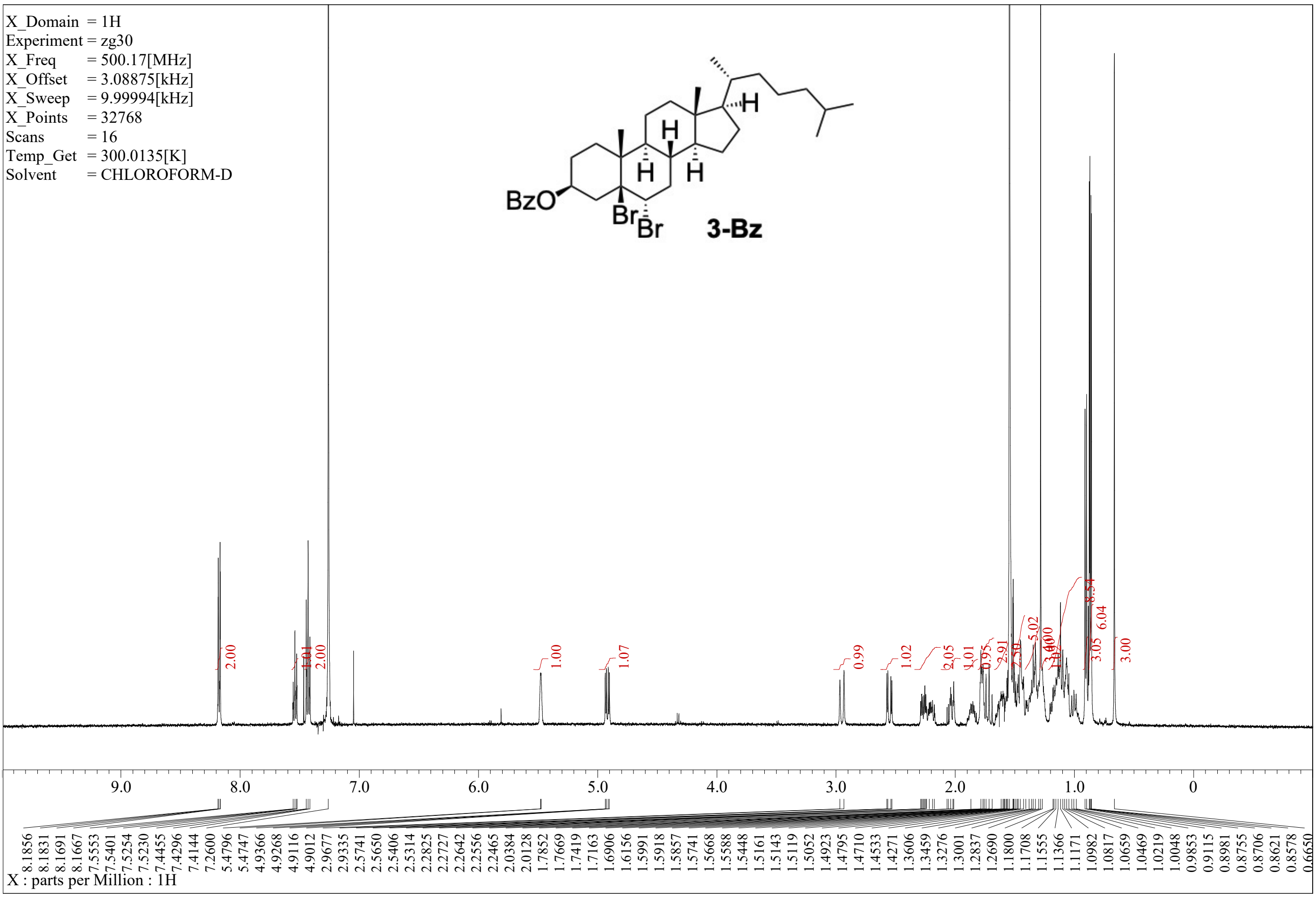
X_Domain = 1H
Experiment = zg30
X_Freq = 500.17[MHz]
X_Offset = 3.08875[kHz]
X_Sweep = 9.99994[kHz]
X_Points = 32768
Scans = 16
Temp_Get = 300.0135[K]
Solvent = CHLOROFORM-D
BzO
Br
Br
H
H
H
H
3-Bz
2.00
1.01
2.00
1.00
1.07
0.99
1.02
2.05
1.01
0.95
2.91
5.02
6.04
3.05
3.00
9.0
8.0
7.0
6.0
5.0
4.0
3.0
2.0
1.0
0
8.1856 8.1831 8.1691 8.1667 7.5553 7.5401 7.5254 7.5230 7.4455 7.4296 7.4144 7.2600 5.4796 5.4747 4.9366 4.9268 4.9116 4.9012 2.9677 2.9335 2.5741 2.5650 2.5406 2.5314 2.2825 2.2727 2.2642 2.2556 2.2465 2.0384 2.0128 1.7852 1.7669 1.7419 1.7163 1.6906 1.6156 1.5991 1.5918 1.5857 1.5741 1.5668 1.5588 1.5448 1.5161 1.5143 1.5119 1.5052 1.4923 1.4795 1.4710 1.4533 1.4271 1.3606 1.3459 1.3276 1.3001 1.2837 1.2690 1.1800 1.1708 1.1555 1.1366 1.1171 1.0982 1.0817 1.0659 1.0469 1.0219 1.0048 0.9853 0.9115 0.8981 0.8755 0.8706 0.8621 0.8578 0.6650
X : parts per Million : 1H

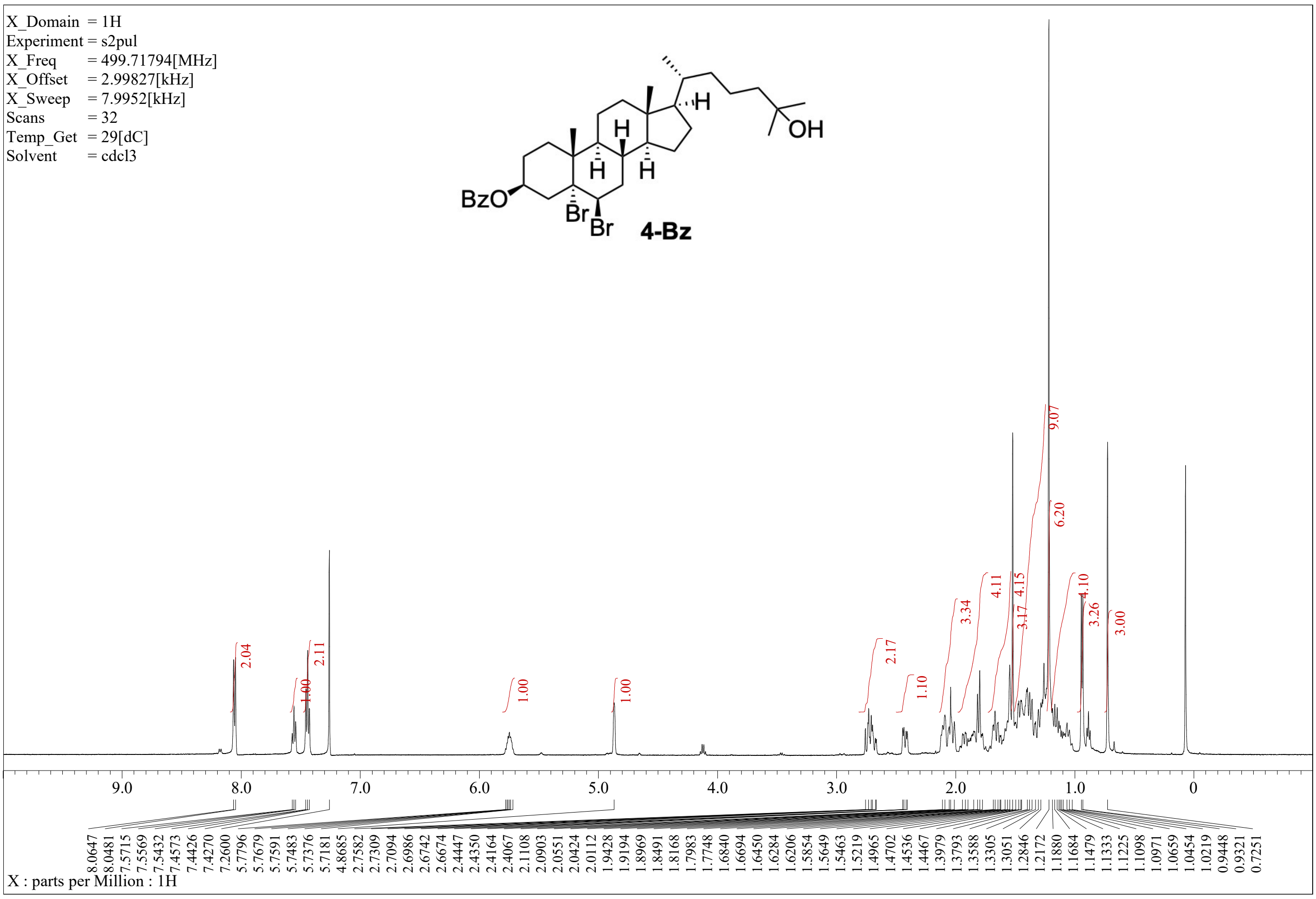
X_Domain = 1H
Experiment = s2pul
X_Freq = 499.71794[MHz]
X_Offset = 2.99827[kHz]
X_Sweep = 7.9952[kHz]
Scans = 32
Temp_Get = 29[dC]
Solvent = cdcl3
BzO
Br
Br
H
H
H
H
OH
4-Bz
2.04
1.00
2.11
1.00
1.00
2.17
1.10
3.34
4.11
3.17
4.15
9.07
6.20
4.10
3.26
3.00
8.0647
8.0481
7.5715
7.5569
7.5432
7.4573
7.4426
7.4270
7.2600
5.7796
5.7679
5.7591
5.7483
5.7376
5.7181
4.8685
2.7582
2.7309
2.7094
2.6986
2.6742
2.6674
2.4447
2.4350
2.4164
2.4067
2.1108
2.0903
2.0551
2.0424
2.0112
1.9428
1.9194
1.8969
1.8491
1.8168
1.7983
1.7748
1.6840
1.6694
1.6450
1.6284
1.6206
1.5854
1.5649
1.5463
1.5219
1.4965
1.4702
1.4536
1.4467
1.3979
1.3793
1.3588
1.3305
1.3051
1.2846
1.2172
1.1880
1.1684
1.1479
1.1333
1.1225
1.1098
1.0971
1.0659
1.0454
1.0219
0.9448
0.9321
0.7251
X : parts per Million : 1H

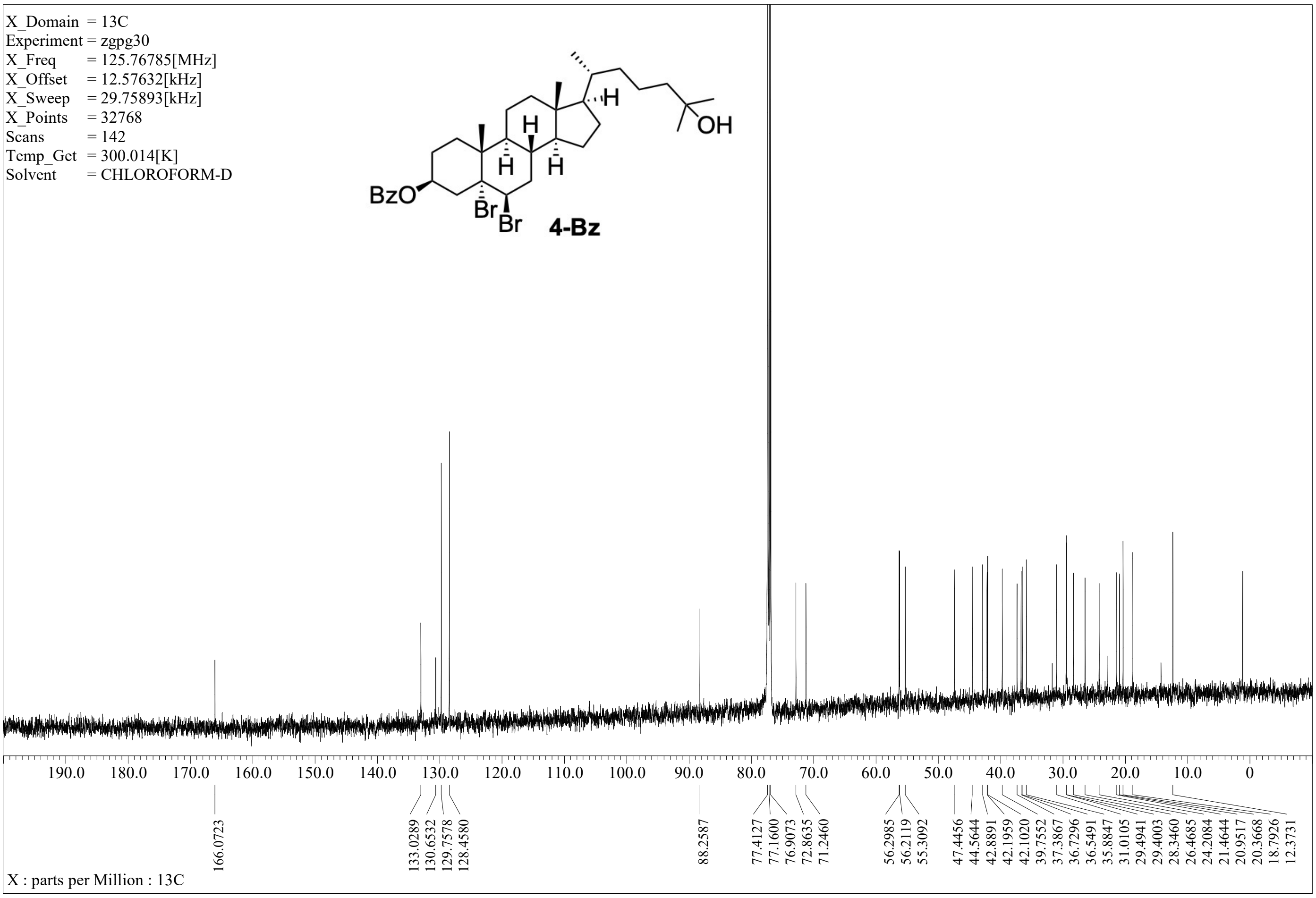
X_Domain = 13C
Experiment = zgpg30
X_Freq = 125.76785[MHz]
X_Offset = 12.57632[kHz]
X_Sweep = 29.75893[kHz]
X_Points = 32768
Scans = 142
Temp_Get = 300.014[K]
Solvent = CHLOROFORM-D
BzO
Br
Br
H
H
H
H
OH
4-Bz
166.0723
133.0289
130.6532
129.7578
128.4580
88.2587
77.4127
77.1600
76.9073
72.8635
71.2460
56.2985
56.2119
55.3092
47.4456
44.5644
42.8891
42.1959
42.1020
39.7552
37.3867
36.7296
36.5491
35.8847
31.0105
29.4941
29.4003
28.3460
26.4685
24.2084
21.4644
20.9517
20.3668
18.7926
12.3731
X : parts per Million : 13C

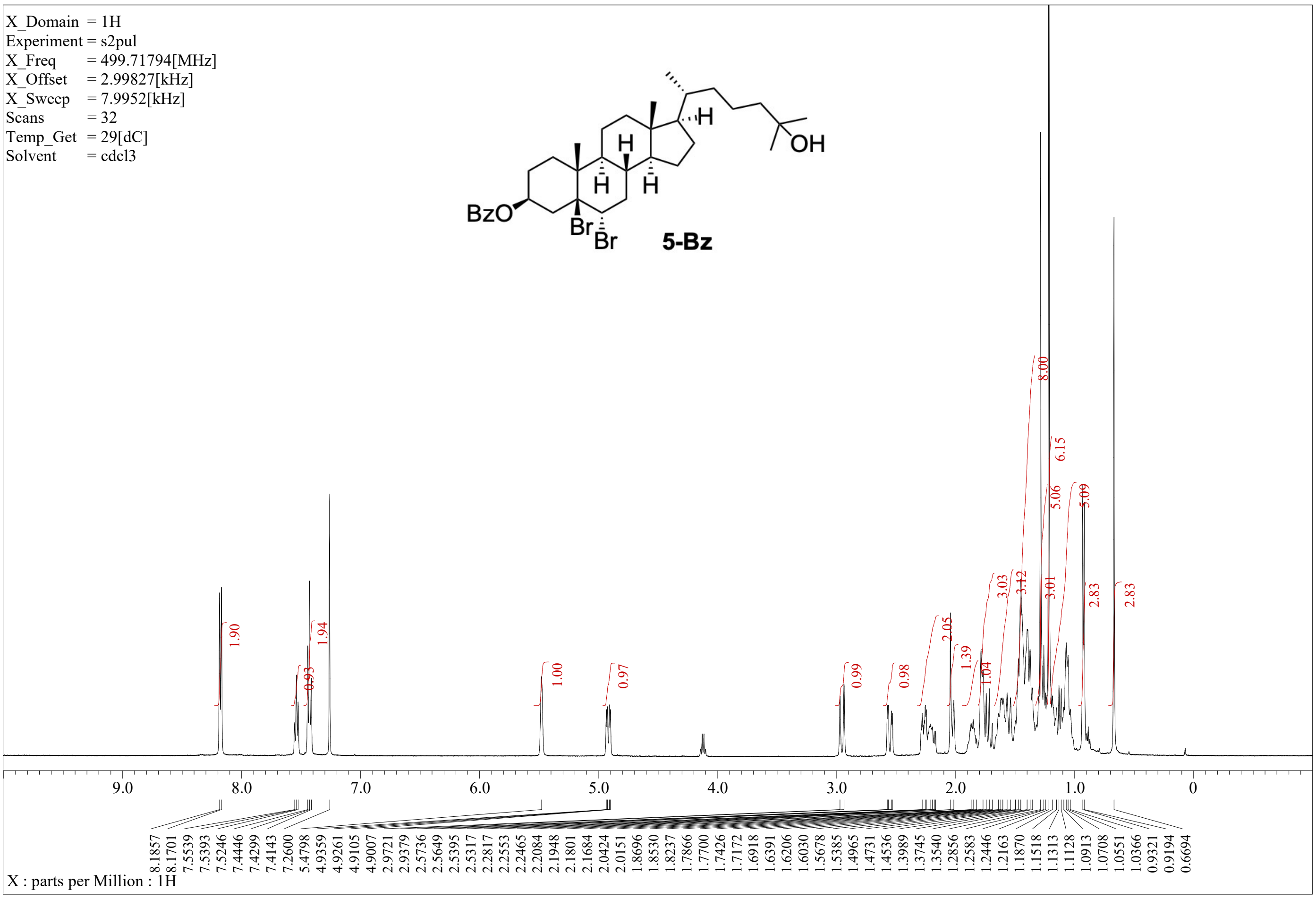
X_Domain = 1H
Experiment = s2pul
X_Freq = 499.71794[MHz]
X_Offset = 2.99827[kHz]
X_Sweep = 7.9952[kHz]
Scans = 32
Temp_Get = 29[dC]
Solvent = cdcl3
BzO
Br
Br
H
H
H
H
OH
5-Bz
1.90
0.93
1.94
1.00
0.97
0.99
0.98
2.05
1.39
1.04
3.03
3.12
8.00
3.01
5.06
6.15
5.09
2.83
2.83
9.0
8.0
7.0
6.0
5.0
4.0
3.0
2.0
1.0
0
8.1857
8.1701
7.5539
7.5393
7.5246
7.4446
7.4299
7.4143
7.2600
5.4798
4.9359
4.9261
4.9105
4.9007
2.9721
2.9379
2.5736
2.5649
2.5395
2.5317
2.2817
2.2553
2.2465
2.2084
2.1948
2.1801
2.1684
2.0424
2.0151
1.8696
1.8530
1.8237
1.7866
1.7700
1.7426
1.7172
1.6918
1.6391
1.6206
1.6030
1.5678
1.5385
1.4965
1.4731
1.4536
1.3989
1.3745
1.3540
1.2856
1.2583
1.2446
1.2163
1.1870
1.1518
1.1313
1.1128
1.0913
1.0708
1.0551
1.0366
0.9321
0.9194
0.6694
X : parts per Million : 1H

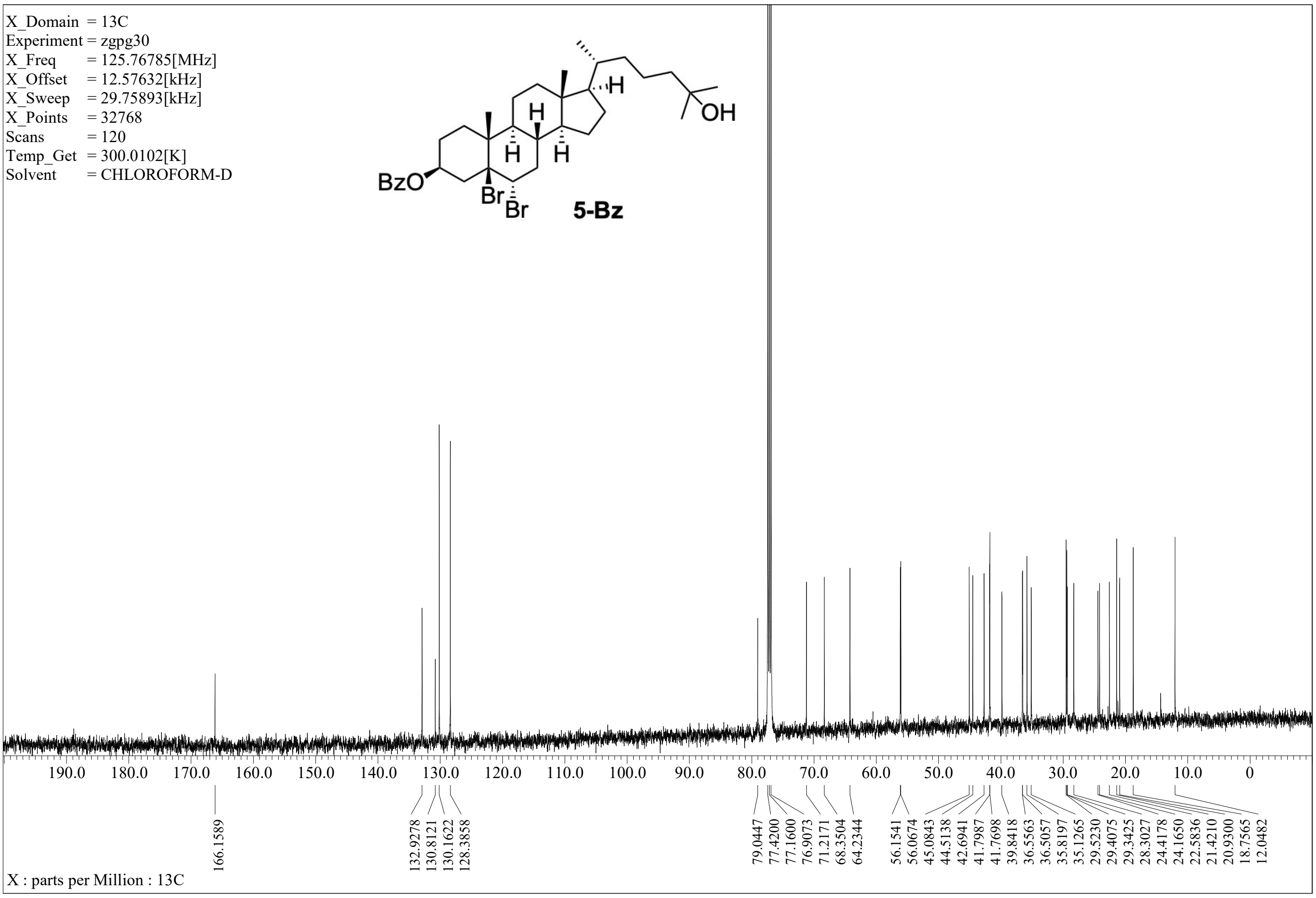
X_Domain = 13C
Experiment = zgpg30
X_Freq = 125.76785[MHz]
X_Offset = 12.57632[kHz]
X_Sweep = 29.75893[kHz]
X_Points = 32768
Scans = 120
Temp_Get = 300.0102[K]
Solvent = CHLOROFORM-D
BzO
Br
Br
H
H
H
H
OH
5-Bz
190.0
180.0
170.0
160.0
150.0
140.0
130.0
120.0
110.0
100.0
90.0
80.0
70.0
60.0
50.0
40.0
30.0
20.0
10.0
0
166.1589
132.9278
130.8121
130.1622
128.3858
79.0447
77.4200
77.1600
76.9073
71.2171
68.3504
64.2344
56.1541
56.0674
45.0843
44.5138
42.6941
41.7987
41.7698
39.8418
36.5563
36.5057
35.8197
35.1265
29.5230
29.4075
29.3425
28.3027
24.4178
24.1650
22.5836
21.4210
20.9300
18.7565
12.0482
X : parts per Million : 13C

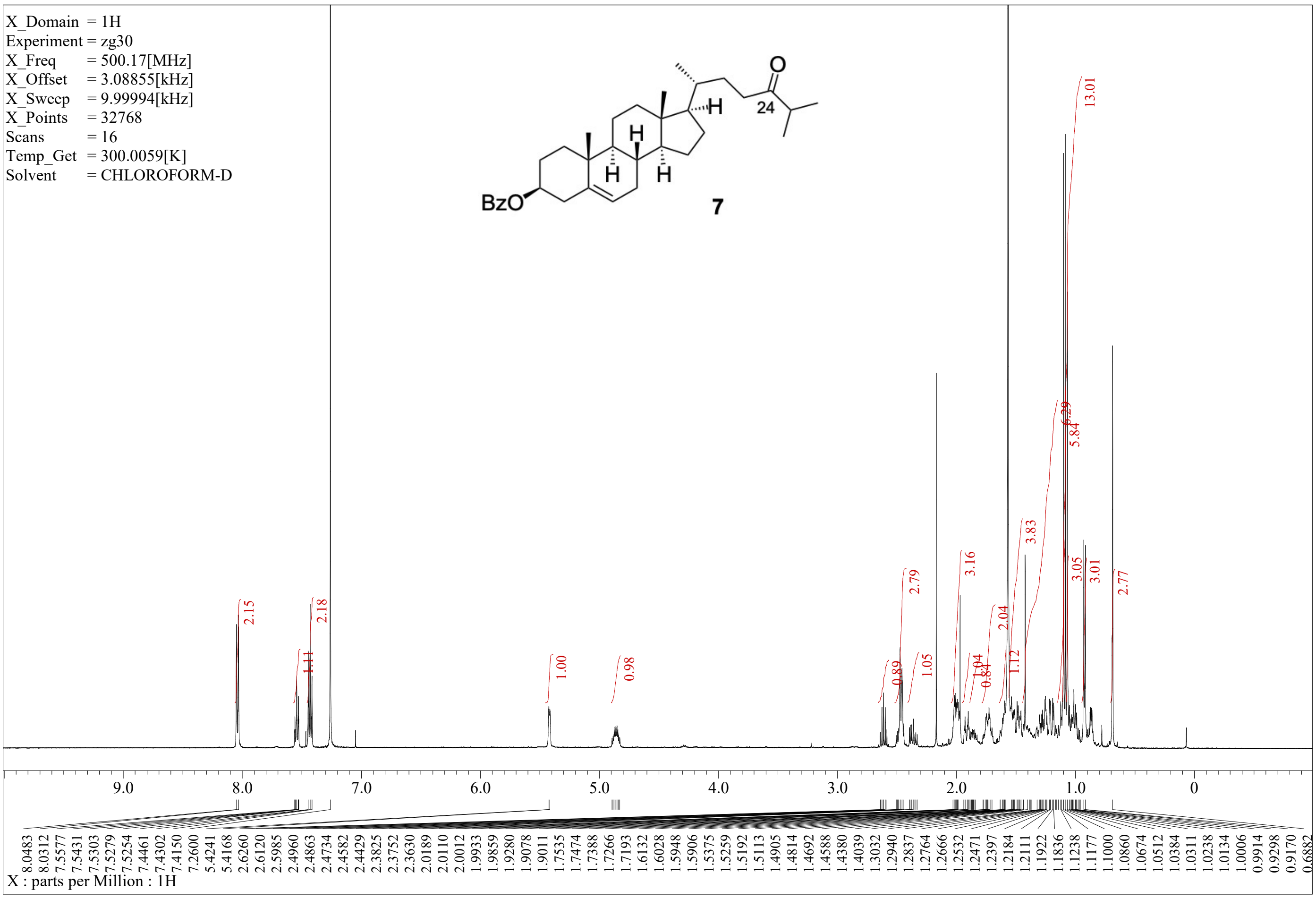
X_Domain = 1H
Experiment = zg30
X_Freq = 500.17[MHz]
X_Offset = 3.08855[kHz]
X_Sweep = 9.99994[kHz]
X_Points = 32768
Scans = 16
Temp_Get = 300.0059[K]
Solvent = CHLOROFORM-D
BzO
H
24
O
7
2.15
1.11
2.18
1.00
0.98
0.89
2.79
1.05
3.16
1.04
0.84
2.04
1.12
3.83
6.29
5.84
3.05
3.01
2.77
13.01
9.0
8.0
7.0
6.0
5.0
4.0
3.0
2.0
1.0
0
8.0483 8.0312 7.5577 7.5431 7.5303 7.5279 7.5254 7.4461 7.4302 7.4150 7.2600 5.4241 5.4168 2.6260 2.6120 2.5985 2.4960 2.4863 2.4734 2.4582 2.4429 2.3825 2.3752 2.3630 2.0189 2.0110 2.0012 1.9933 1.9859 1.9280 1.9078 1.9011 1.7535 1.7474 1.7388 1.7266 1.7193 1.6132 1.6028 1.5948 1.5906 1.5375 1.5259 1.5192 1.5113 1.4905 1.4814 1.4692 1.4588 1.4380 1.4039 1.3032 1.2940 1.2837 1.2764 1.2666 1.2532 1.2471 1.2397 1.2184 1.2111 1.1922 1.1836 1.1238 1.1177 1.1000 1.0860 1.0674 1.0512 1.0384 1.0311 1.0238 1.0134 1.0006 0.9914 0.9298 0.9170 0.6882
X : parts per Million : 1H

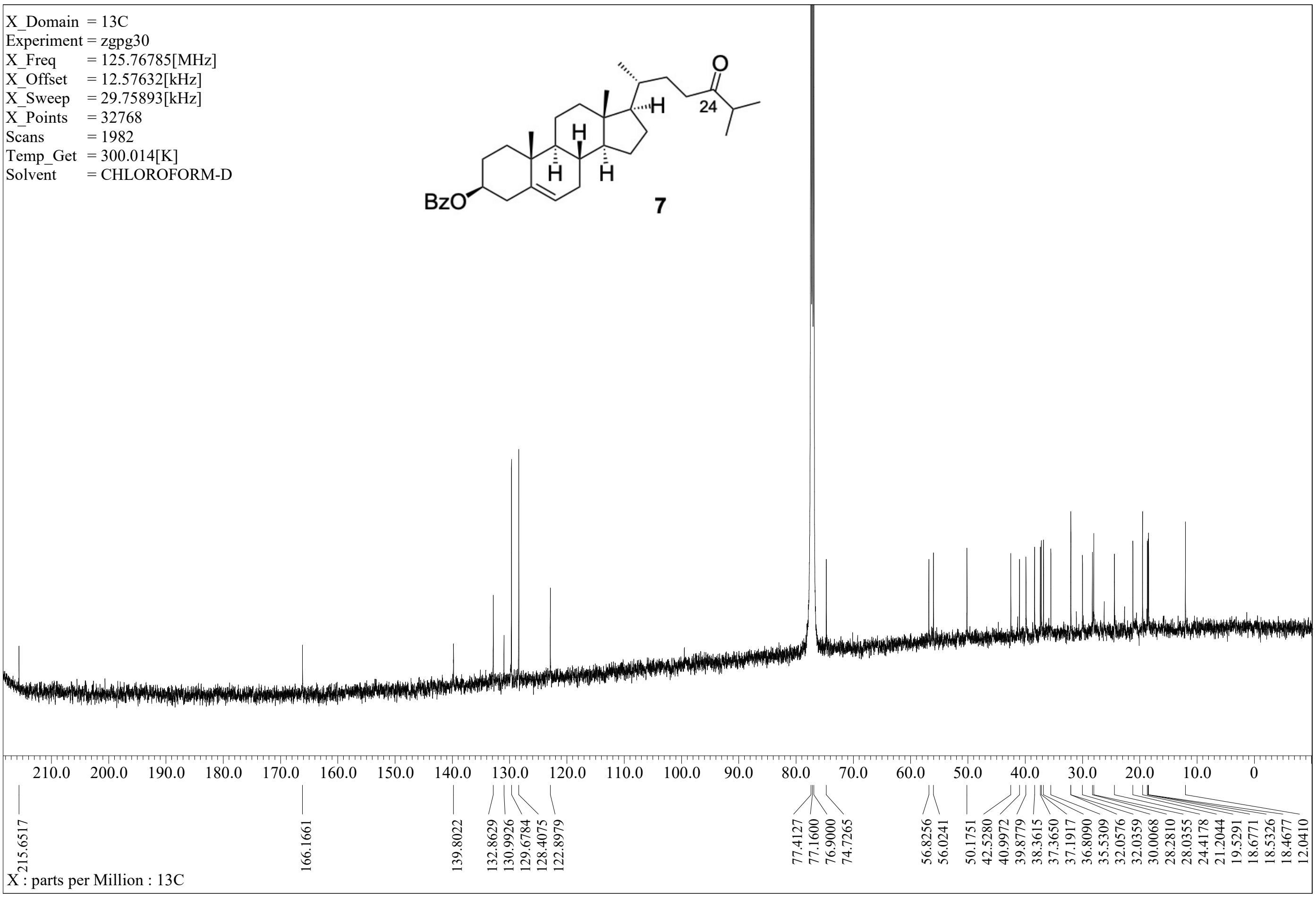
X_Domain = 13C
Experiment = zgpg30
X_Freq = 125.76785[MHz]
X_Offset = 12.57632[kHz]
X_Sweep = 29.75893[kHz]
X_Points = 32768
Scans = 1982
Temp_Get = 300.014[K]
Solvent = CHLOROFORM-D
BzO
H
24
O
7
210.0 200.0 190.0 180.0 170.0 160.0 150.0 140.0 130.0 120.0 110.0 100.0 90.0 80.0 70.0 60.0 50.0 40.0 30.0 20.0 10.0 0
215.6517 166.1661 139.8022 132.8629 130.9926 129.6784 128.4075 122.8979 77.4127 77.1600 76.9000 74.7265 56.8256 56.0241 50.1751 42.5280 40.9972 39.8779 38.3615 37.3650 37.1917 36.8090 35.5309 32.0576 32.0359 30.0068 28.2810 28.0355 24.4178 21.2044 19.5291 18.6771 18.5326 18.4677 12.0410
X : parts per Million : 13C

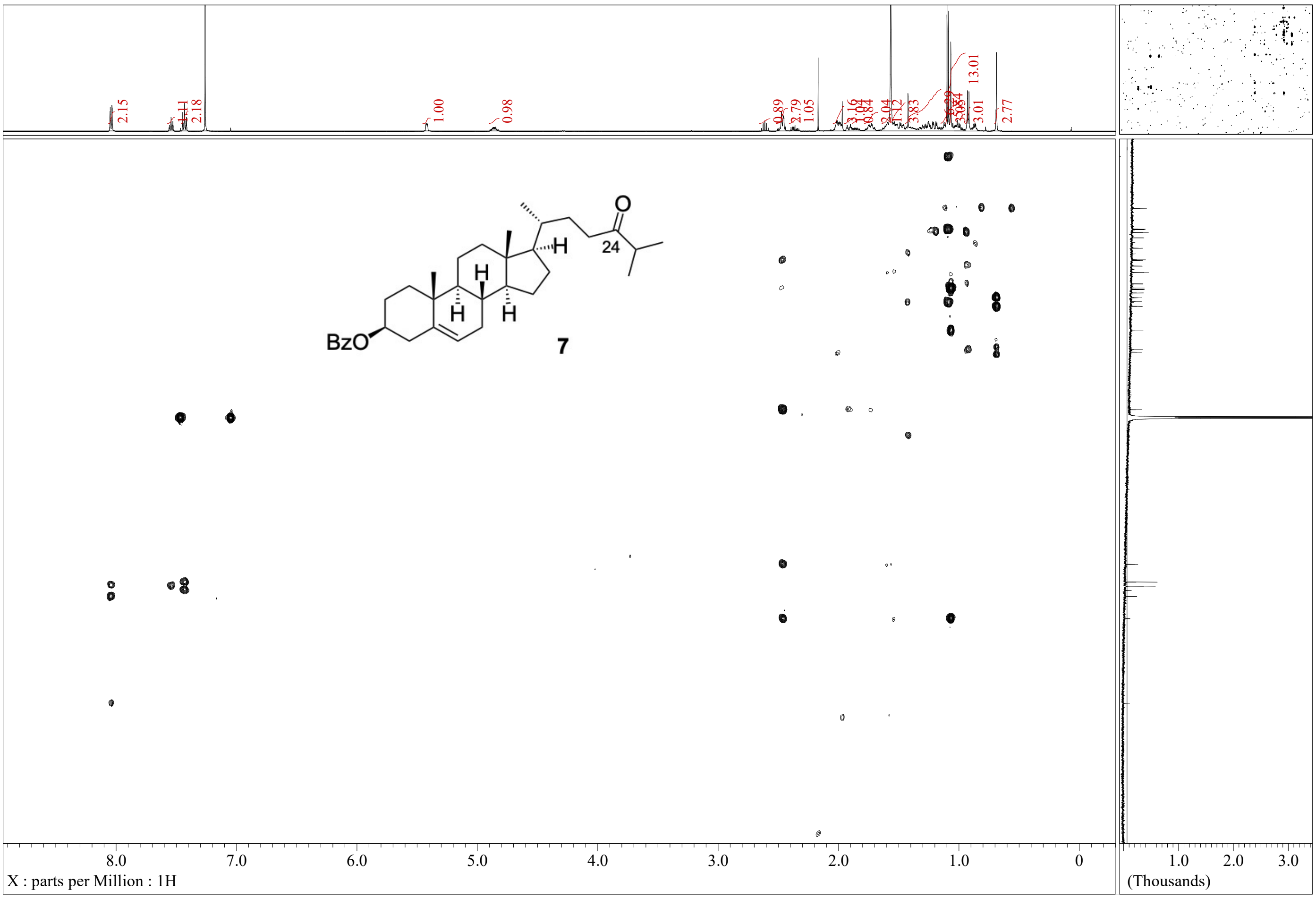

BzO
24
7
8.0
7.0
6.0
5.0
4.0
3.0
2.0
1.0
0
X : parts per Million : 1H
1.0
2.0
3.0
(Thousands)

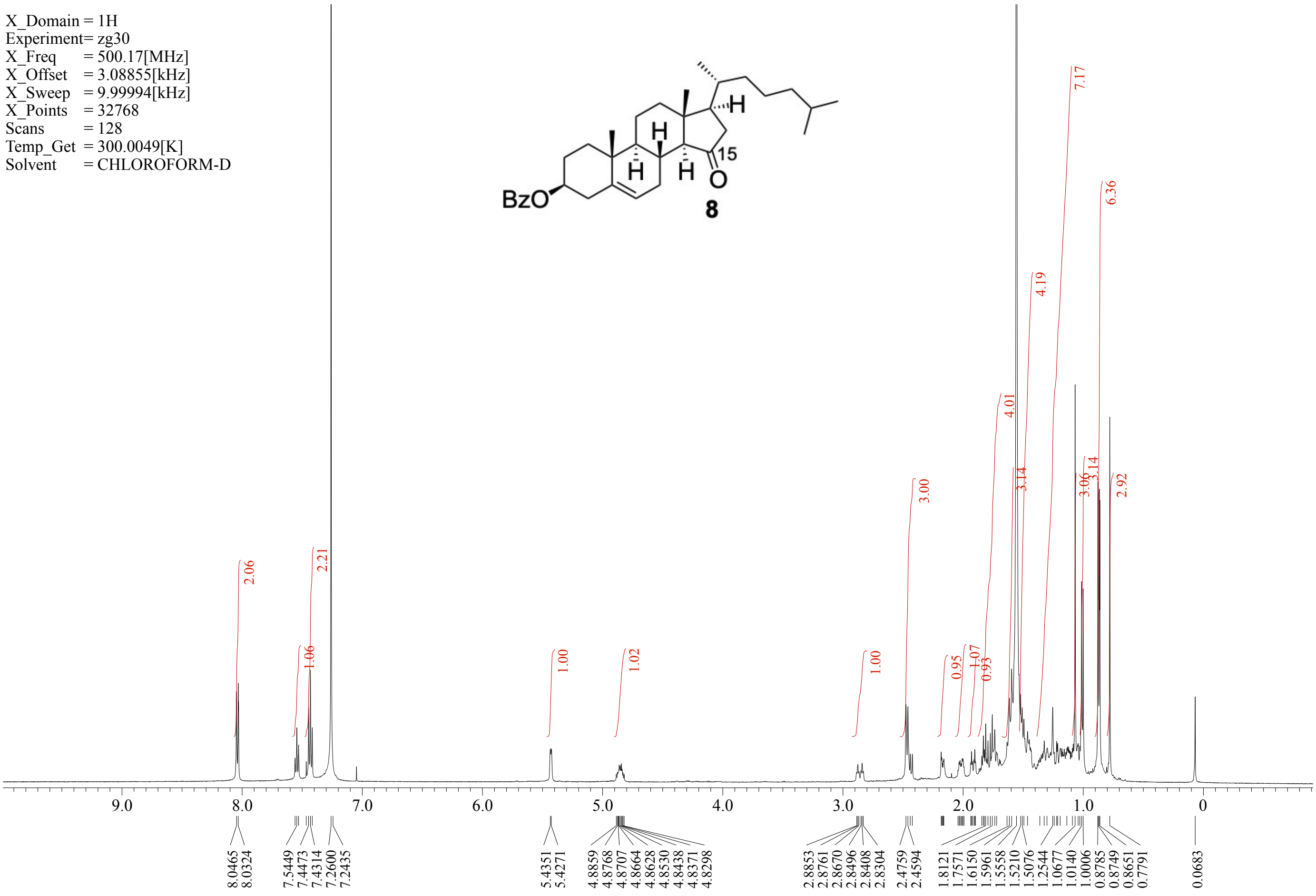

X_Domain = 1H
Experiment= zg30
X_Freq = 500.17[MHz]
X_Offset = 3.08855[kHz]
X_Sweep = 9.99994[kHz]
X_Points = 32768
Scans = 128
Temp_Get = 300.0049[K]
Solvent = CHLOROFORM-D
BzO
15
8
9.0
8.0
7.0
6.0
5.0
4.0
3.0
2.0
1.0
0
8.0465
8.0324
7.5449
7.4473
7.4314
7.2600
7.2435
5.4351
5.4271
4.8859
4.8768
4.8707
4.8664
4.8628
4.8530
4.8438
4.8371
4.8298
2.8853
2.8761
2.8670
2.8496
2.8408
2.8304
2.4759
2.4594
1.8121
1.7571
1.6150
1.5961
1.5558
1.5210
1.5076
1.2544
1.0677
1.0140
1.0006
0.8785
0.8749
0.8651
0.7791
0.0683

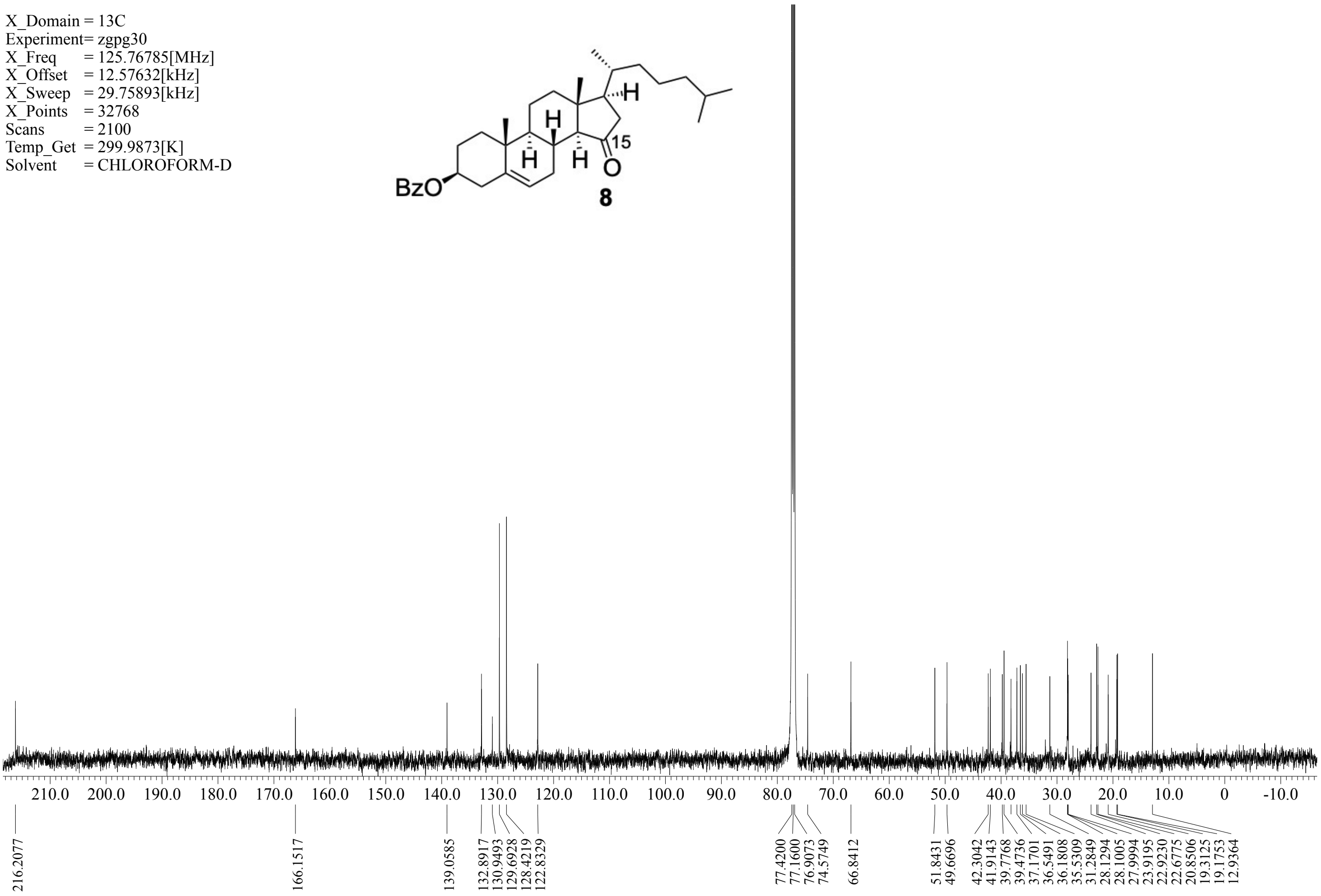
X_Domain = 13C
Experiment= zgpg30
X_Freq = 125.76785[MHz]
X_Offset = 12.57632[kHz]
X_Sweep = 29.75893[kHz]
X_Points = 32768
Scans = 2100
Temp_Get = 299.9873[K]
Solvent = CHLOROFORM-D
H
H
H
H
15
O
BzO
8
210.0 200.0 190.0 180.0 170.0 160.0 150.0 140.0 130.0 120.0 110.0 100.0 90.0 80.0 70.0 60.0 50.0 40.0 30.0 20.0 10.0 0 -10.0
216.2077
166.1517
139.0585
132.8917
130.9493
129.6928
128.4219
122.8329
77.4200
77.1600
76.9073
74.5749
66.8412
51.8431
49.6696
42.3042
41.9143
39.7768
39.4736
37.1701
36.5491
36.1808
35.5309
31.2849
28.1294
28.1005
27.9994
23.9195
22.9230
22.6775
20.8506
19.3125
19.1753
12.9364

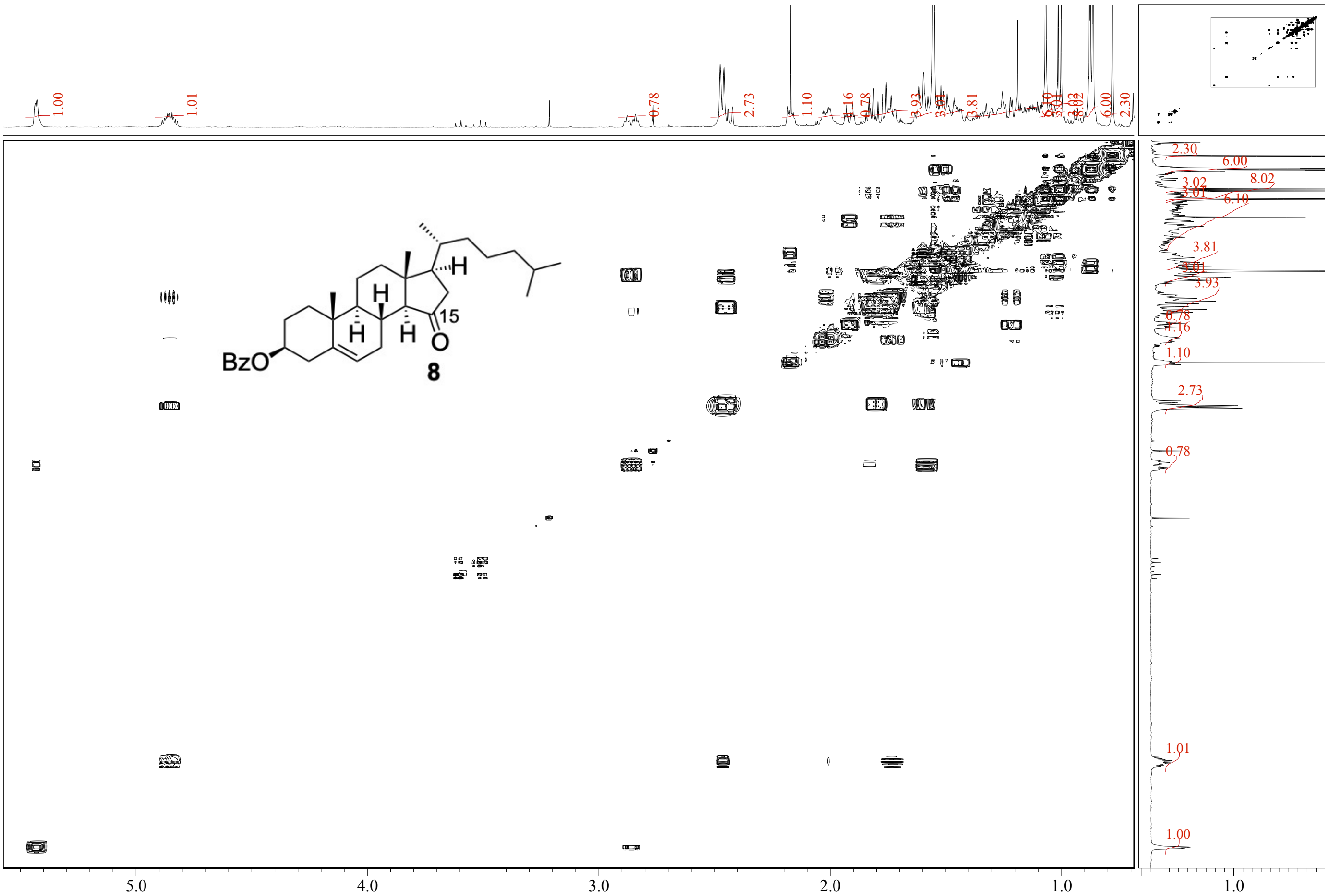
H
H
H
H
15
O
BzO
8
1.00
1.01
0.78
2.73
1.10
3.81
6.00
2.30
8.02
3.02
3.01
6.10
3.93
5.0
4.0
3.0
2.0
1.0

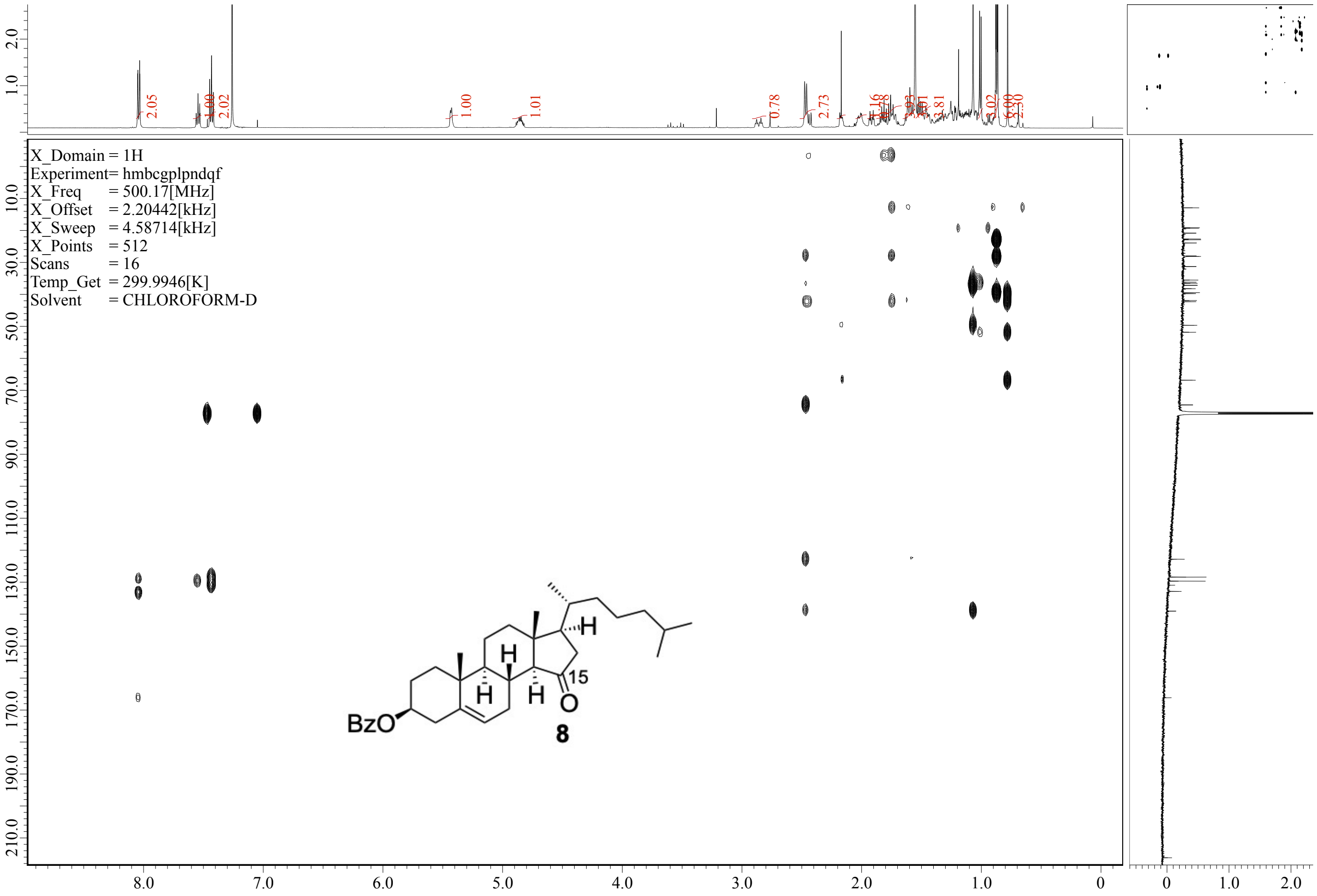
X_Domain = 1H
Experiment= hmbcgplpndqf
X_Freq = 500.17[MHz]
X_Offset = 2.20442[kHz]
X_Sweep = 4.58714[kHz]
X_Points = 512
Scans = 16
Temp_Get = 299.9946[K]
Solvent = CHLOROFORM-D
BzO
15
8

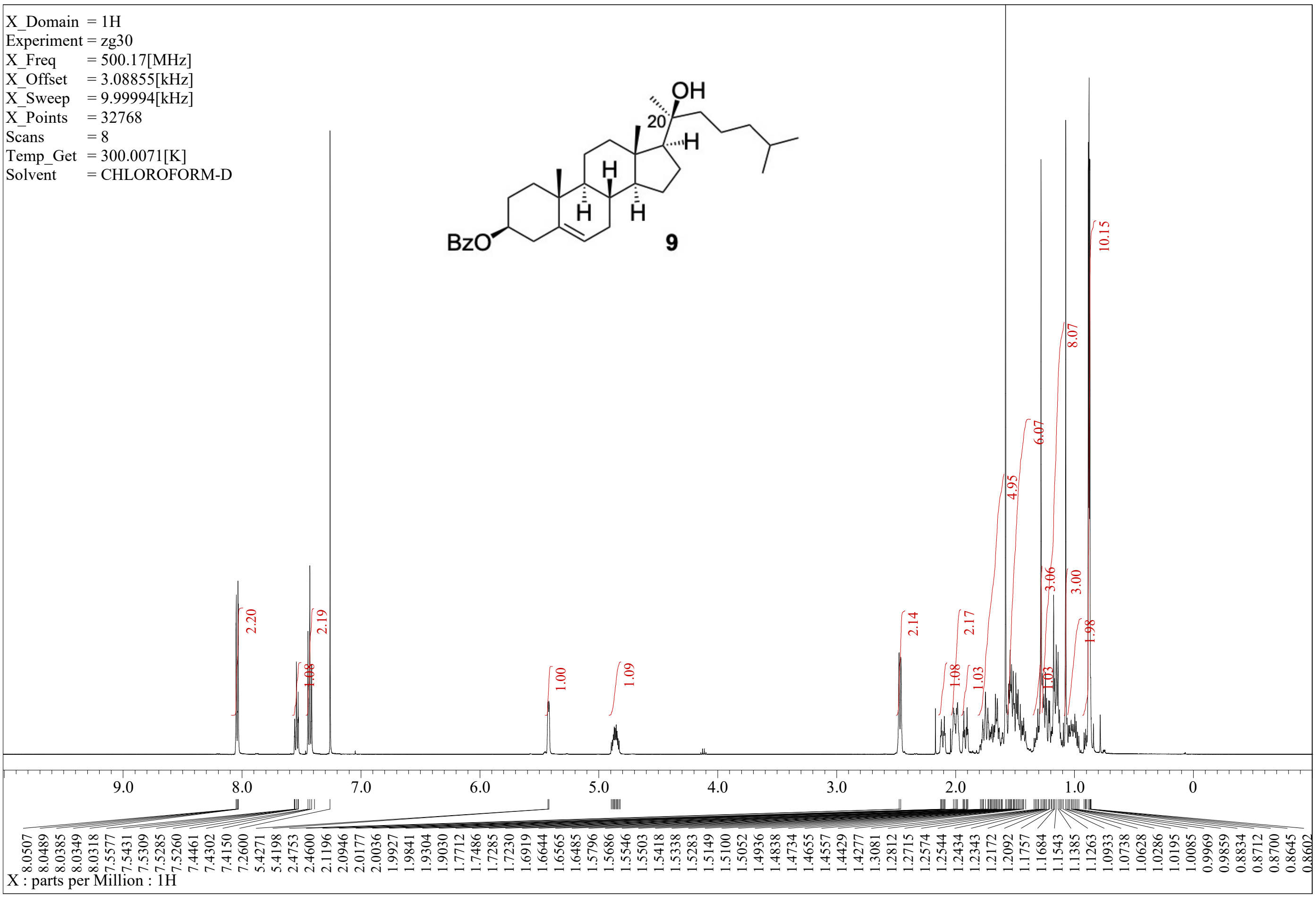
X_Domain = 1H
Experiment = zg30
X_Freq = 500.17[MHz]
X_Offset = 3.08855[kHz]
X_Sweep = 9.99994[kHz]
X_Points = 32768
Scans = 8
Temp_Get = 300.0071[K]
Solvent = CHLOROFORM-D
OH
20
BzO
9
8.0507
8.0489
8.0385
8.0349
8.0318
7.5577
7.5431
7.5309
7.5285
7.5260
7.4461
7.4302
7.4150
7.2600
5.4271
5.4198
2.4753
2.4600
2.1196
2.0946
2.0177
2.0036
1.9927
1.9841
1.9304
1.9030
1.7712
1.7486
1.7285
1.7230
1.6919
1.6644
1.6565
1.6485
1.5796
1.5686
1.5546
1.5503
1.5418
1.5338
1.5283
1.5149
1.5100
1.5052
1.4936
1.4838
1.4734
1.4655
1.4557
1.4429
1.4277
1.3081
1.2812
1.2715
1.2574
1.2544
1.2434
1.2343
1.2172
1.2092
1.1757
1.1684
1.1543
1.1385
1.1263
1.0933
1.0738
1.0628
1.0286
1.0195
1.0085
0.9969
0.9859
0.8834
0.8712
0.8700
0.8645
0.8602
X : parts per Million : 1H

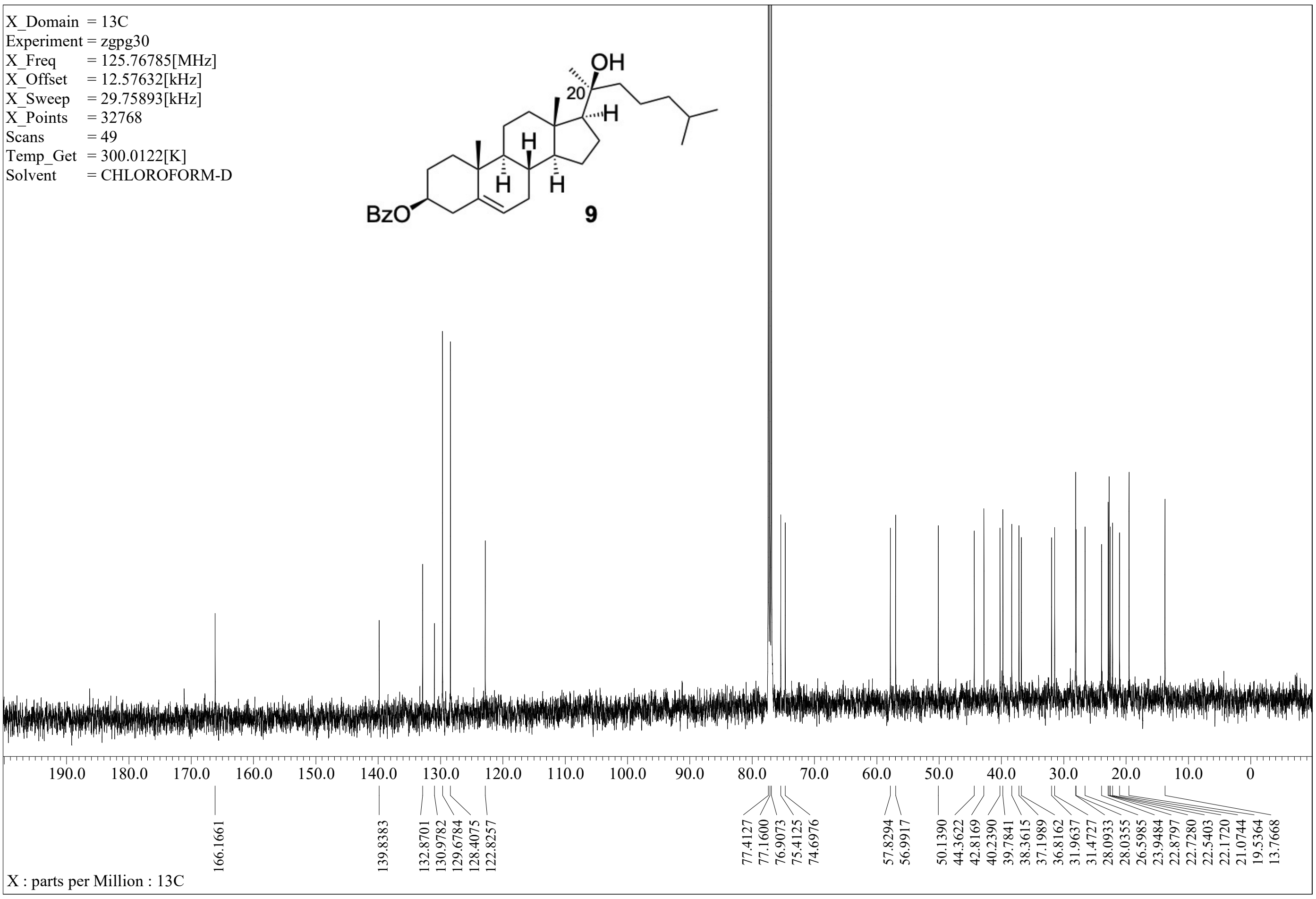
X_Domain = 13C
Experiment = zgpg30
X_Freq = 125.76785[MHz]
X_Offset = 12.57632[kHz]
X_Sweep = 29.75893[kHz]
X_Points = 32768
Scans = 49
Temp_Get = 300.0122[K]
Solvent = CHLOROFORM-D
OH
20
H
H
H
H
BzO
9
166.1661
139.8383
132.8701
130.9782
129.6784
128.4075
122.8257
77.4127
77.1600
76.9073
75.4125
74.6976
57.8294
56.9917
50.1390
44.3622
42.8169
40.2390
39.7841
38.3615
37.1989
36.8162
31.9637
31.4727
28.0933
28.0355
26.5985
23.9484
22.8797
22.7280
22.5403
22.1720
21.0744
19.5364
13.7668
X : parts per Million : 13C

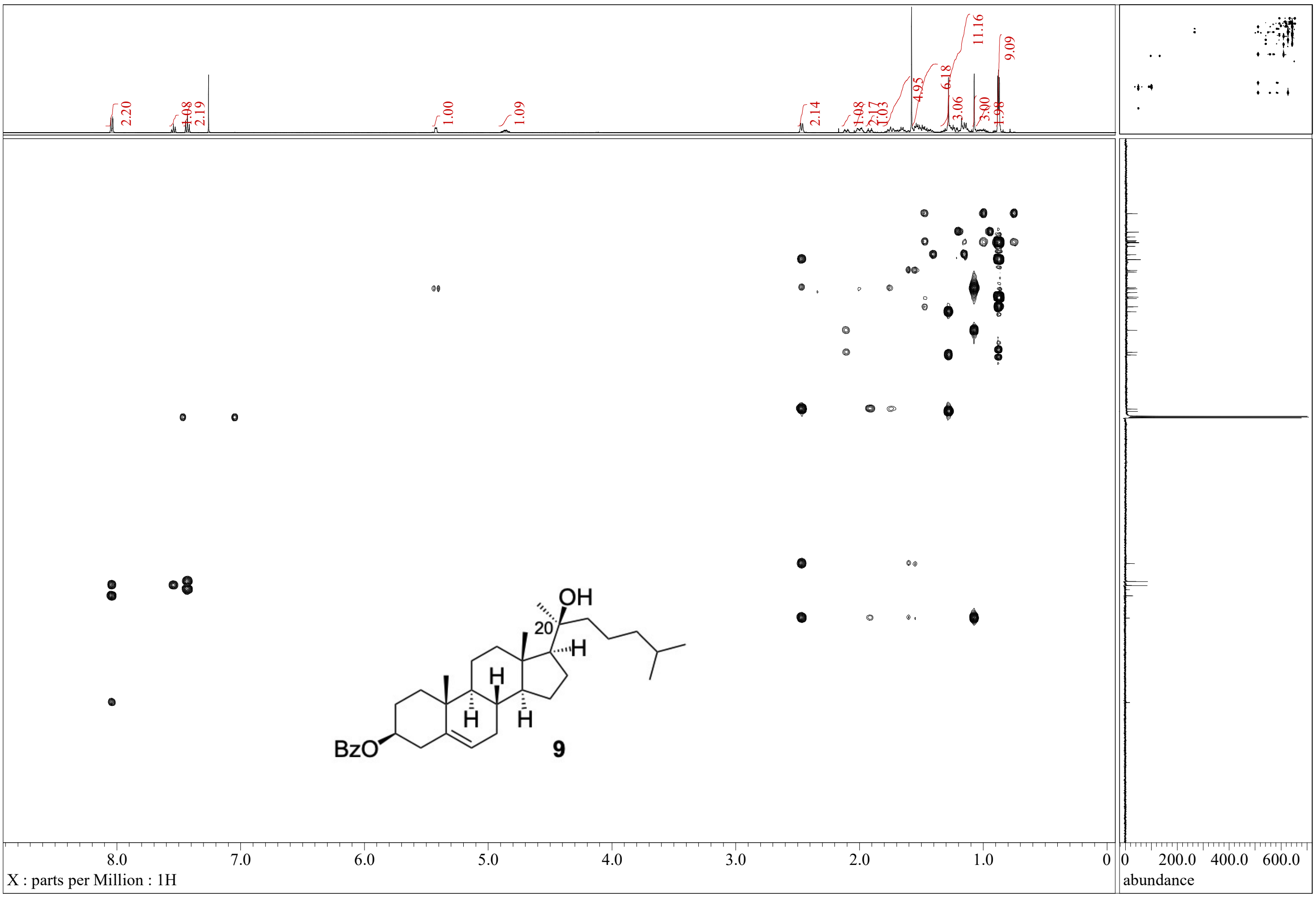
2.20
1.08
2.19
1.00
1.09
2.14
1.08
2.17
1.03
4.95
6.18
3.06
11.16
3.00
9.09
OH
20
H
H
H
H
BzO
9
X : parts per Million : 1H
abundance

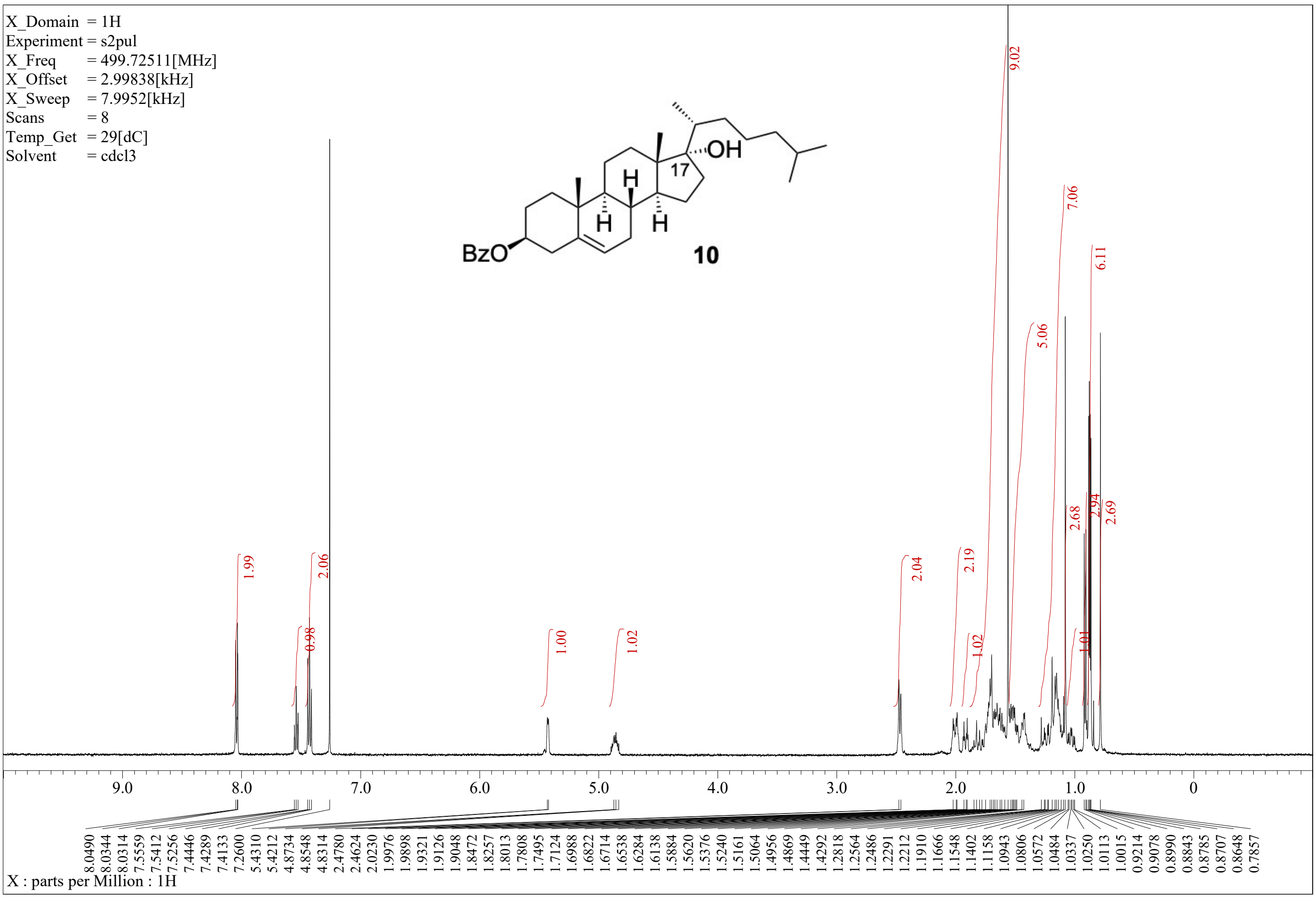
X_Domain = 1H
Experiment = s2pul
X_Freq = 499.72511[MHz]
X_Offset = 2.99838[kHz]
X_Sweep = 7.9952[kHz]
Scans = 8
Temp_Get = 29[dC]
Solvent = cdcl3
BzO
OH
17
H
10
X : parts per Million : 1H

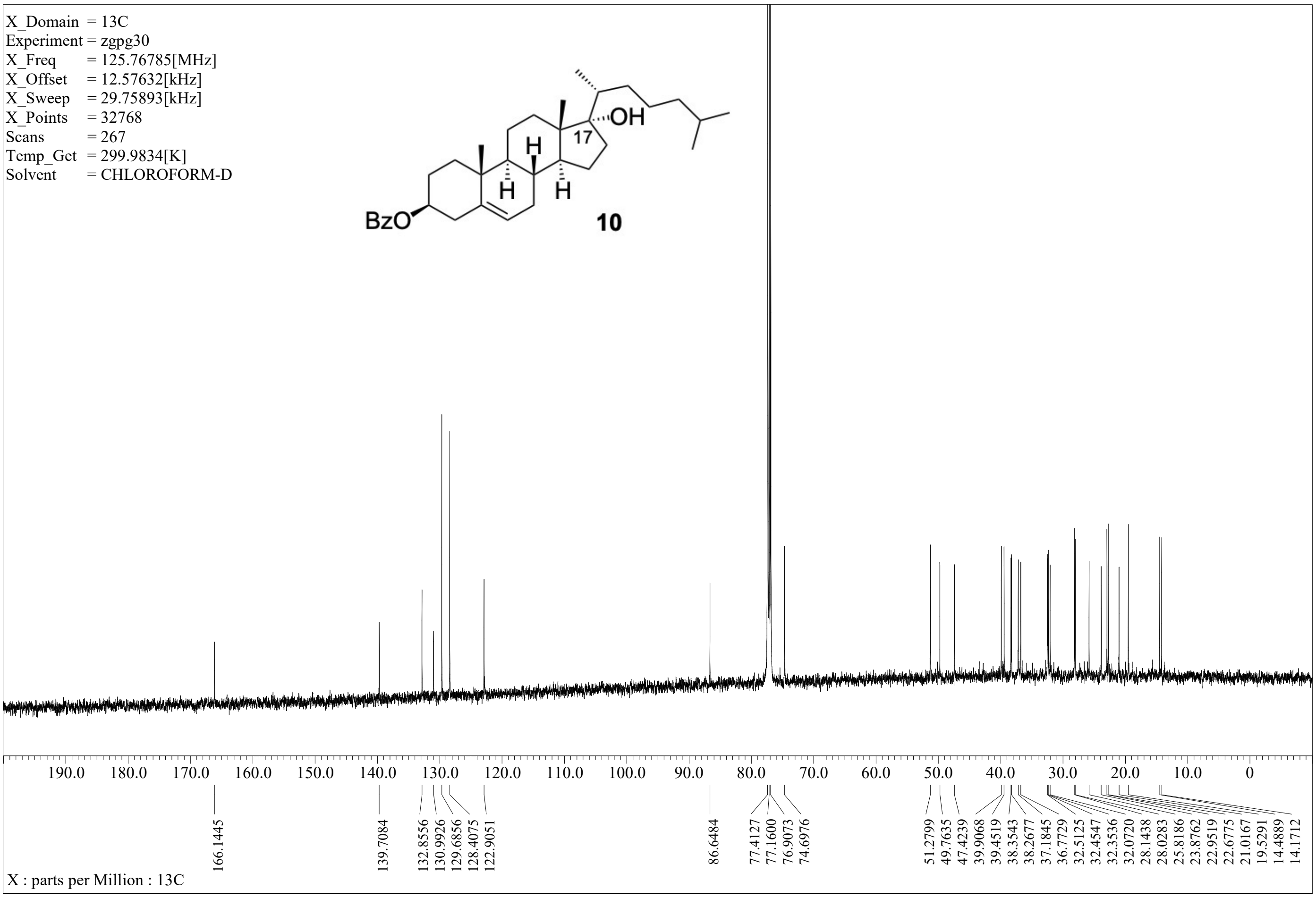
X_Domain = 13C
Experiment = zgpg30
X_Freq = 125.76785[MHz]
X_Offset = 12.57632[kHz]
X_Sweep = 29.75893[kHz]
X_Points = 32768
Scans = 267
Temp_Get = 299.9834[K]
Solvent = CHLOROFORM-D
BzO
OH
17
H
10
X : parts per Million : 13C

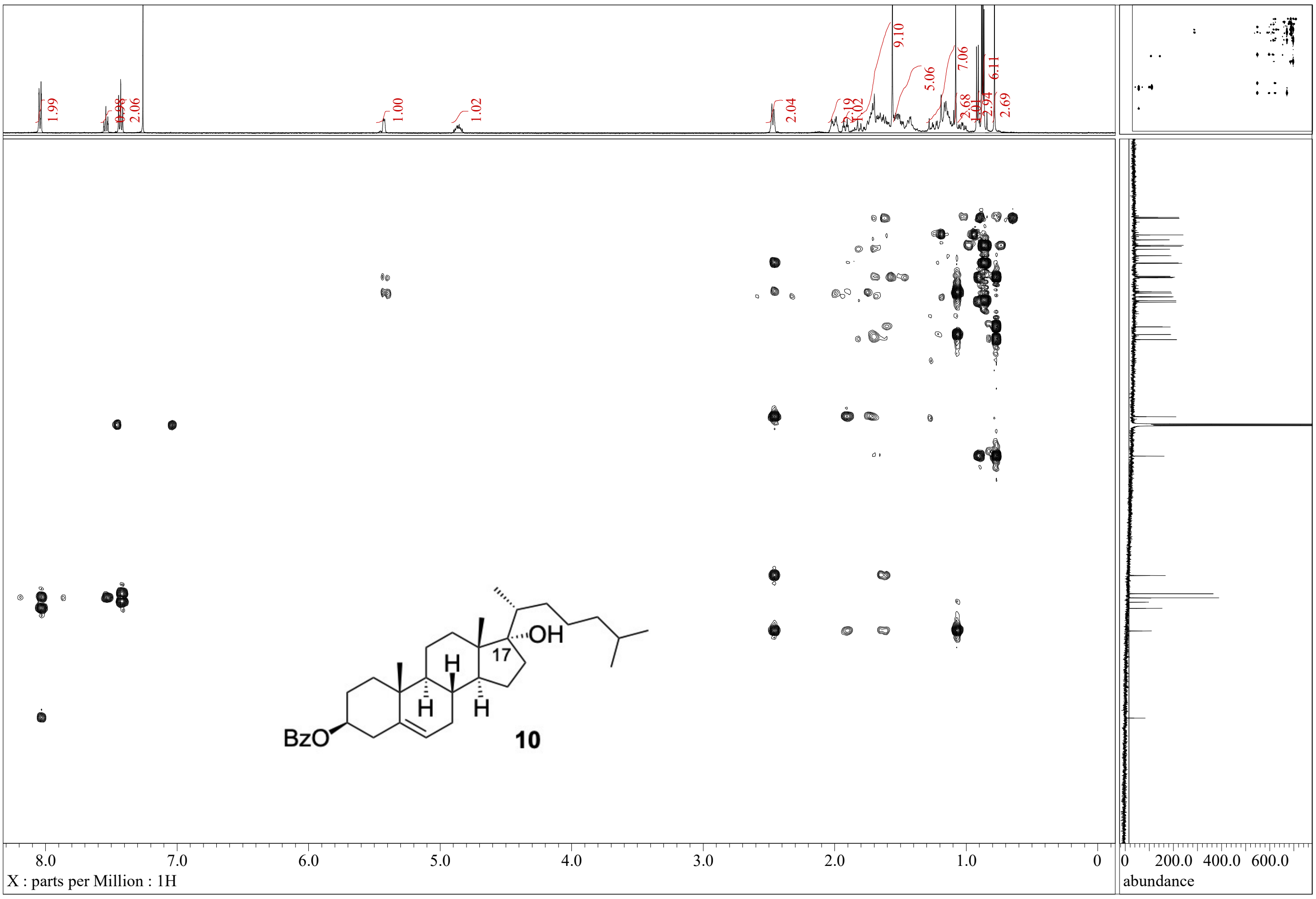
1.99
0.98
2.06
1.00
1.02
2.04
9.10
5.06
7.06
6.11
2.69
OH
17
H
H
H
BzO
10
8.0
7.0
6.0
5.0
4.0
3.0
2.0
1.0
0
X : parts per Million : 1H
0
200.0
400.0
600.0
abundance

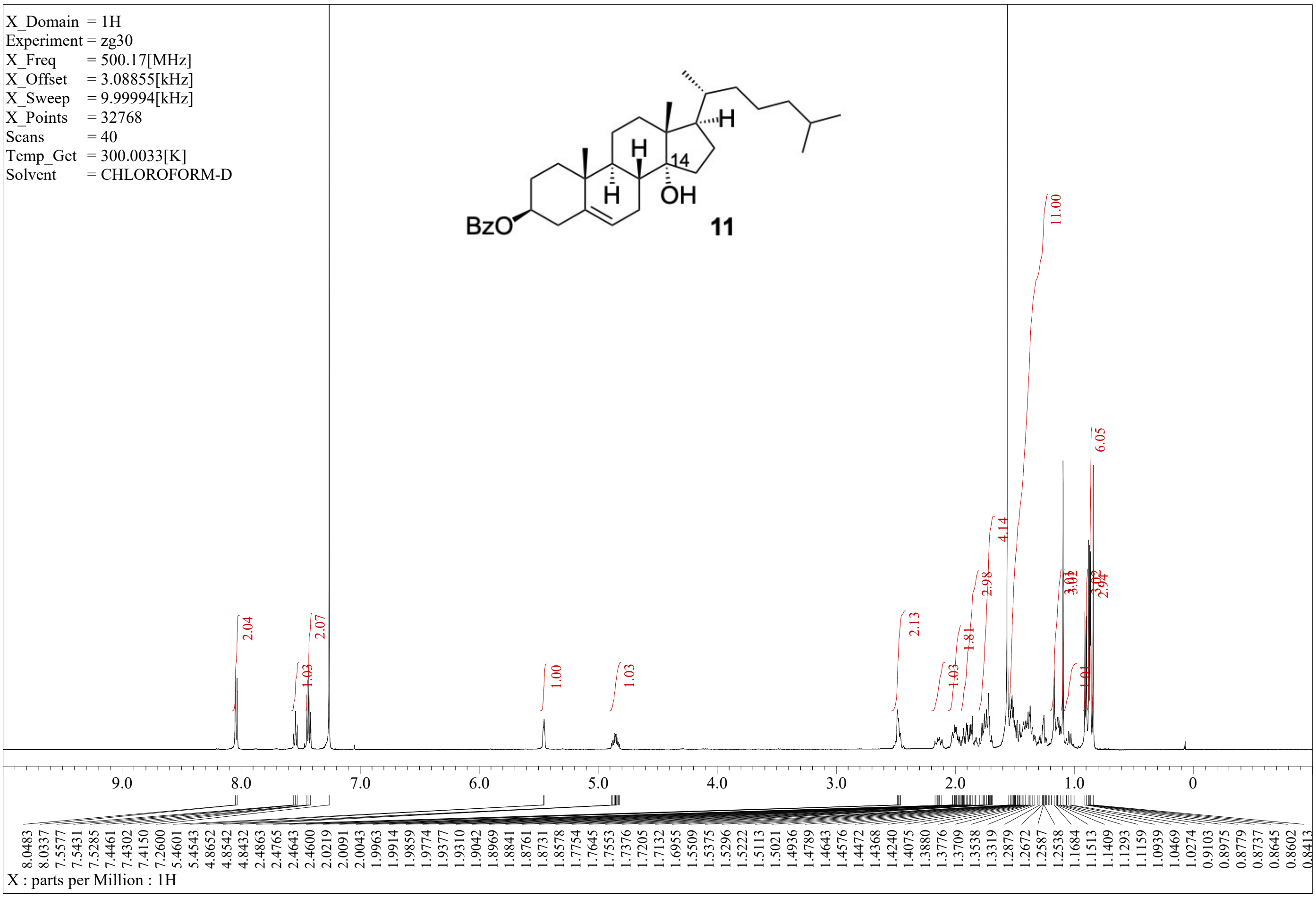
X_Domain = 1H
Experiment = zg30
X_Freq = 500.17[MHz]
X_Offset = 3.08855[kHz]
X_Sweep = 9.99994[kHz]
X_Points = 32768
Scans = 40
Temp_Get = 300.0033[K]
Solvent = CHLOROFORM-D
H
H
14
H
OH
BzO
11
2.04
1.03
2.07
1.00
1.03
2.13
1.03
1.81
2.98
4.14
11.00
1.01
6.05
9.0
8.0
7.0
6.0
5.0
4.0
3.0
2.0
1.0
0
8.0483 8.0337 7.5577 7.5431 7.5285 7.4461 7.4302 7.4150 7.2600 5.4601 5.4543 4.8652 4.8542 4.8432 2.4863 2.4765 2.4643 2.4600 2.0219 2.0091 2.0043 1.9963 1.9914 1.9859 1.9774 1.9377 1.9310 1.9042 1.8969 1.8841 1.8761 1.8731 1.8578 1.7754 1.7645 1.7553 1.7376 1.7205 1.7132 1.6955 1.5509 1.5375 1.5296 1.5222 1.5113 1.5021 1.4936 1.4789 1.4643 1.4576 1.4472 1.4368 1.4240 1.4075 1.3880 1.3776 1.3709 1.3538 1.3319 1.2879 1.2672 1.2587 1.2538 1.1684 1.1513 1.1409 1.1293 1.1159 1.0939 1.0469 1.0274 0.9103 0.8975 0.8779 0.8737 0.8645 0.8602 0.8413
X : parts per Million : 1H

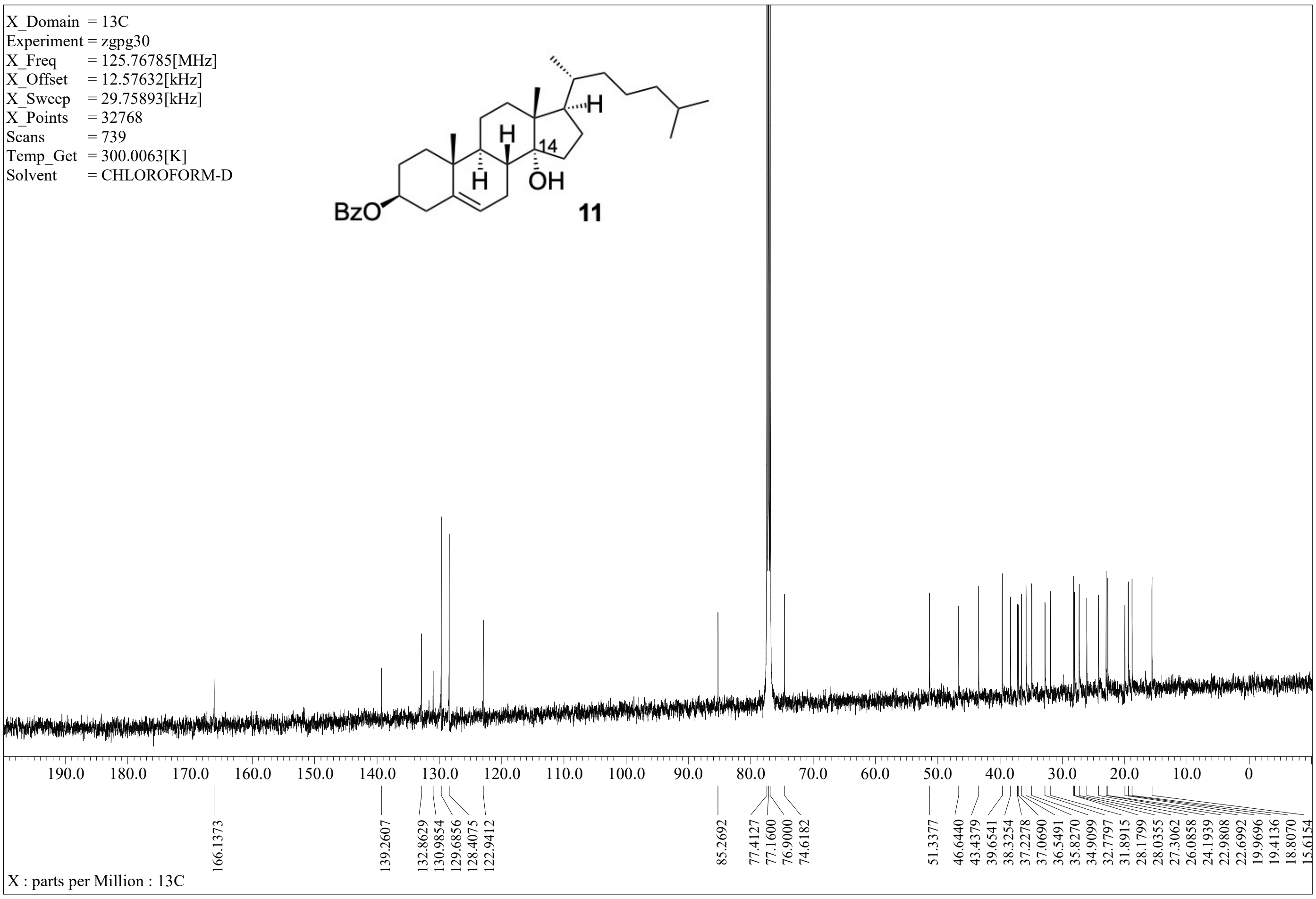
X_Domain = 13C
Experiment = zgpg30
X_Freq = 125.76785[MHz]
X_Offset = 12.57632[kHz]
X_Sweep = 29.75893[kHz]
X_Points = 32768
Scans = 739
Temp_Get = 300.0063[K]
Solvent = CHLOROFORM-D
BzO
H
H
H
14
OH
11
190.0 180.0 170.0 160.0 150.0 140.0 130.0 120.0 110.0 100.0 90.0 80.0 70.0 60.0 50.0 40.0 30.0 20.0 10.0 0
166.1373
139.2607
132.8629
130.9854
129.6856
128.4075
122.9412
85.2692
77.4127
77.1600
76.9000
74.6182
51.3377
46.6440
43.4379
39.6541
38.3254
37.2278
37.0690
36.5491
35.8270
34.9099
32.7797
31.8915
28.1799
28.0355
27.3062
26.0858
24.1939
22.9808
22.6992
19.9696
19.4136
18.8070
15.6154
X : parts per Million : 13C

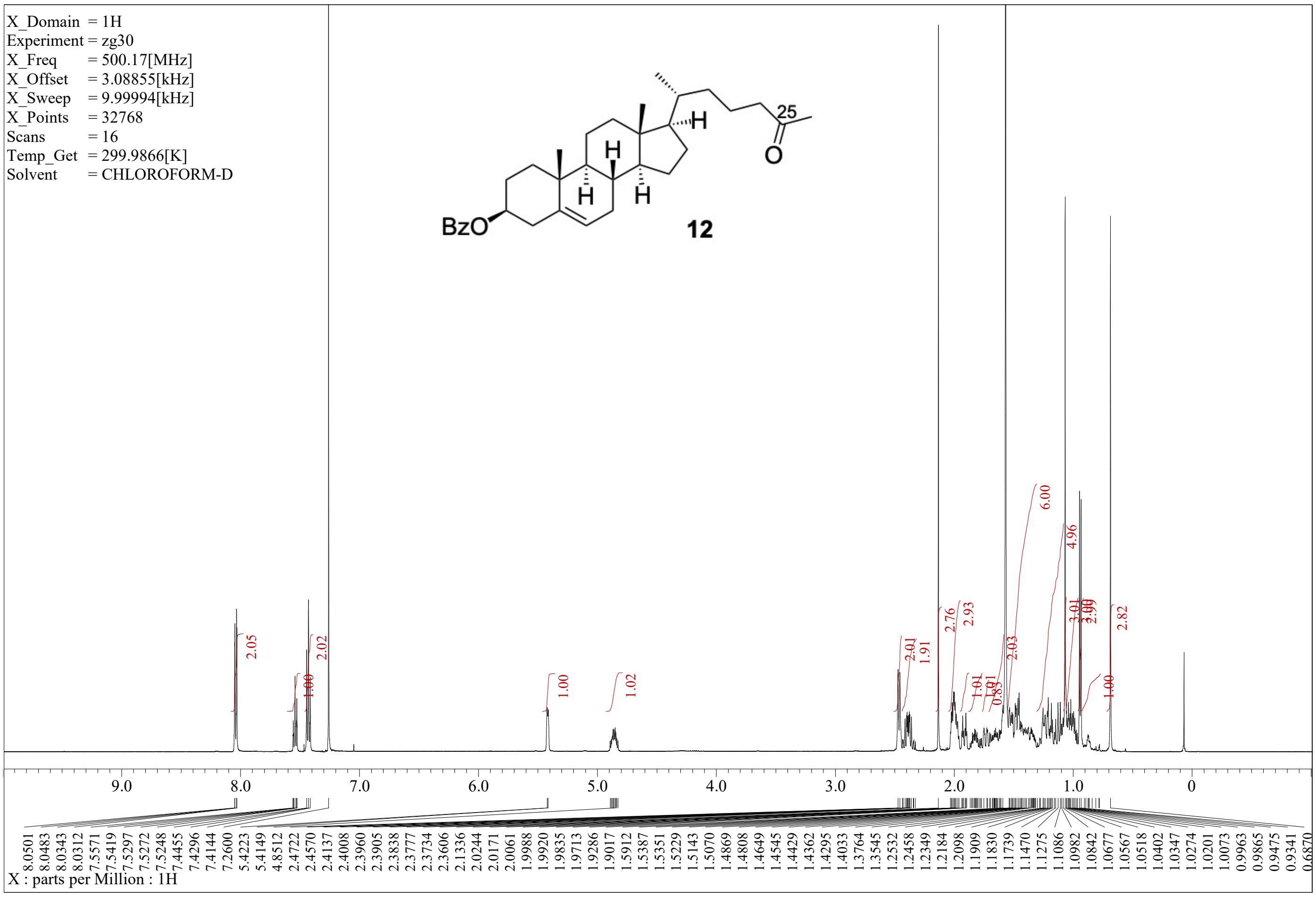
X_Domain = 1H
Experiment = zg30
X_Freq = 500.17[MHz]
X_Offset = 3.08855[kHz]
X_Sweep = 9.99994[kHz]
X_Points = 32768
Scans = 16
Temp_Get = 299.9866[K]
Solvent = CHLOROFORM-D
BzO
H
H
H
H
25
O
12
2.05
1.00
2.02
1.00
1.02
2.01
1.91
2.76
2.93
1.01
1.01
0.85
2.03
6.00
4.96
3.01
2.82
1.00
9.0 8.0 7.0 6.0 5.0 4.0 3.0 2.0 1.0 0
8.0501
8.0483
8.0343
8.0312
7.5571
7.5419
7.5297
7.5272
7.5248
7.4455
7.4296
7.4144
7.2600
5.4223
5.4149
4.8512
2.4722
2.4570
2.4137
2.4008
2.3960
2.3905
2.3838
2.3777
2.3734
2.3606
2.1336
2.0244
2.0171
2.0061
1.9988
1.9920
1.9835
1.9713
1.9286
1.9017
1.5912
1.5387
1.5351
1.5229
1.5143
1.5070
1.4869
1.4808
1.4649
1.4545
1.4429
1.4362
1.4295
1.4033
1.3764
1.3545
1.2532
1.2458
1.2349
1.2184
1.2098
1.1909
1.1830
1.1739
1.1470
1.1275
1.1086
1.0982
1.0842
1.0677
1.0567
1.0518
1.0402
1.0347
1.0274
1.0201
1.0073
0.9963
0.9865
0.9475
0.9341
0.6870
X : parts per Million : 1H

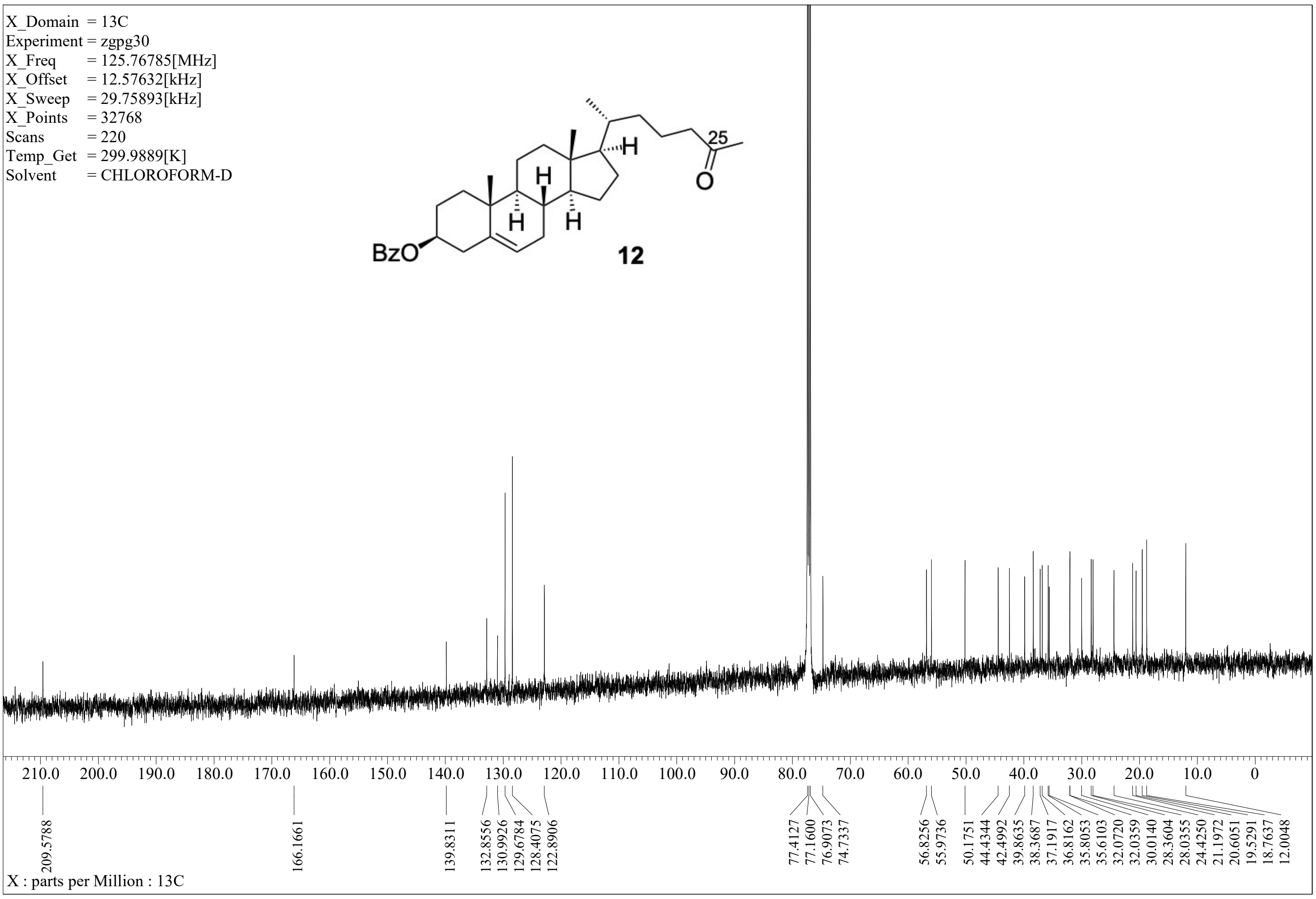
X_Domain = 13C
Experiment = zgpg30
X_Freq = 125.76785[MHz]
X_Offset = 12.57632[kHz]
X_Sweep = 29.75893[kHz]
X_Points = 32768
Scans = 220
Temp_Get = 299.9889[K]
Solvent = CHLOROFORM-D
BzO
25
12
209.5788
166.1661
139.8311
132.8556
130.9926
129.6784
128.4075
122.8906
77.4127
77.1600
76.9073
74.7337
56.8256
55.9736
50.1751
44.4344
42.4992
39.8635
38.3687
37.1917
36.8162
35.8053
35.6103
32.0720
32.0359
30.0140
28.3604
28.0355
24.4250
21.1972
20.6051
19.5291
18.7637
12.0048
X : parts per Million : 13C

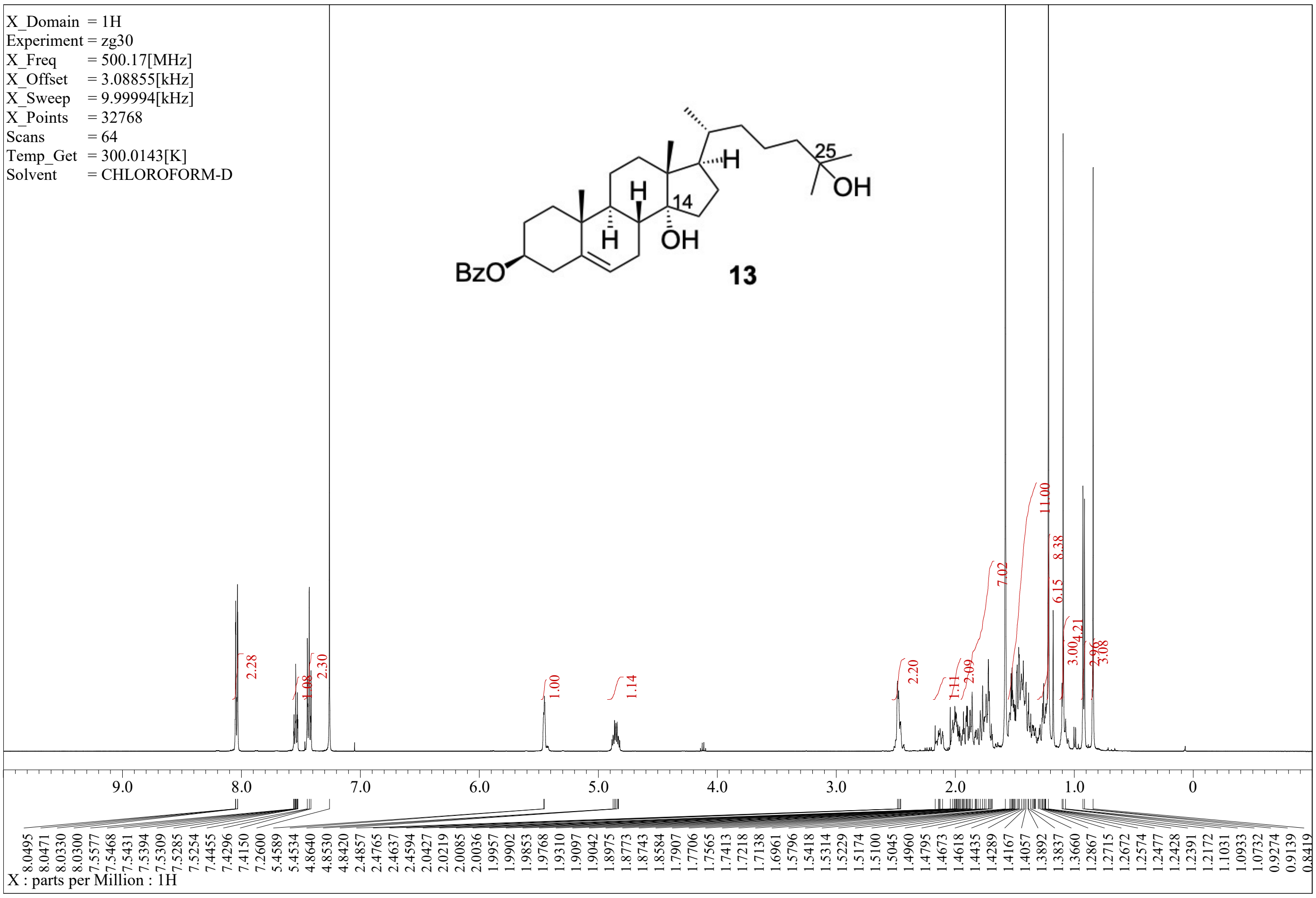
X_Domain = 1H
Experiment = zg30
X_Freq = 500.17[MHz]
X_Offset = 3.08855[kHz]
X_Sweep = 9.99994[kHz]
X_Points = 32768
Scans = 64
Temp_Get = 300.0143[K]
Solvent = CHLOROFORM-D
BzO
25
14
OH
13
X : parts per Million : 1H

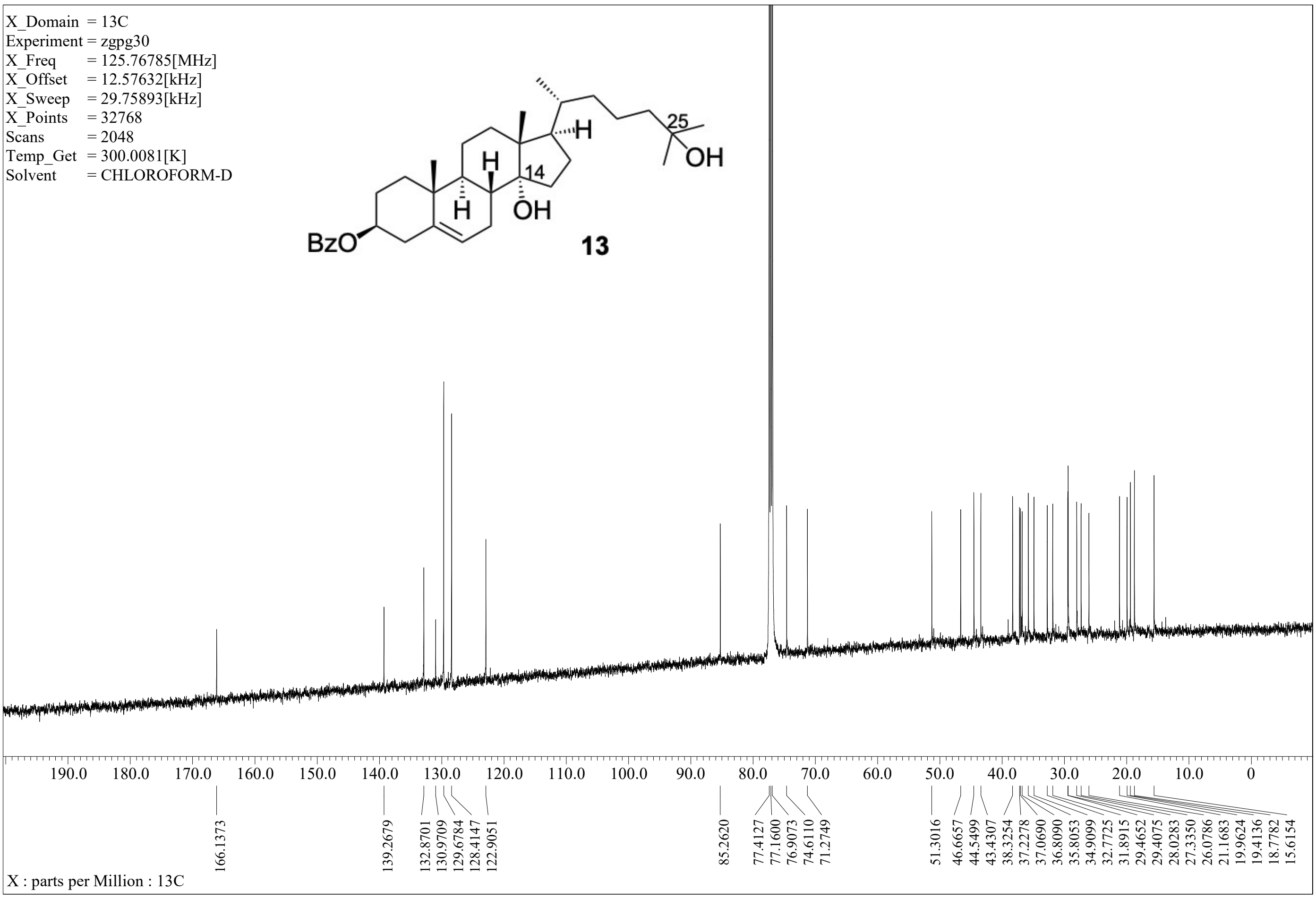
X_Domain = 13C
Experiment = zgpg30
X_Freq = 125.76785[MHz]
X_Offset = 12.57632[kHz]
X_Sweep = 29.75893[kHz]
X_Points = 32768
Scans = 2048
Temp_Get = 300.0081[K]
Solvent = CHLOROFORM-D
BzO
H
H
H
OH
OH
14
25
13
190.0 180.0 170.0 160.0 150.0 140.0 130.0 120.0 110.0 100.0 90.0 80.0 70.0 60.0 50.0 40.0 30.0 20.0 10.0 0
166.1373
139.2679
132.8701
130.9709
129.6784
128.4147
122.9051
85.2620
77.4127
77.1600
76.9073
74.6110
71.2749
51.3016
46.6657
44.5499
43.4307
38.3254
37.2278
37.0690
36.8090
35.8053
34.9099
32.7725
31.8915
29.4652
29.4075
28.0283
27.3350
26.0786
21.1683
19.9624
19.4136
18.7782
15.6154
X : parts per Million : 13C

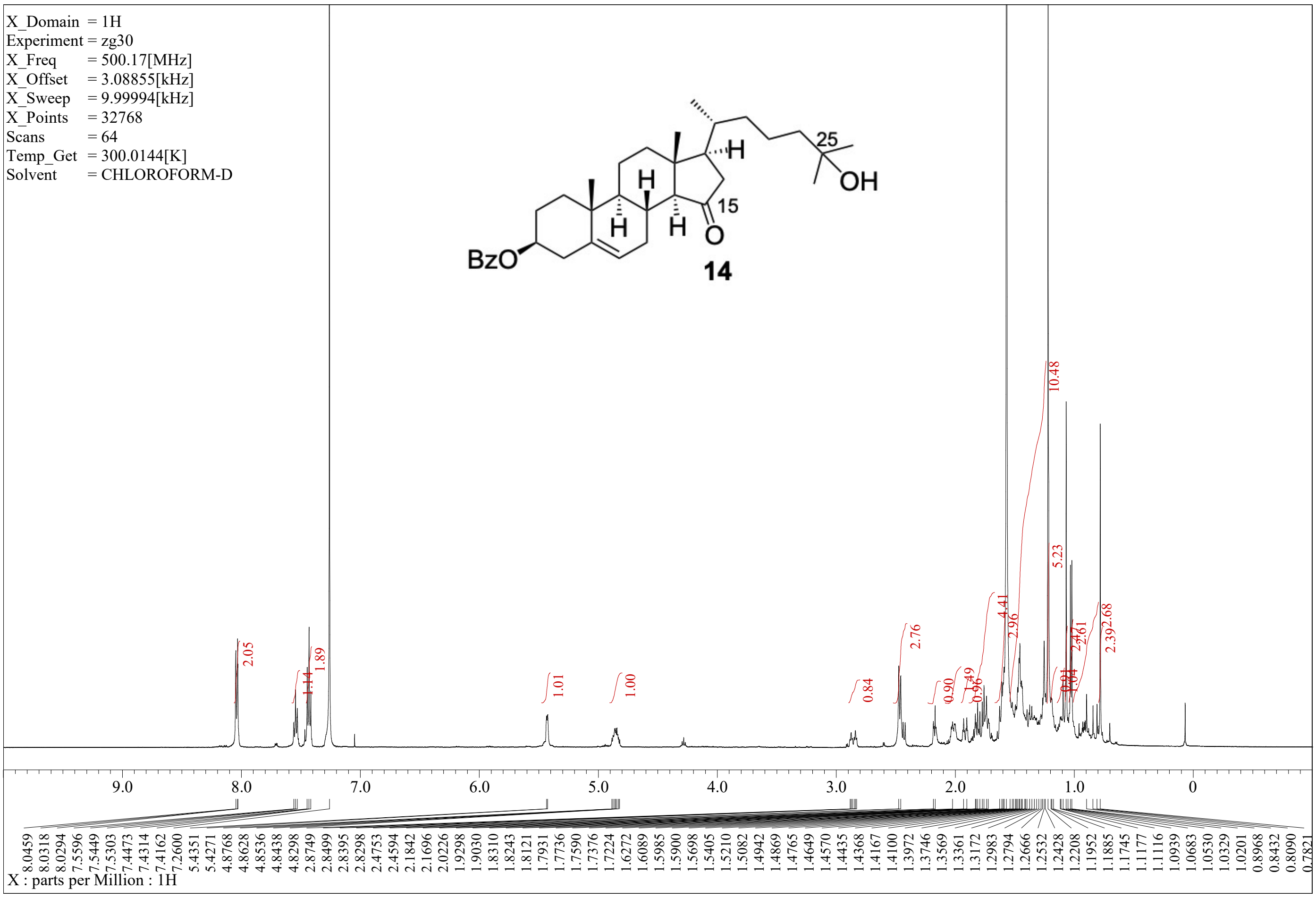
X_Domain = 1H
Experiment = zg30
X_Freq = 500.17[MHz]
X_Offset = 3.08855[kHz]
X_Sweep = 9.99994[kHz]
X_Points = 32768
Scans = 64
Temp_Get = 300.0144[K]
Solvent = CHLOROFORM-D
BzO
H
H
H
H
O
OH
15
25
14
2.05
1.14
1.89
1.01
1.00
0.84
2.76
0.90
0.96
1.49
4.41
2.96
10.48
5.23
2.47
2.61
2.39
2.68
9.0 8.0 7.0 6.0 5.0 4.0 3.0 2.0 1.0 0
8.0459
8.0318
8.0294
7.5596
7.5449
7.5303
7.4473
7.4314
7.4162
7.2600
5.4351
5.4271
4.8768
4.8628
4.8536
4.8438
4.8298
2.8749
2.8499
2.8395
2.8298
2.4753
2.4594
2.1842
2.1696
2.0226
1.9298
1.9030
1.8310
1.8243
1.8121
1.7931
1.7736
1.7590
1.7376
1.7224
1.6272
1.6089
1.5985
1.5900
1.5698
1.5405
1.5210
1.5082
1.4942
1.4869
1.4765
1.4649
1.4570
1.4435
1.4368
1.4167
1.4100
1.3972
1.3746
1.3569
1.3361
1.3172
1.2983
1.2794
1.2666
1.2532
1.2428
1.2208
1.1952
1.1885
1.1745
1.1177
1.1116
1.0939
1.0683
1.0530
1.0329
1.0201
0.8968
0.8432
0.8090
0.7821
X : parts per Million : 1H

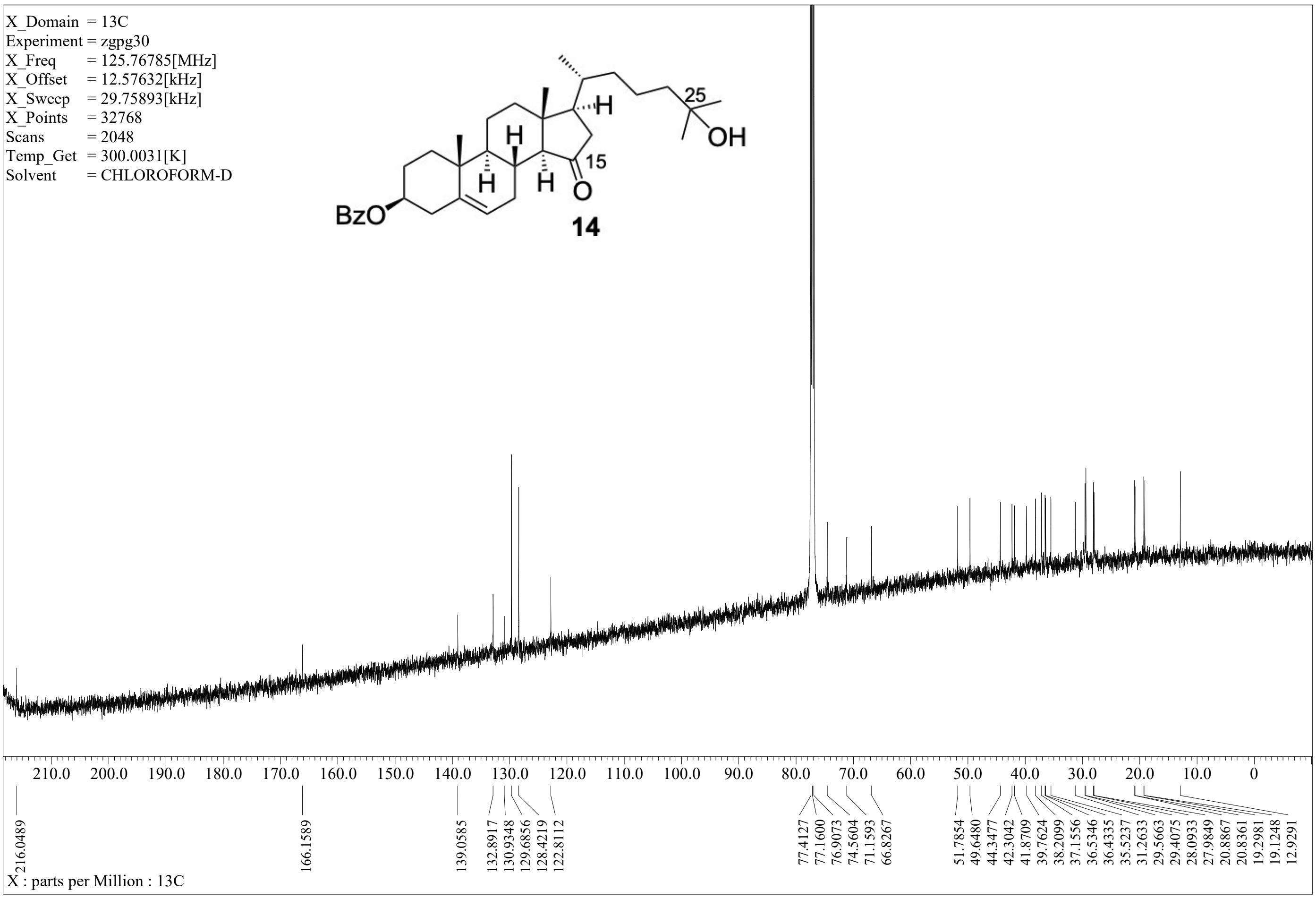
X_Domain = 13C
Experiment = zgpg30
X_Freq = 125.76785[MHz]
X_Offset = 12.57632[kHz]
X_Sweep = 29.75893[kHz]
X_Points = 32768
Scans = 2048
Temp_Get = 300.0031[K]
Solvent = CHLOROFORM-D
25
OH
15
BzO
14
216.0489
166.1589
139.0585
132.8917
130.9348
129.6856
128.4219
122.8112
77.4127
77.1600
76.9073
74.5604
71.1593
66.8267
51.7854
49.6480
44.3477
42.3042
41.8709
39.7624
38.2099
37.1556
36.5346
36.4335
35.5237
31.2633
29.5663
29.4075
28.0933
27.9849
20.8361
19.2981
19.1248
12.9291
X : parts per Million : 13C

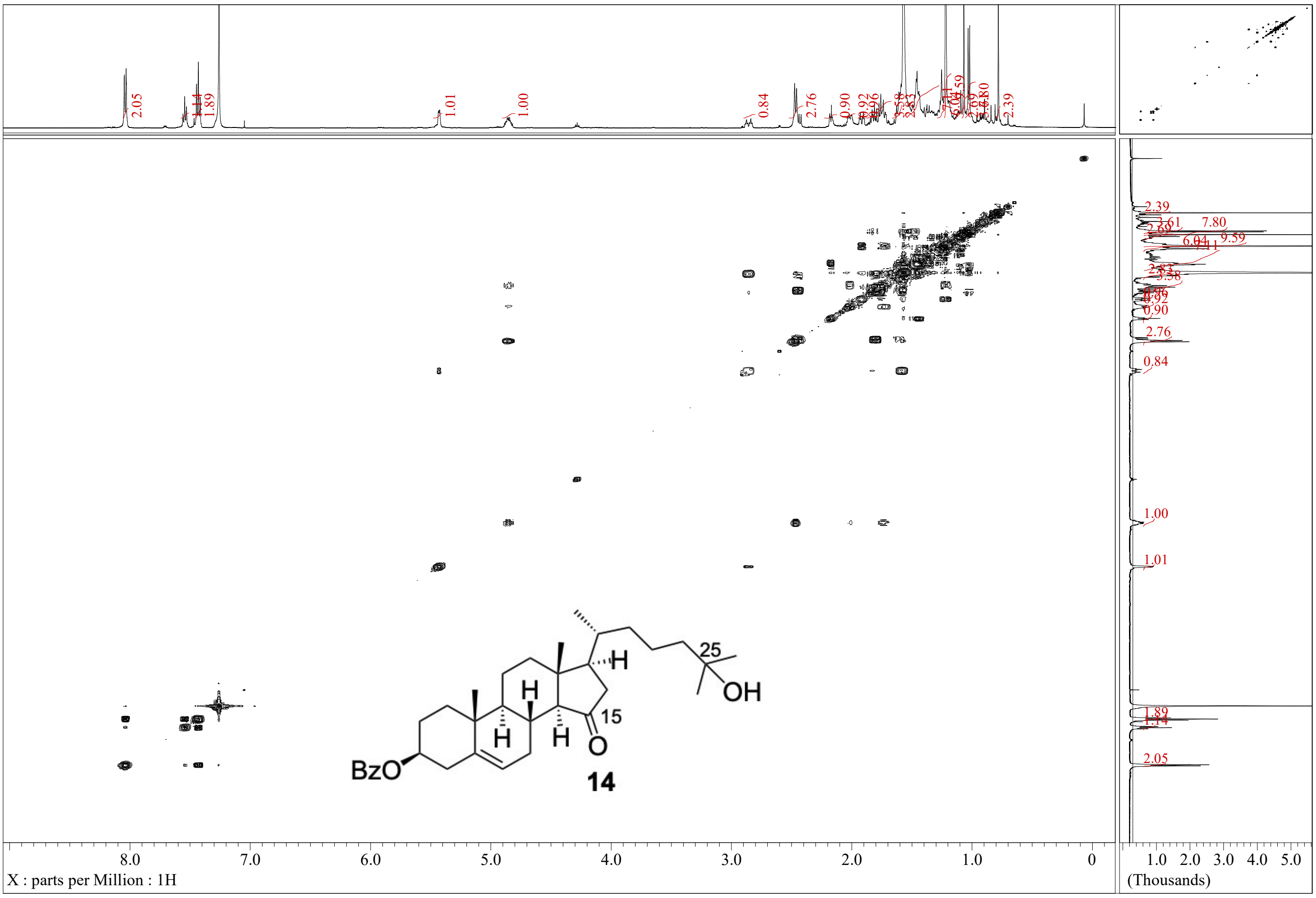
25
OH
15
BzO
14
X : parts per Million : 1H
(Thousands)

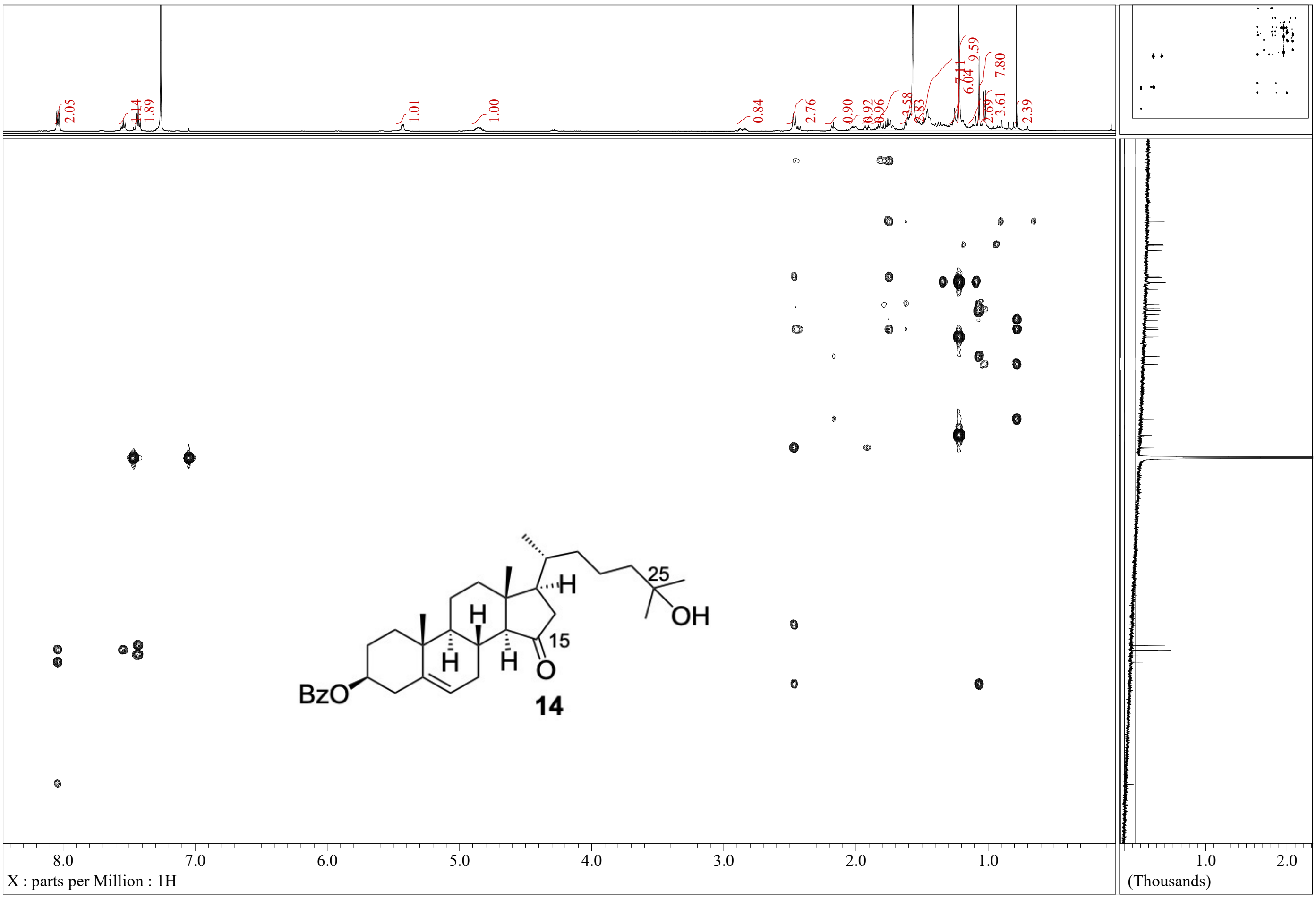
BzO
14
25
15
OH
2.05
1.14
1.89
1.01
1.00
0.84
2.76
0.90
0.92
0.96
3.58
3.83
7.11
6.04
9.59
2.69
3.61
7.80
2.39
8.0
7.0
6.0
5.0
4.0
3.0
2.0
1.0
X : parts per Million : 1H
1.0
2.0
(Thousands)

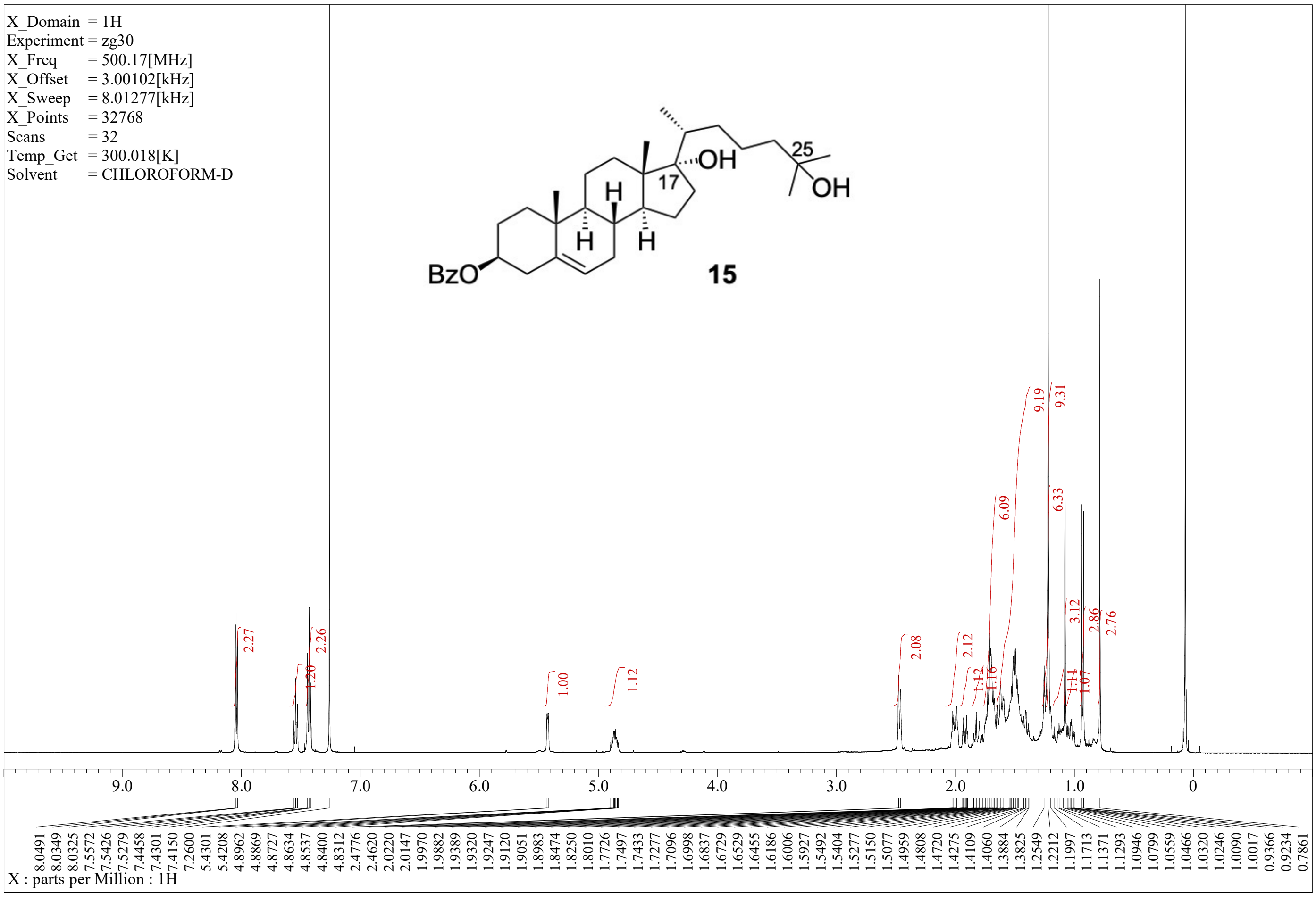
X_Domain = 1H
Experiment = zg30
X_Freq = 500.17[MHz]
X_Offset = 3.00102[kHz]
X_Sweep = 8.01277[kHz]
X_Points = 32768
Scans = 32
Temp_Get = 300.018[K]
Solvent = CHLOROFORM-D
BzO
15
17
OH
25
OH
2.27
1.20
2.26
1.00
1.12
2.08
2.12
1.12
1.16
6.09
9.19
9.31
6.33
1.14
1.07
3.12
2.86
2.76
9.0
8.0
7.0
6.0
5.0
4.0
3.0
2.0
1.0
0
8.0491
8.0349
8.0325
7.5572
7.5426
7.5279
7.4458
7.4301
7.4150
7.2600
5.4301
5.4208
4.8962
4.8869
4.8727
4.8634
4.8537
4.8400
4.8312
2.4776
2.4620
2.0220
2.0147
1.9970
1.9882
1.9389
1.9320
1.9247
1.9120
1.9051
1.8983
1.8474
1.8250
1.8010
1.7726
1.7497
1.7433
1.7277
1.7096
1.6998
1.6837
1.6729
1.6529
1.6455
1.6186
1.6006
1.5927
1.5492
1.5404
1.5277
1.5150
1.5077
1.4959
1.4808
1.4720
1.4275
1.4109
1.4060
1.3884
1.3825
1.2549
1.2212
1.1997
1.1713
1.1371
1.1293
1.0946
1.0799
1.0559
1.0466
1.0320
1.0246
1.0090
1.0017
0.9366
0.9234
0.7861
X : parts per Million : 1H

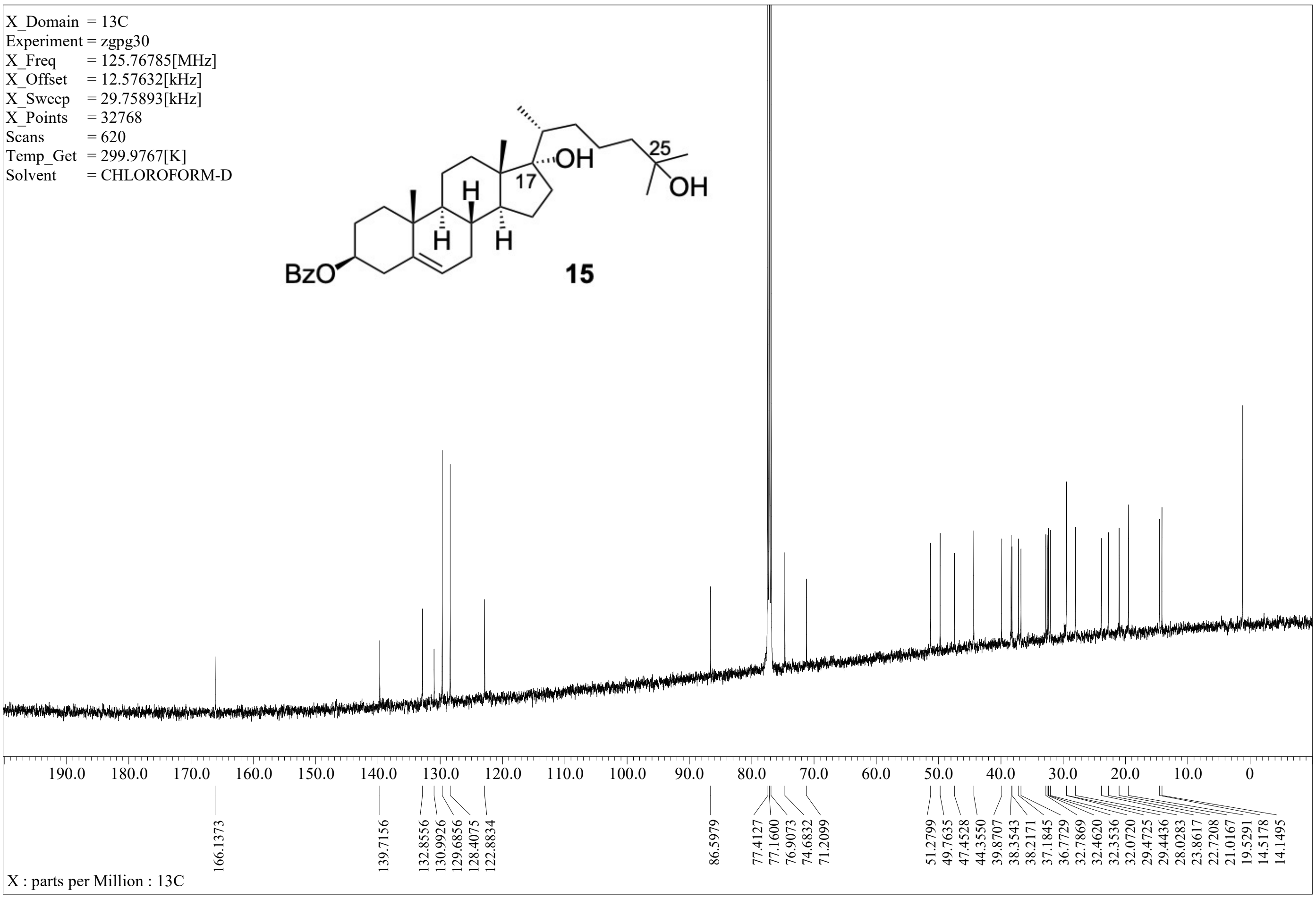
X_Domain = 13C
Experiment = zgpg30
X_Freq = 125.76785[MHz]
X_Offset = 12.57632[kHz]
X_Sweep = 29.75893[kHz]
X_Points = 32768
Scans = 620
Temp_Get = 299.9767[K]
Solvent = CHLOROFORM-D
BzO
OH
OH
17
25
15
166.1373
139.7156
132.8556
130.9926
129.6856
128.4075
122.8834
86.5979
77.4127
77.1600
76.9073
74.6832
71.2099
51.2799
49.7635
47.4528
44.3550
39.8707
38.3543
38.2171
37.1845
36.7729
32.7869
32.4620
32.3536
32.0720
29.4725
29.4436
28.0283
23.8617
22.7208
21.0167
19.5291
14.5178
14.1495
X : parts per Million : 13C

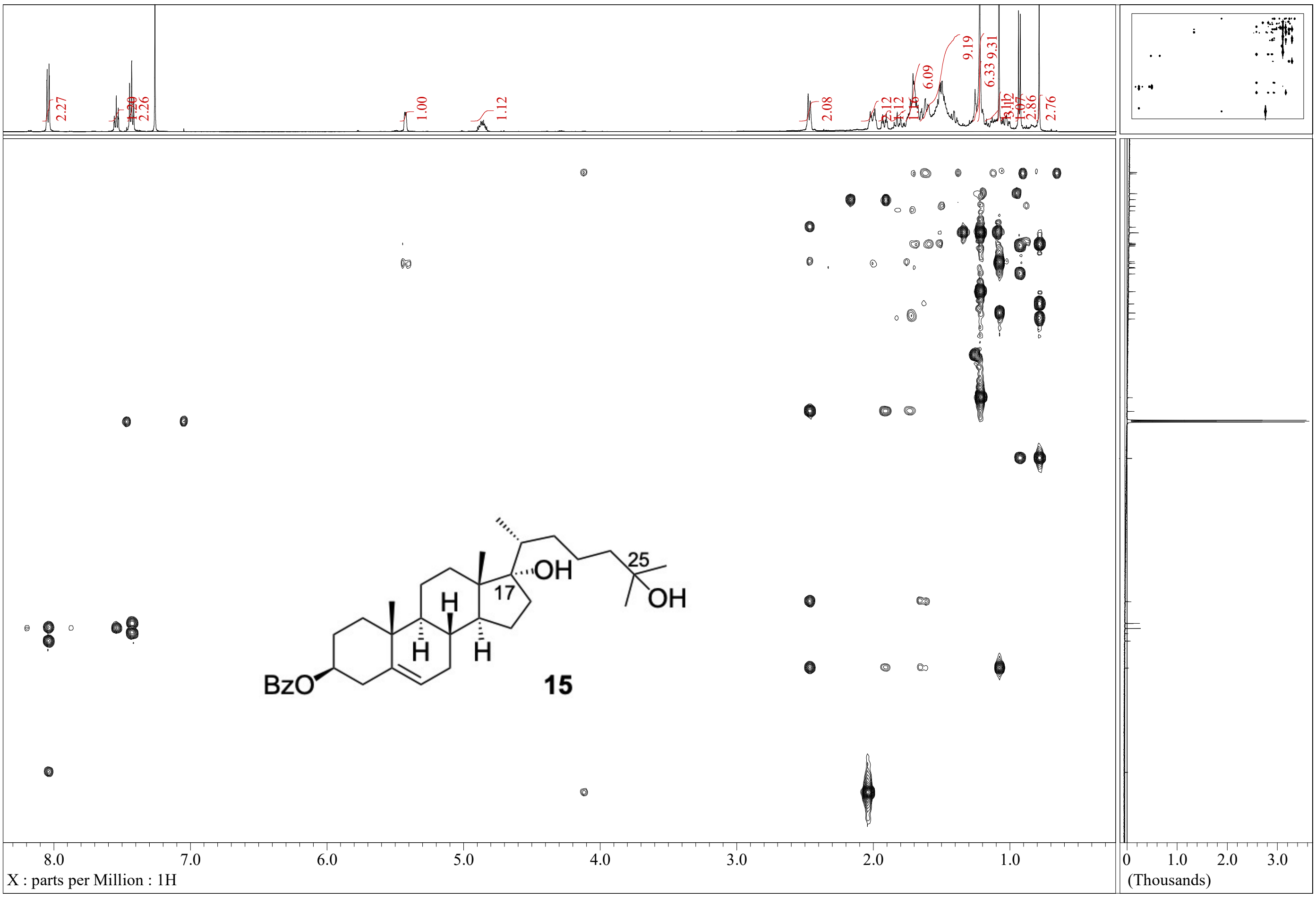
BzO
OH
OH
17
25
15
X : parts per Million : 1H
(Thousands)

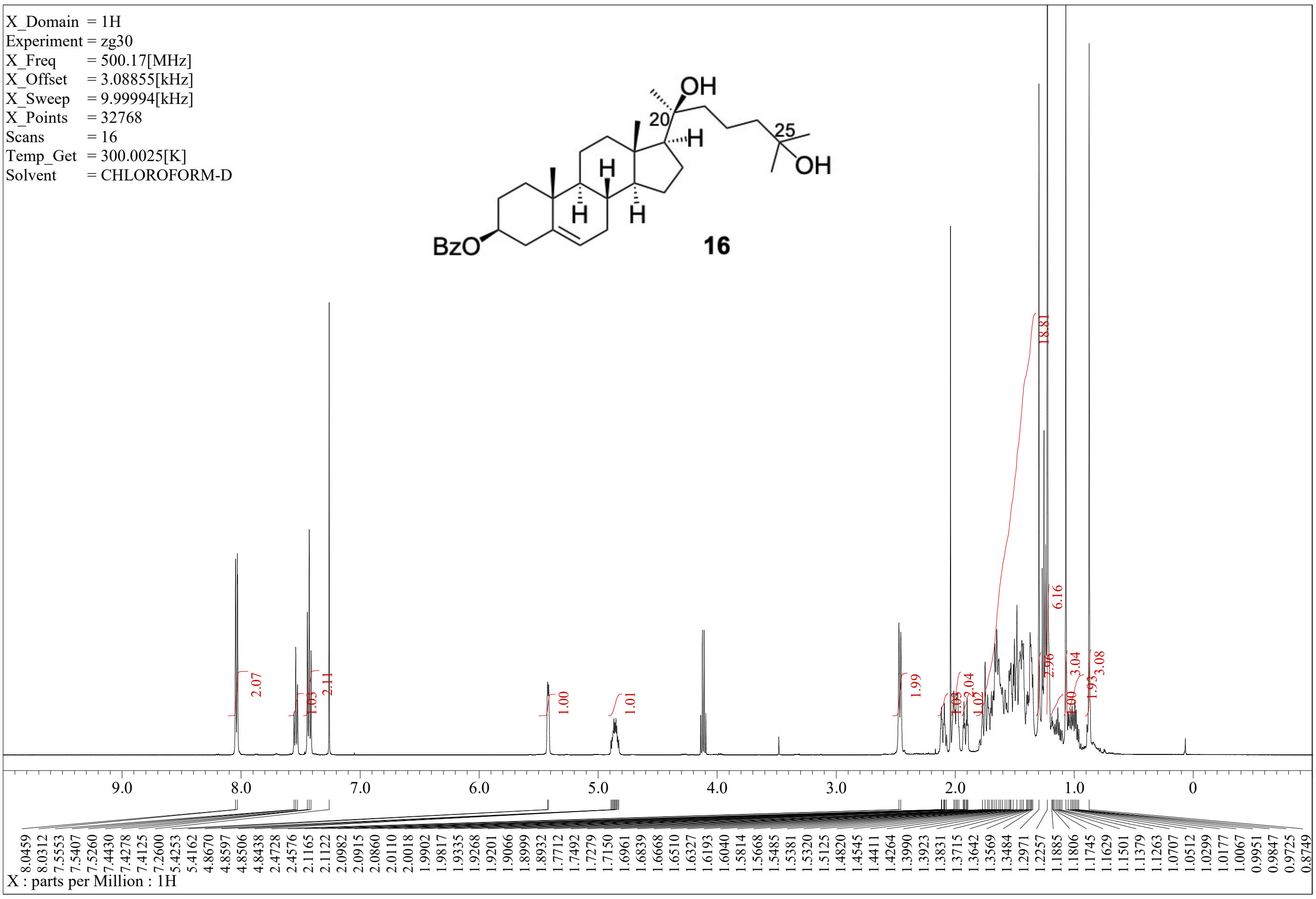
X_Domain = 1H
Experiment = zg30
X_Freq = 500.17[MHz]
X_Offset = 3.08855[kHz]
X_Sweep = 9.99994[kHz]
X_Points = 32768
Scans = 16
Temp_Get = 300.0025[K]
Solvent = CHLOROFORM-D
BzO
OH
20
25
OH
16
8.0459 8.0312 7.5553 7.5407 7.5260 7.4430 7.4278 7.4125 7.2600 5.4253 5.4162 4.8670 4.8597 4.8506 4.8438 2.4728 2.4576 2.1165 2.1122 2.0982 2.0915 2.0860 2.0110 2.0018 1.9902 1.9817 1.9335 1.9268 1.9201 1.9066 1.8999 1.8932 1.7712 1.7492 1.7279 1.7150 1.6961 1.6839 1.6668 1.6510 1.6327 1.6193 1.6040 1.5814 1.5668 1.5485 1.5381 1.5320 1.5125 1.4820 1.4545 1.4411 1.4264 1.3990 1.3923 1.3831 1.3715 1.3642 1.3569 1.3484 1.2971 1.2257 1.1885 1.1806 1.1745 1.1629 1.1501 1.1379 1.1263 1.0707 1.0512 1.0299 1.0177 1.0067 0.9951 0.9847 0.9725 0.8749
X : parts per Million : 1H

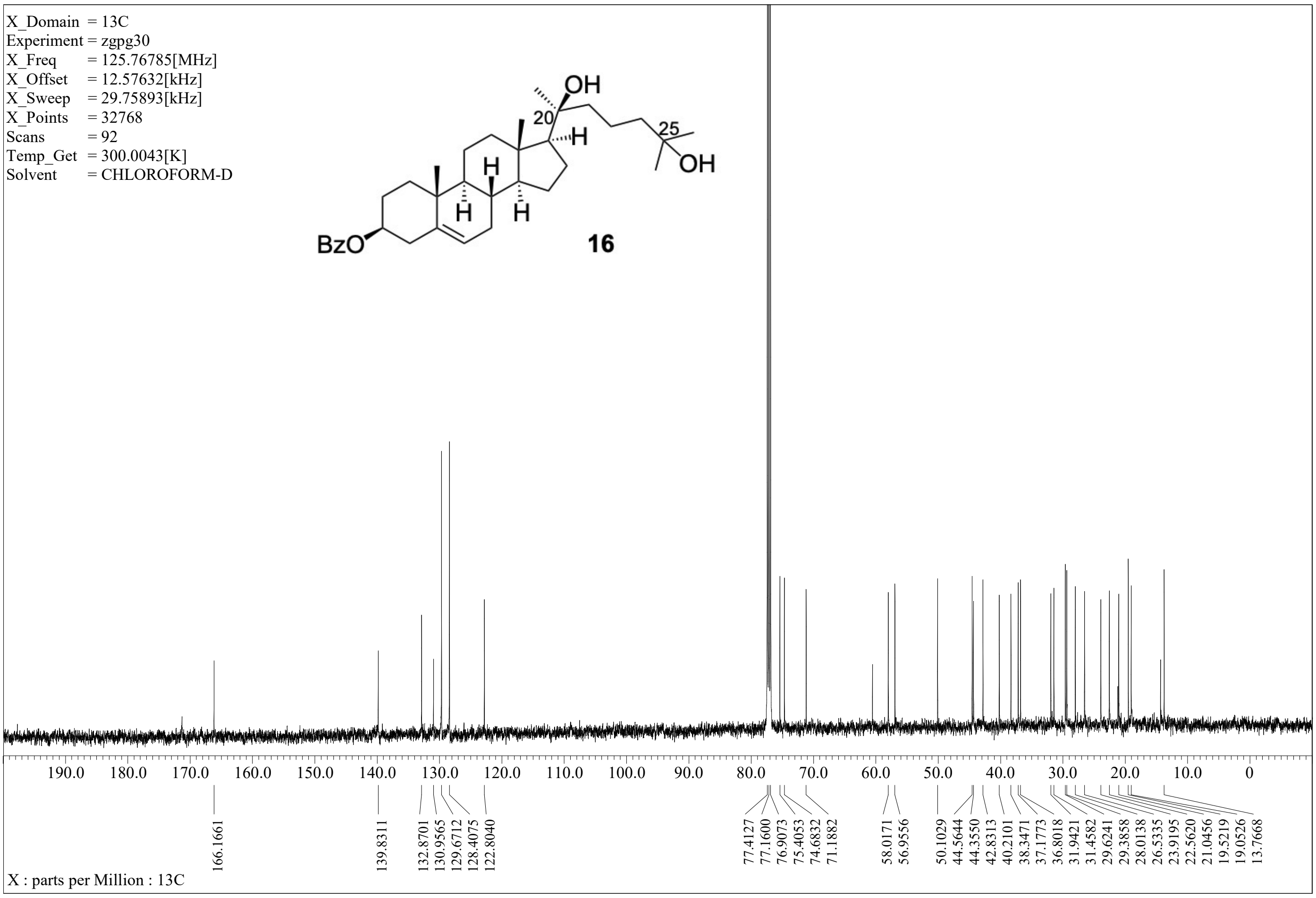
X_Domain = 13C
Experiment = zgpg30
X_Freq = 125.76785[MHz]
X_Offset = 12.57632[kHz]
X_Sweep = 29.75893[kHz]
X_Points = 32768
Scans = 92
Temp_Get = 300.0043[K]
Solvent = CHLOROFORM-D
BzO
OH
20
25
OH
16
166.1661 139.8311 132.8701 130.9565 129.6712 128.4075 122.8040 77.4127 77.1600 76.9073 75.4053 74.6832 71.1882 58.0171 56.9556 50.1029 44.5644 44.3550 42.8313 40.2101 38.3471 37.1773 36.8018 31.9421 31.4582 29.6241 29.3858 28.0138 26.5335 23.9195 22.5620 21.0456 19.5219 19.0526 13.7668
X : parts per Million : 13C

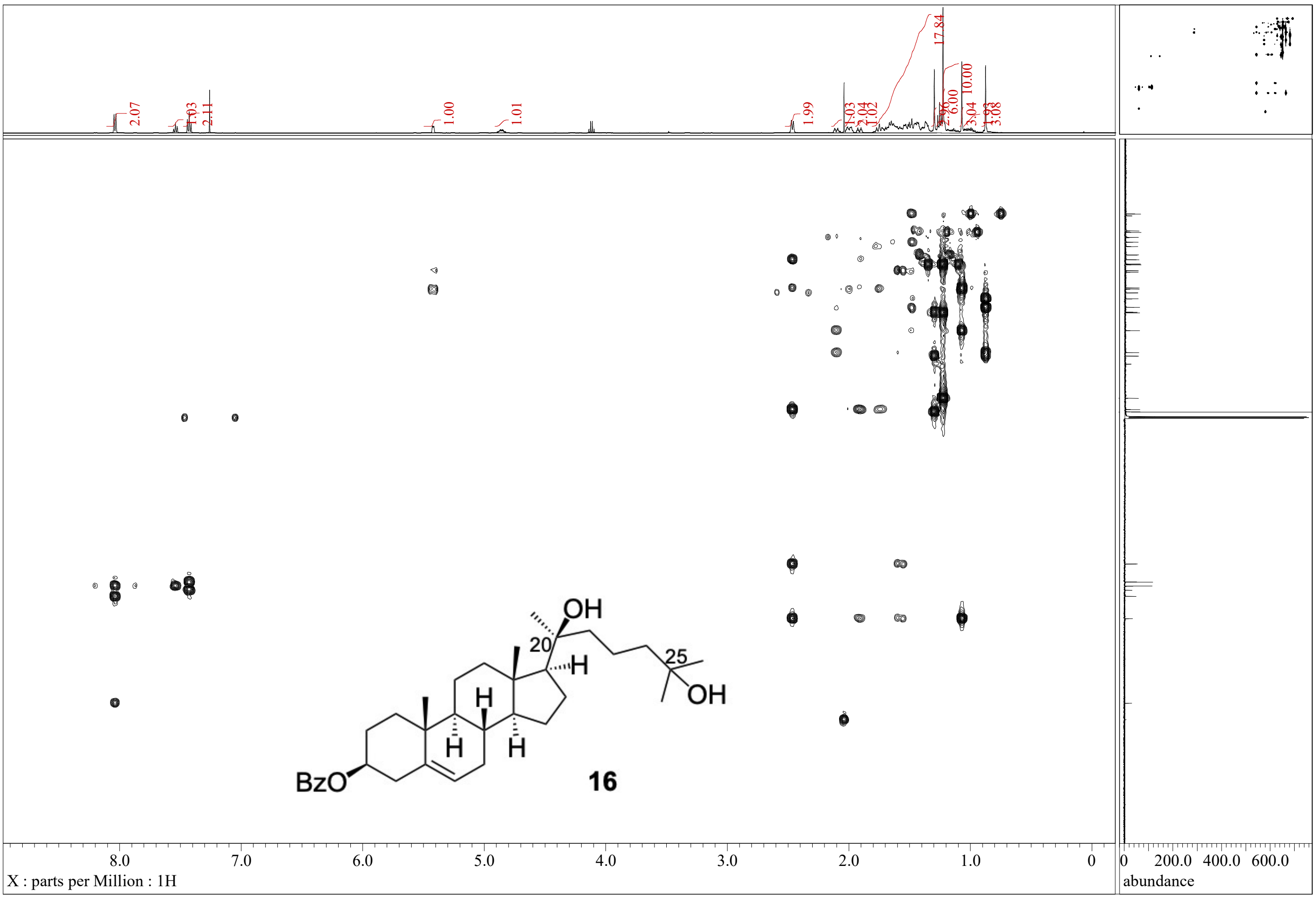

2.07
1.03
2.11
1.00
1.01
1.99
17.84
10.00
6.00
8.0
7.0
6.0
5.0
4.0
3.0
2.0
1.0
0
X : parts per Million : 1H
0
200.0
400.0
600.0
abundance
OH
20
H
25
OH
H
H
H
BzO
16

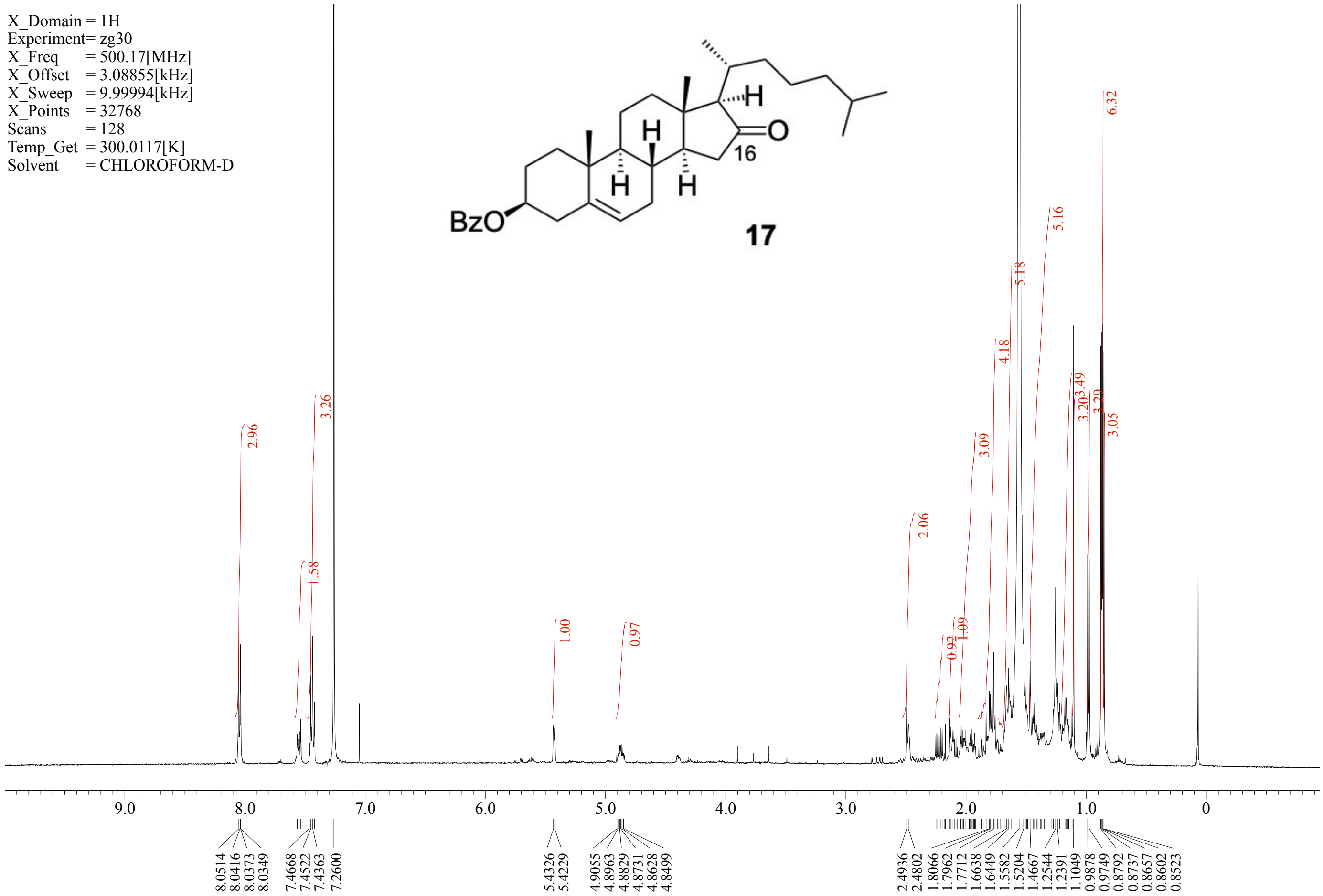

X_Domain = 1H
Experiment= zg30
X_Freq = 500.17[MHz]
X_Offset = 3.08855[kHz]
X_Sweep = 9.99994[kHz]
X_Points = 32768
Scans = 128
Temp_Get = 300.0117[K]
Solvent = CHLOROFORM-D
H
O
16
H
H
H
BzO
17
2.96
1.58
3.26
1.00
0.97
2.06
0.92
1.09
3.09
4.18
5.18
5.16
3.20
3.49
3.29
3.05
6.32
9.0
8.0
7.0
6.0
5.0
4.0
3.0
2.0
1.0
0
8.0514
8.0416
8.0373
8.0349
7.4668
7.4522
7.4363
7.2600
5.4326
5.4229
4.9055
4.8963
4.8829
4.8731
4.8628
4.8499
2.4936
2.4802
1.8066
1.7962
1.7712
1.6638
1.6449
1.5582
1.5204
1.4667
1.2544
1.2391
1.1049
0.9878
0.9749
0.8792
0.8737
0.8657
0.8602
0.8523

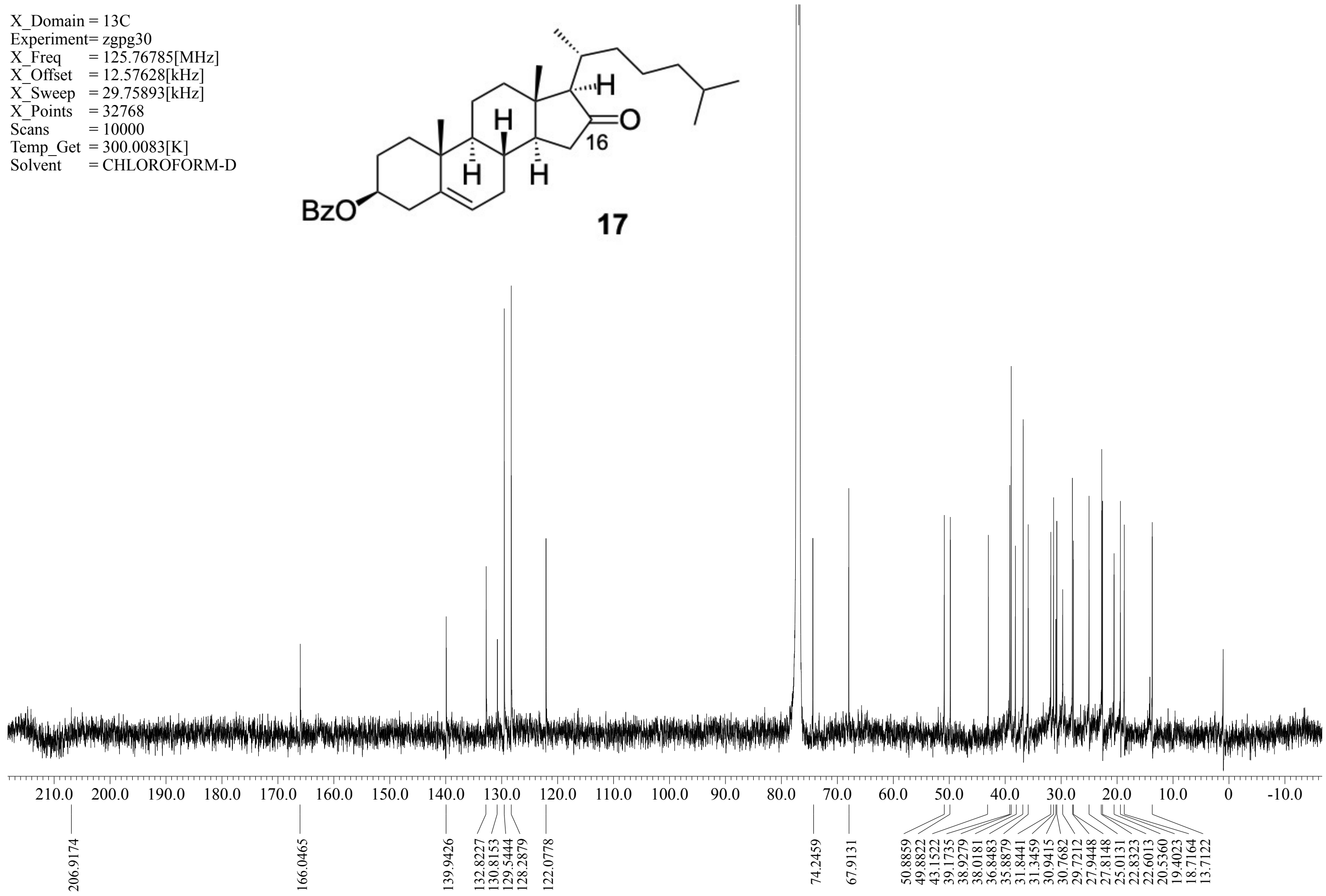
X_Domain = 13C
Experiment= zgpg30
X_Freq = 125.76785[MHz]
X_Offset = 12.57628[kHz]
X_Sweep = 29.75893[kHz]
X_Points = 32768
Scans = 10000
Temp_Get = 300.0083[K]
Solvent = CHLOROFORM-D
BzO
16
17
206.9174
166.0465
139.9426
132.8227
130.8153
129.5444
128.2879
122.0778
74.2459
67.9131
50.8859
49.8822
43.1522
39.1735
38.9279
38.0181
36.8483
35.8879
31.8441
31.3459
30.9415
30.7682
29.7212
27.9448
27.8148
25.0131
22.8323
22.6013
20.5360
19.4023
18.7164
13.7122

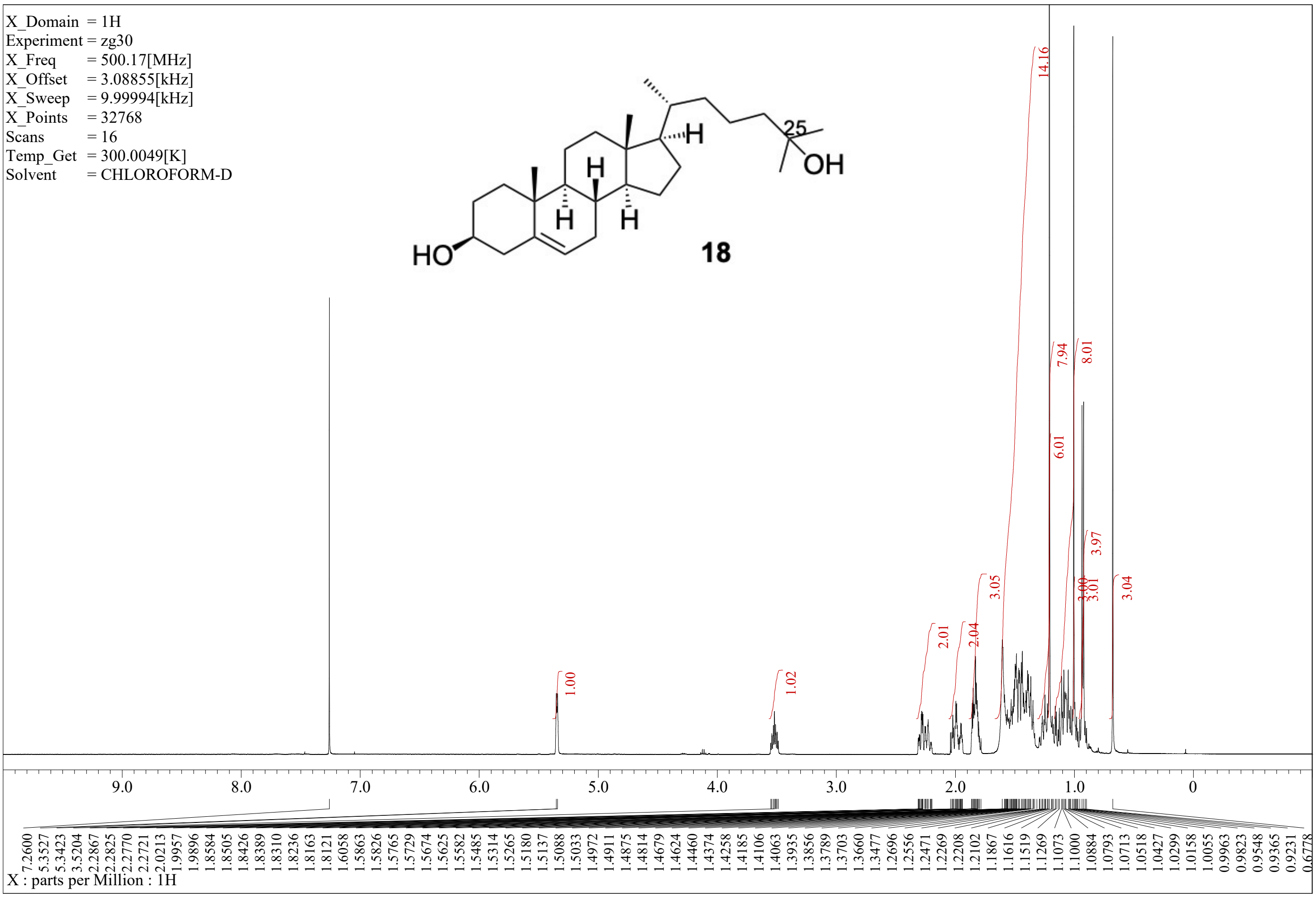
X_Domain = 1H
Experiment = zg30
X_Freq = 500.17[MHz]
X_Offset = 3.08855[kHz]
X_Sweep = 9.99994[kHz]
X_Points = 32768
Scans = 16
Temp_Get = 300.0049[K]
Solvent = CHLOROFORM-D
25
OH
HO
18
1.00
1.02
2.01
2.04
3.05
14.16
7.94
6.01
8.01
3.97
3.00
3.01
3.04
X : parts per Million : 1H
7.2600
5.3527
5.3423
3.5204
2.2867
2.2825
2.2770
2.2721
2.0213
1.9957
1.9896
1.8584
1.8505
1.8426
1.8389
1.8310
1.8236
1.8163
1.8121
1.6058
1.5863
1.5826
1.5765
1.5729
1.5674
1.5625
1.5582
1.5485
1.5314
1.5265
1.5180
1.5137
1.5088
1.5033
1.4972
1.4911
1.4875
1.4814
1.4679
1.4624
1.4460
1.4374
1.4258
1.4185
1.4106
1.4063
1.3935
1.3856
1.3789
1.3703
1.3660
1.3477
1.2696
1.2556
1.2471
1.2269
1.2208
1.2102
1.1867
1.1616
1.1519
1.1269
1.1073
1.1000
1.0884
1.0793
1.0713
1.0518
1.0427
1.0299
1.0158
1.0055
0.9963
0.9823
0.9548
0.9365
0.9231
0.6778

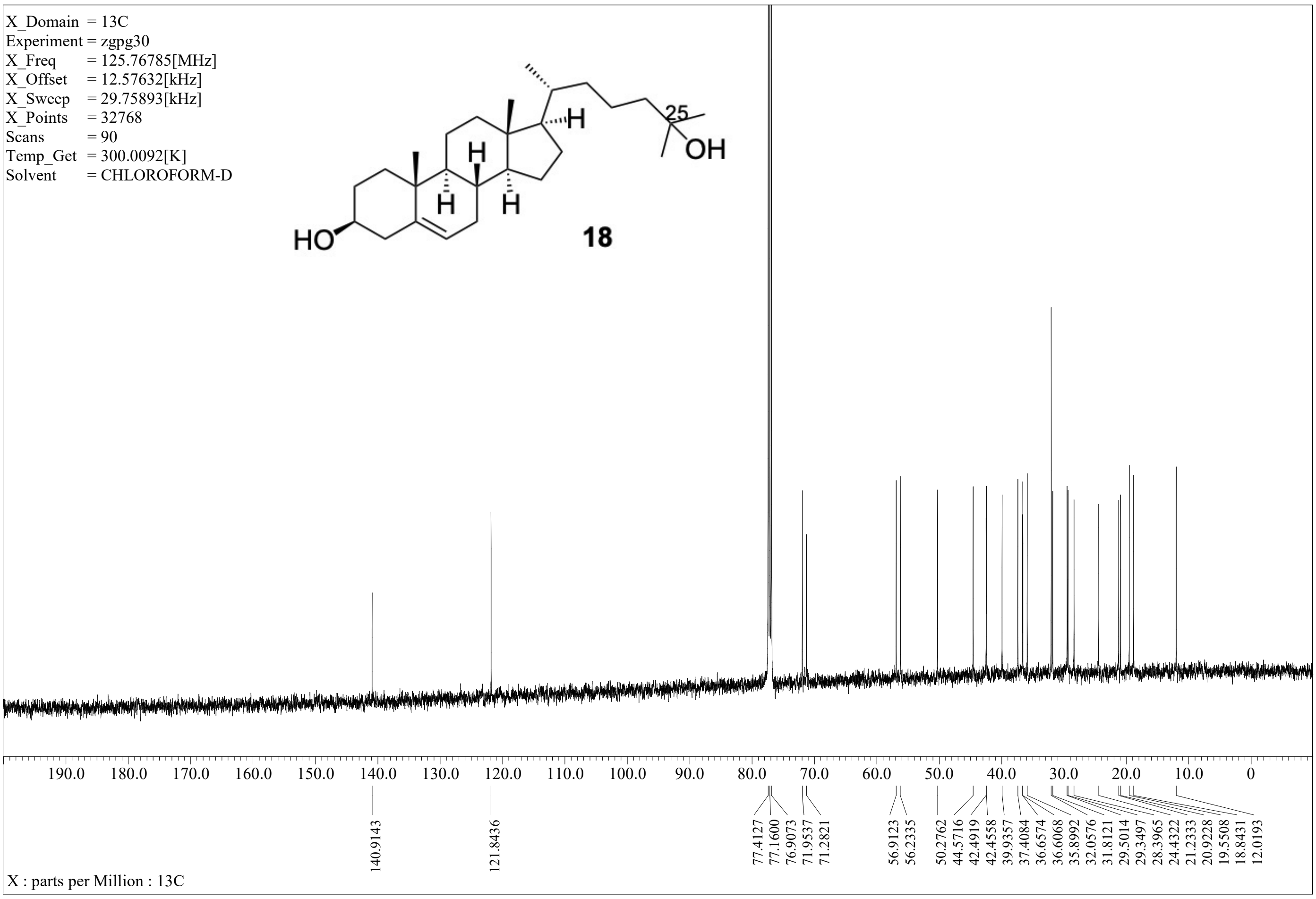
X_Domain = 13C
Experiment = zgpg30
X_Freq = 125.76785[MHz]
X_Offset = 12.57632[kHz]
X_Sweep = 29.75893[kHz]
X_Points = 32768
Scans = 90
Temp_Get = 300.0092[K]
Solvent = CHLOROFORM-D
25
OH
HO
18
140.9143
121.8436
77.4127
77.1600
76.9073
71.9537
71.2821
56.9123
56.2335
50.2762
44.5716
42.4919
42.4558
39.9357
37.4084
36.6574
36.6068
35.8992
32.0576
31.8121
29.5014
29.3497
28.3965
24.4322
21.2333
20.9228
19.5508
18.8431
12.0193
X : parts per Million : 13C

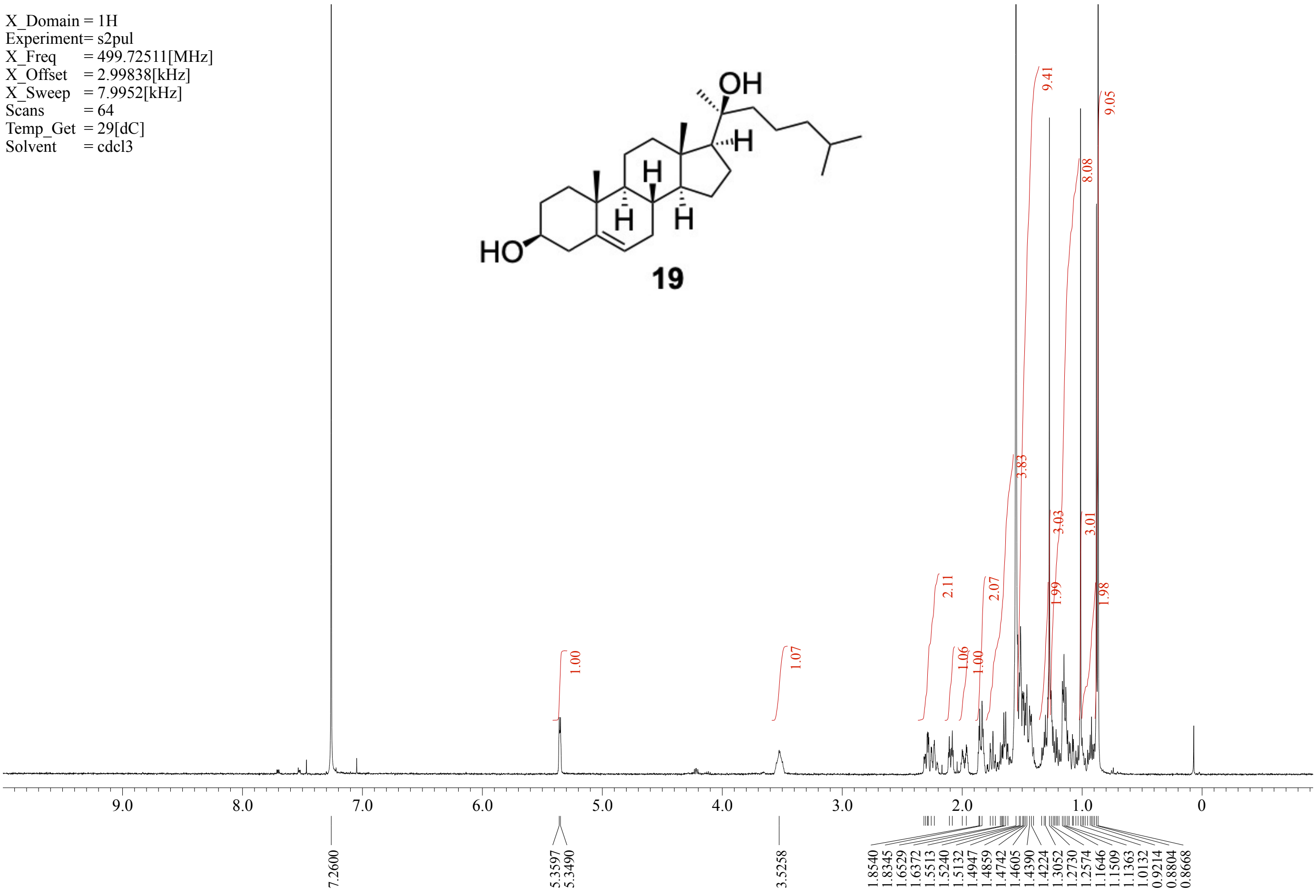
X_Domain = 1H
Experiment= s2pul
X_Freq = 499.72511[MHz]
X_Offset = 2.99838[kHz]
X_Sweep = 7.9952[kHz]
Scans = 64
Temp_Get = 29[dC]
Solvent = cdcl3
OH
HO
19
1.00
1.07
2.11
1.06
1.00
2.07
3.83
9.41
1.99
3.03
8.08
3.01
1.98
9.05
7.2600
5.3597
5.3490
3.5258
1.8540
1.8345
1.6529
1.6372
1.5513
1.5240
1.5132
1.4947
1.4859
1.4742
1.4605
1.4390
1.4224
1.3052
1.2730
1.2574
1.1646
1.1509
1.1363
1.0132
0.9214
0.8804
0.8668

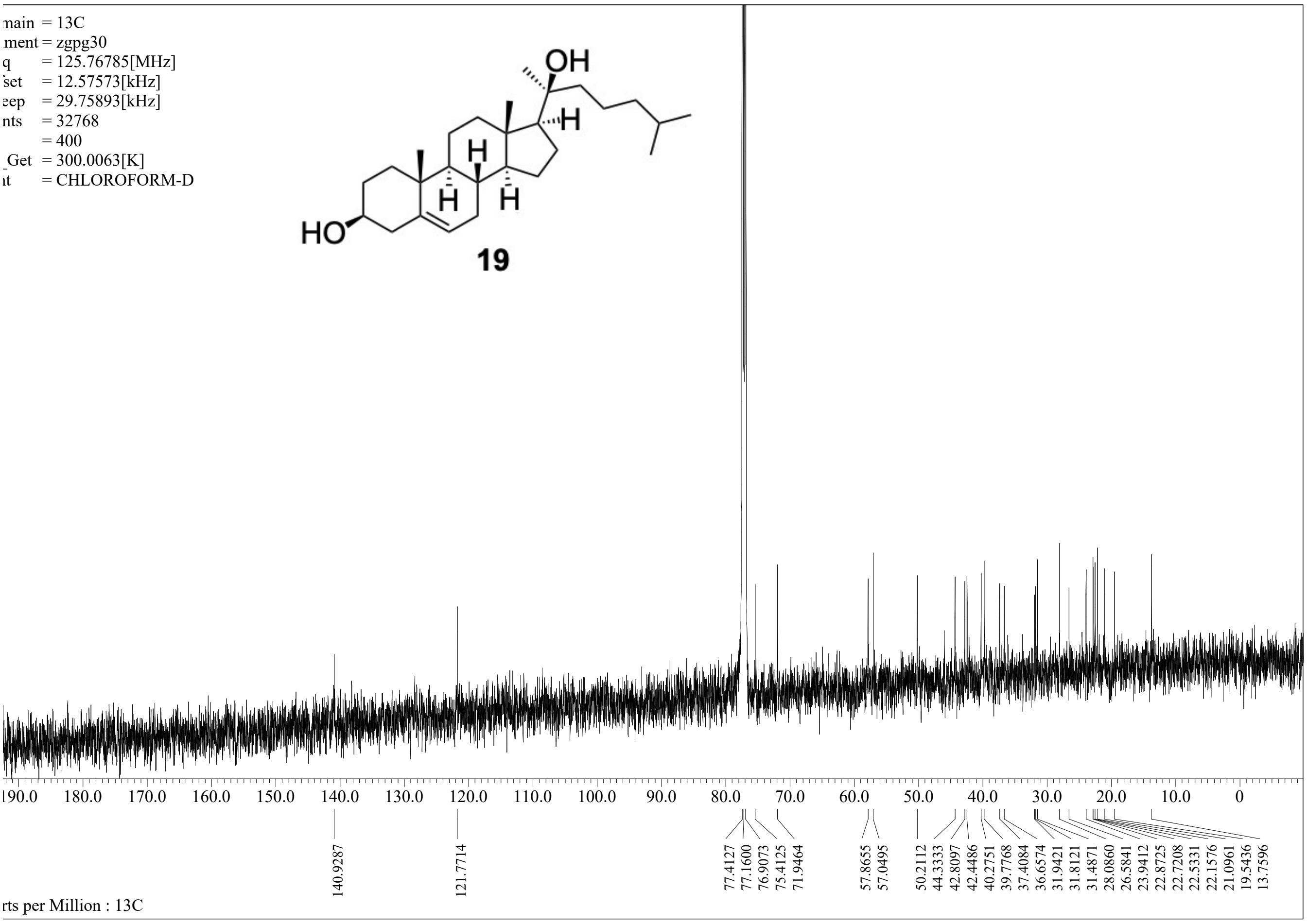
main = 13C
ment = zgpg30
q = 125.76785[MHz]
set = 12.57573[kHz]
eep = 29.75893[kHz]
nts = 32768
= 400
_Get = 300.0063[K]
t = CHLOROFORM-D
OH
H
H
H
H
HO
19
190.0 180.0 170.0 160.0 150.0 140.0 130.0 120.0 110.0 100.0 90.0 80.0 70.0 60.0 50.0 40.0 30.0 20.0 10.0 0
140.9287
121.7714
77.4127
77.1600
76.9073
75.4125
71.9464
57.8655
57.0495
50.2112
44.3333
42.8097
42.4486
40.2751
39.7768
37.4084
36.6574
31.9421
31.8121
31.4871
28.0860
26.5841
23.9412
22.8725
22.7208
22.5331
22.1576
21.0961
19.5436
13.7596
rts per Million : 13C

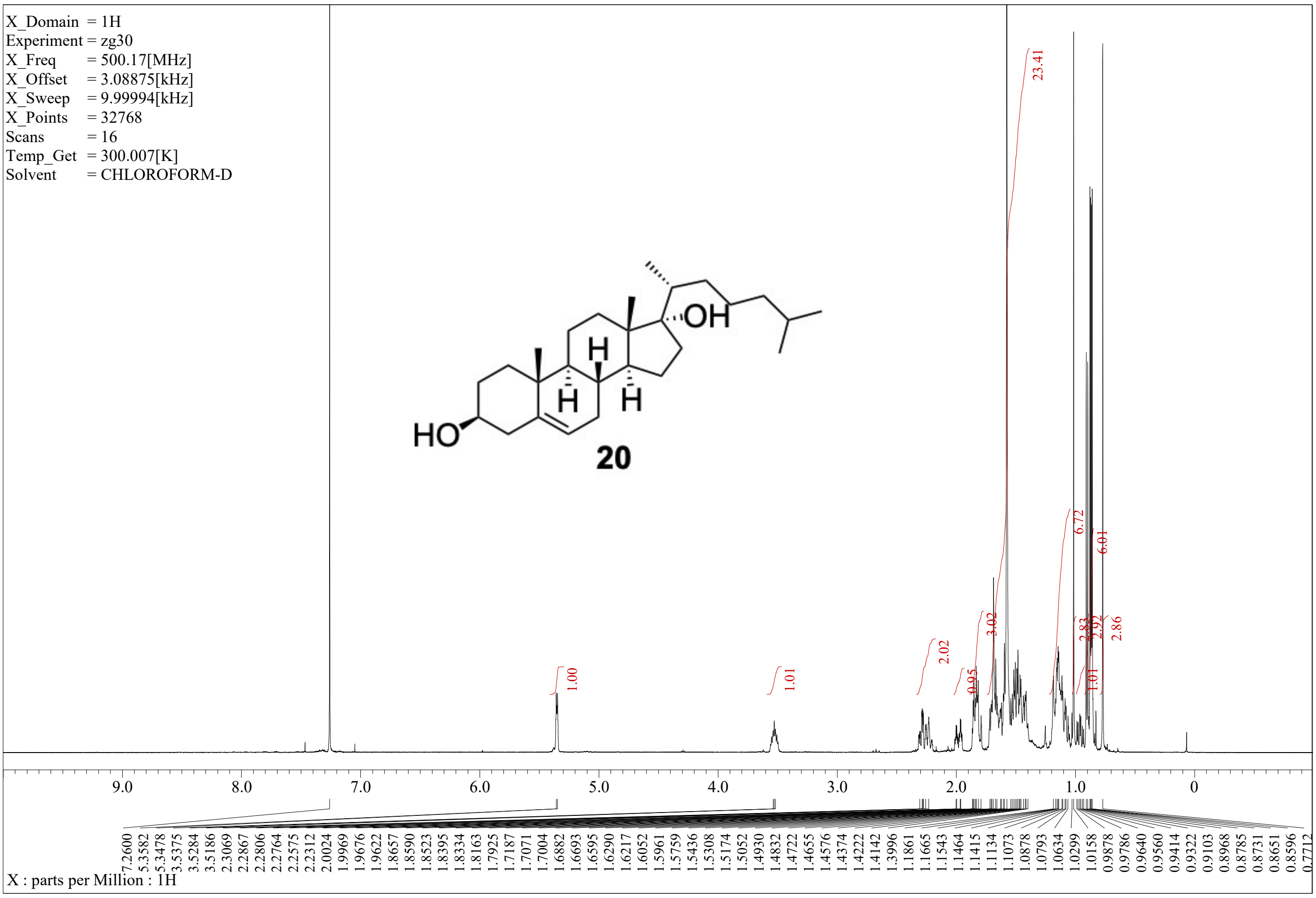
X_Domain = 1H
Experiment = zg30
X_Freq = 500.17[MHz]
X_Offset = 3.08875[kHz]
X_Sweep = 9.99994[kHz]
X_Points = 32768
Scans = 16
Temp_Get = 300.007[K]
Solvent = CHLOROFORM-D
OH
H
H
H
HO
20
23.41
6.72
6.01
3.02
2.02
0.95
2.83
2.92
2.86
1.01
1.00
1.01
9.0 8.0 7.0 6.0 5.0 4.0 3.0 2.0 1.0 0
7.2600
5.3582
5.3478
3.5375
3.5284
3.5186
2.3069
2.2867
2.2806
2.2764
2.2575
2.2312
2.0024
1.9969
1.9676
1.9622
1.8657
1.8590
1.8523
1.8395
1.8334
1.8163
1.7925
1.7187
1.7071
1.7004
1.6882
1.6693
1.6595
1.6290
1.6217
1.6052
1.5961
1.5759
1.5436
1.5308
1.5174
1.5052
1.4930
1.4832
1.4722
1.4655
1.4576
1.4374
1.4222
1.4142
1.3996
1.1861
1.1665
1.1543
1.1464
1.1415
1.1134
1.1073
1.0878
1.0793
1.0634
1.0299
1.0158
0.9878
0.9786
0.9640
0.9560
0.9414
0.9322
0.9103
0.8968
0.8785
0.8731
0.8651
0.8596
0.7712
X : parts per Million : 1H

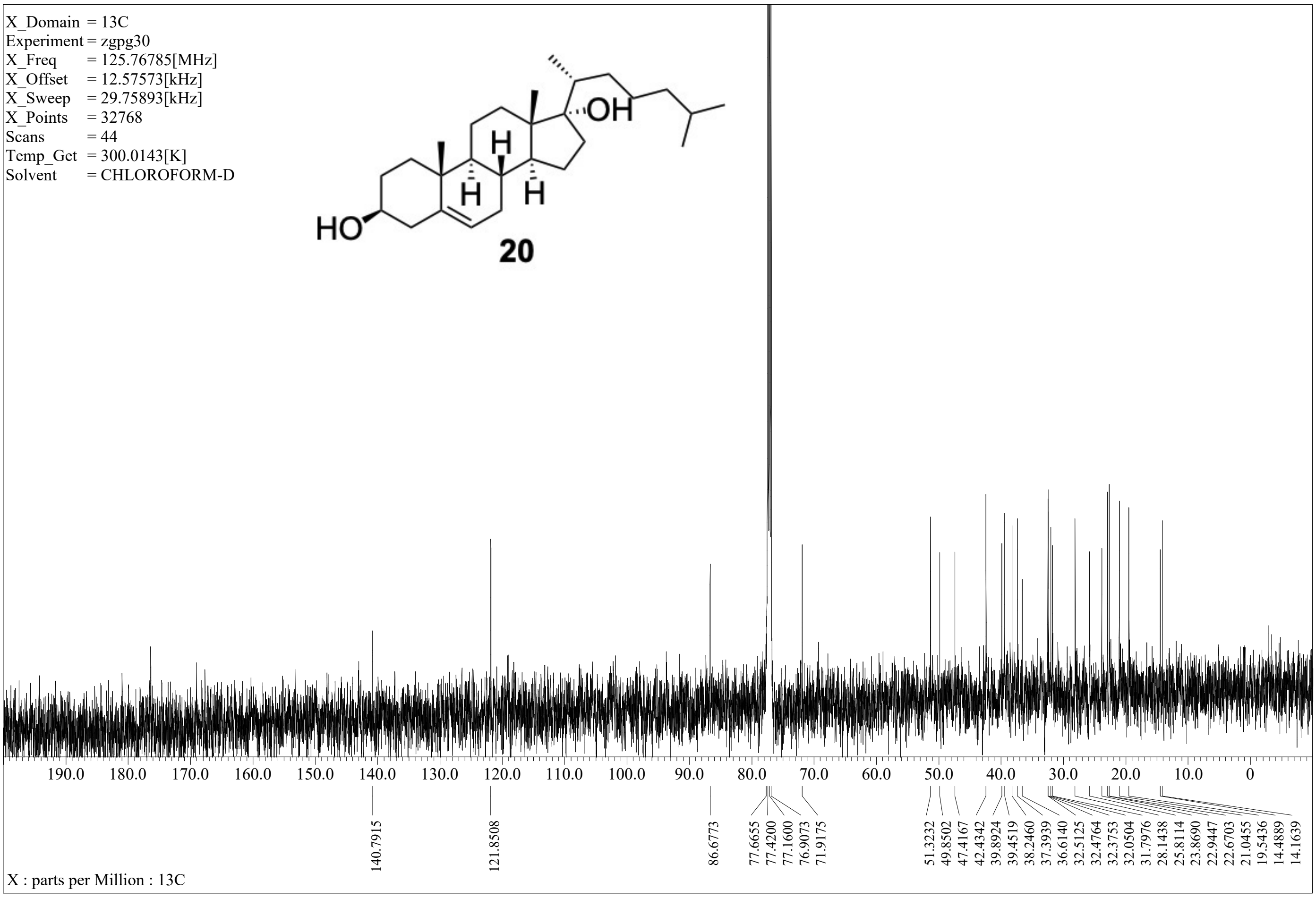

X_Domain = 13C
Experiment = zgpg30
X_Freq = 125.76785[MHz]
X_Offset = 12.57573[kHz]
X_Sweep = 29.75893[kHz]
X_Points = 32768
Scans = 44
Temp_Get = 300.0143[K]
Solvent = CHLOROFORM-D
HO
OH
H
H
H
20
190.0 180.0 170.0 160.0 150.0 140.0 130.0 120.0 110.0 100.0 90.0 80.0 70.0 60.0 50.0 40.0 30.0 20.0 10.0 0
140.7915
121.8508
86.6773
77.6655
77.4200
77.1600
76.9073
71.9175
51.3232
49.8502
47.4167
42.4342
39.8924
39.4519
38.2460
37.3939
36.6140
32.5125
32.4764
32.3753
32.0504
31.7976
28.1438
25.8114
23.8690
22.9447
22.6703
21.0455
19.5436
14.4889
14.1639
X : parts per Million : 13C

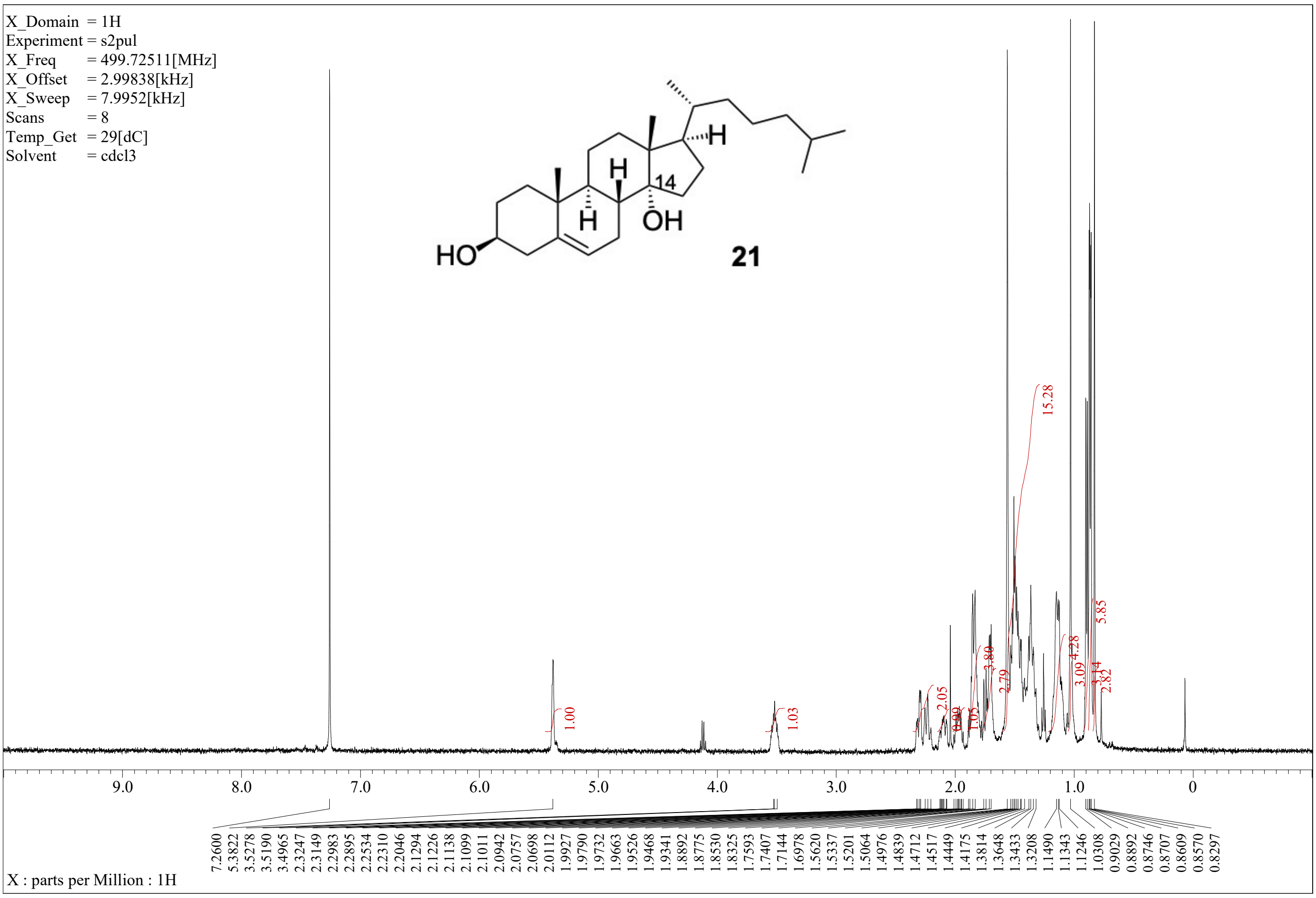

X_Domain = 1H
Experiment = s2pul
X_Freq = 499.72511[MHz]
X_Offset = 2.99838[kHz]
X_Sweep = 7.9952[kHz]
Scans = 8
Temp_Get = 29[dC]
Solvent = cdcl3
HO
H
H
H
14
OH
21
1.00
1.03
2.05
1.05
3.80
2.79
15.28
3.09
4.28
3.14
2.82
5.85
9.0 8.0 7.0 6.0 5.0 4.0 3.0 2.0 1.0 0
7.2600
5.3822
3.5278
3.5190
3.4965
2.3247
2.3149
2.2983
2.2895
2.2534
2.2310
2.2046
2.1294
2.1226
2.1138
2.1099
2.1011
2.0942
2.0757
2.0698
2.0112
1.9927
1.9790
1.9732
1.9663
1.9526
1.9468
1.9341
1.8892
1.8775
1.8530
1.8325
1.7593
1.7407
1.7144
1.6978
1.5620
1.5337
1.5201
1.5064
1.4976
1.4839
1.4712
1.4517
1.4449
1.4175
1.3814
1.3648
1.3433
1.3208
1.1490
1.1343
1.1246
1.0308
0.9029
0.8892
0.8746
0.8707
0.8609
0.8570
0.8297
X : parts per Million : 1H

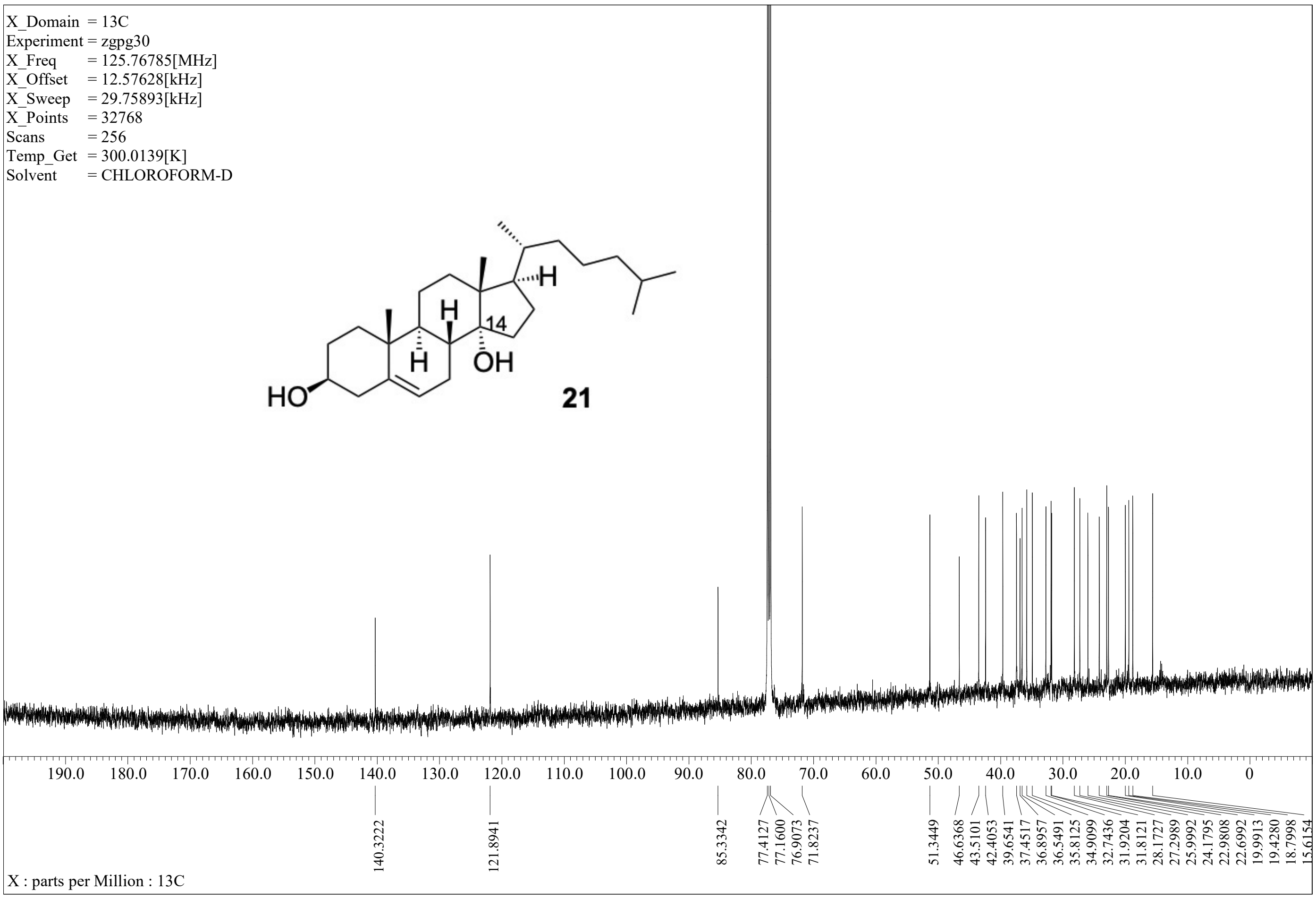
X_Domain = 13C
Experiment = zgpg30
X_Freq = 125.76785[MHz]
X_Offset = 12.57628[kHz]
X_Sweep = 29.75893[kHz]
X_Points = 32768
Scans = 256
Temp_Get = 300.0139[K]
Solvent = CHLOROFORM-D
HO
H
H
H
14
OH
21
140.3222
121.8941
85.3342
77.4127
77.1600
76.9073
71.8237
51.3449
46.6368
43.5101
42.4053
39.6541
37.4517
36.8957
36.5491
35.8125
34.9099
32.7436
31.9204
31.8121
28.1727
27.2989
25.9992
24.1795
22.9808
22.6992
19.9913
19.4280
18.7998
15.6154
X : parts per Million : 13C

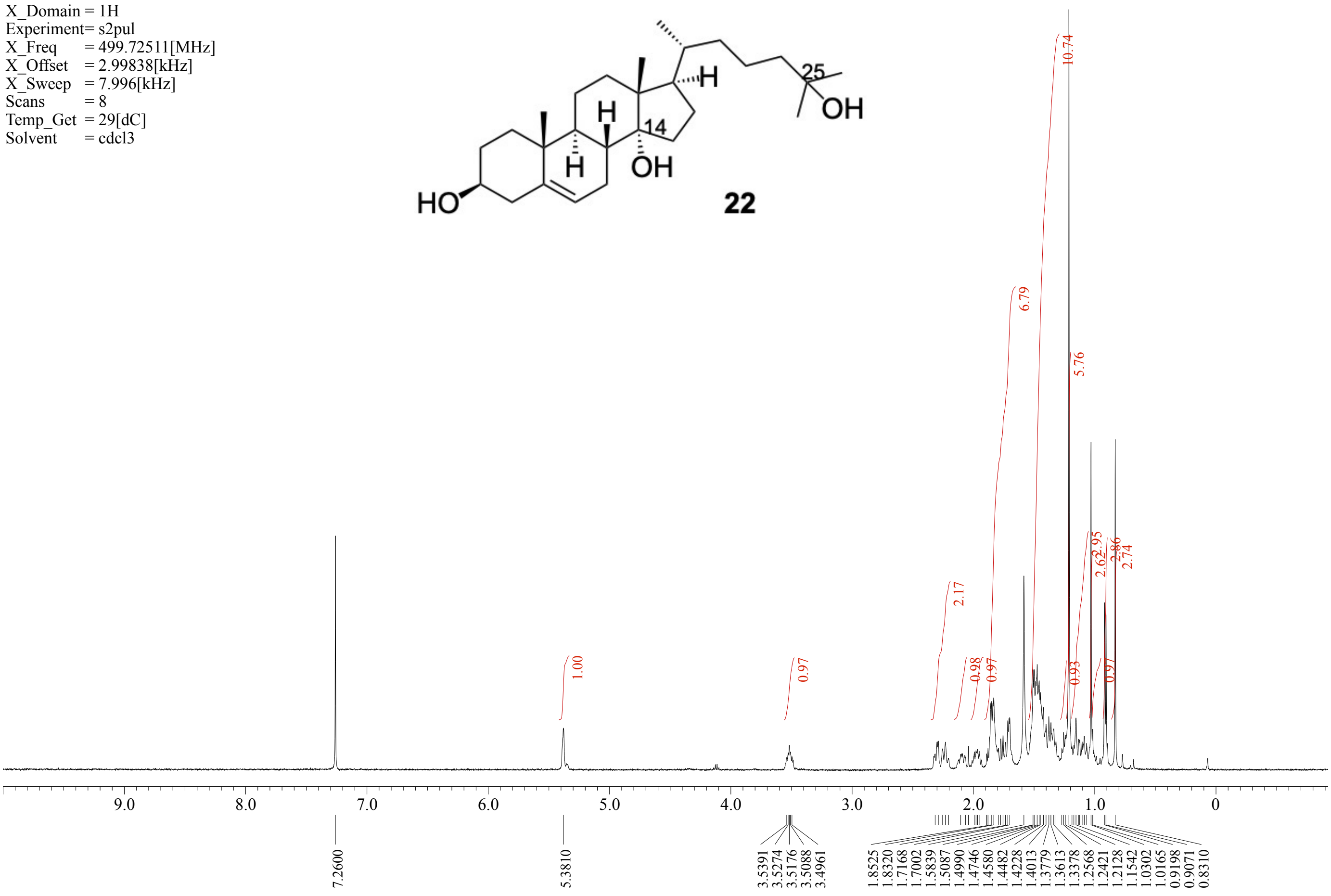
X_Domain = 1H
Experiment= s2pul
X_Freq = 499.72511[MHz]
X_Offset = 2.99838[kHz]
X_Sweep = 7.996[kHz]
Scans = 8
Temp_Get = 29[dC]
Solvent = cdcl3
HO
H
H
H
14
OH
25
OH
22
7.2600
5.3810
3.5391
3.5274
3.5176
3.5088
3.4961
1.8525
1.8320
1.7168
1.7002
1.5839
1.5087
1.4990
1.4746
1.4580
1.4482
1.4228
1.4013
1.3779
1.3613
1.3378
1.2568
1.2421
1.2128
1.1542
1.0302
1.0165
0.9198
0.9071
0.8310

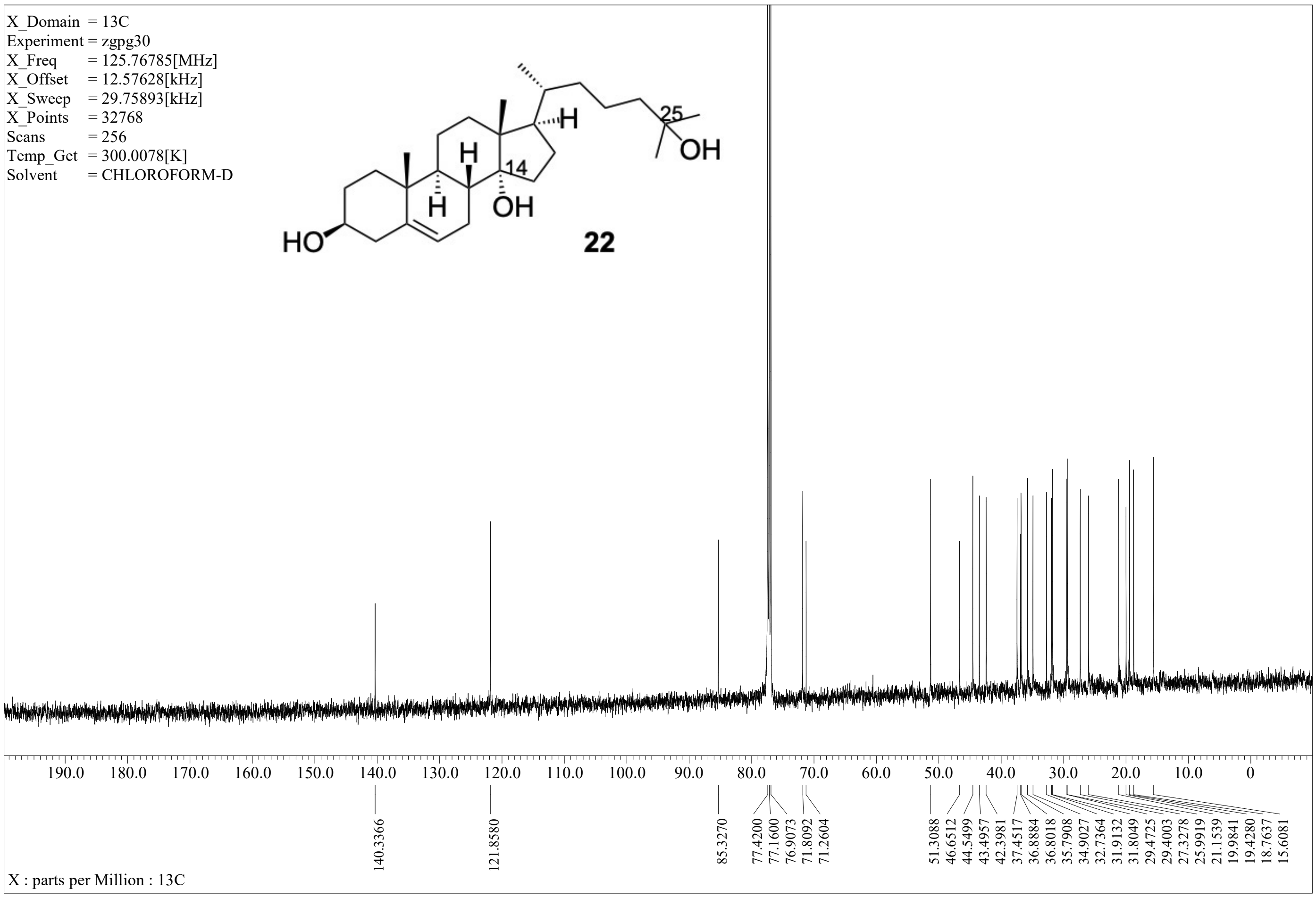
X_Domain = 13C
Experiment = zgpg30
X_Freq = 125.76785[MHz]
X_Offset = 12.57628[kHz]
X_Sweep = 29.75893[kHz]
X_Points = 32768
Scans = 256
Temp_Get = 300.0078[K]
Solvent = CHLOROFORM-D
HO
H
H
OH
14
H
25
OH
22
190.0 180.0 170.0 160.0 150.0 140.0 130.0 120.0 110.0 100.0 90.0 80.0 70.0 60.0 50.0 40.0 30.0 20.0 10.0 0
140.3366
121.8580
85.3270
77.4200
77.1600
76.9073
71.8092
71.2604
51.3088
46.6512
44.5499
43.4957
42.3981
37.4517
36.8884
36.8018
35.7908
34.9027
32.7364
31.9132
31.8049
29.4725
29.4003
27.3278
25.9919
21.1539
19.9841
19.4280
18.7637
15.6081
X : parts per Million : 13C

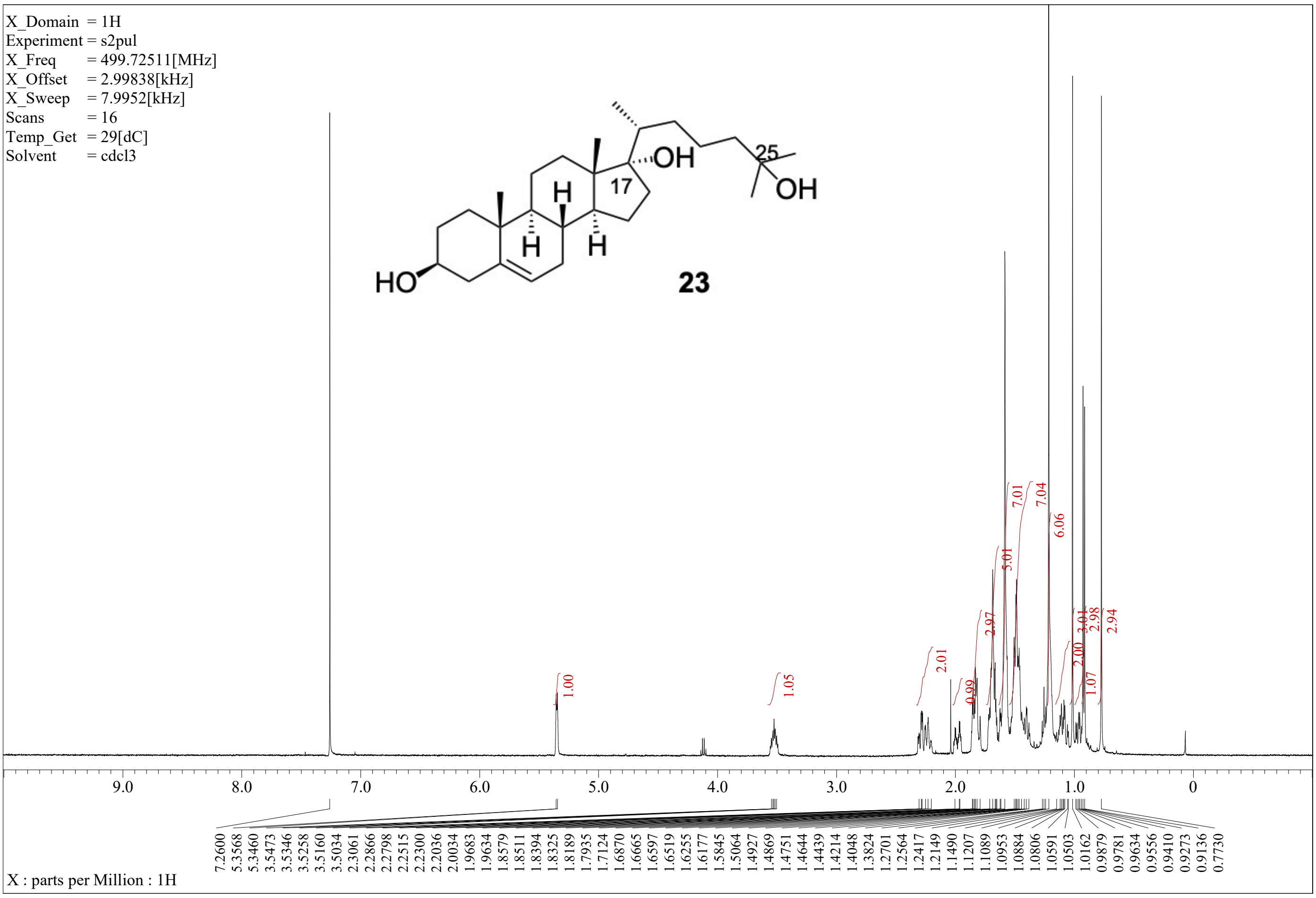
X_Domain = 1H
Experiment = s2pul
X_Freq = 499.72511[MHz]
X_Offset = 2.99838[kHz]
X_Sweep = 7.9952[kHz]
Scans = 16
Temp_Get = 29[dC]
Solvent = cdcl3
HO
H
H
H
17
OH
25
OH
23
1.00
1.05
2.01
0.99
2.97
5.01
7.01
7.04
6.06
2.00
1.07
3.01
2.98
2.94
9.0 8.0 7.0 6.0 5.0 4.0 3.0 2.0 1.0 0
7.2600
5.3568
5.3460
3.5473
3.5346
3.5258
3.5160
3.5034
2.3061
2.2866
2.2798
2.2515
2.2300
2.2036
2.0034
1.9683
1.9634
1.8579
1.8511
1.8394
1.8325
1.8189
1.7935
1.7124
1.6870
1.6665
1.6597
1.6519
1.6255
1.6177
1.5845
1.5064
1.4927
1.4869
1.4751
1.4644
1.4439
1.4214
1.4048
1.3824
1.2701
1.2564
1.2417
1.2149
1.1490
1.1207
1.1089
1.0953
1.0884
1.0806
1.0591
1.0503
1.0162
0.9879
0.9781
0.9634
0.9556
0.9410
0.9273
0.9136
0.7730
X : parts per Million : 1H

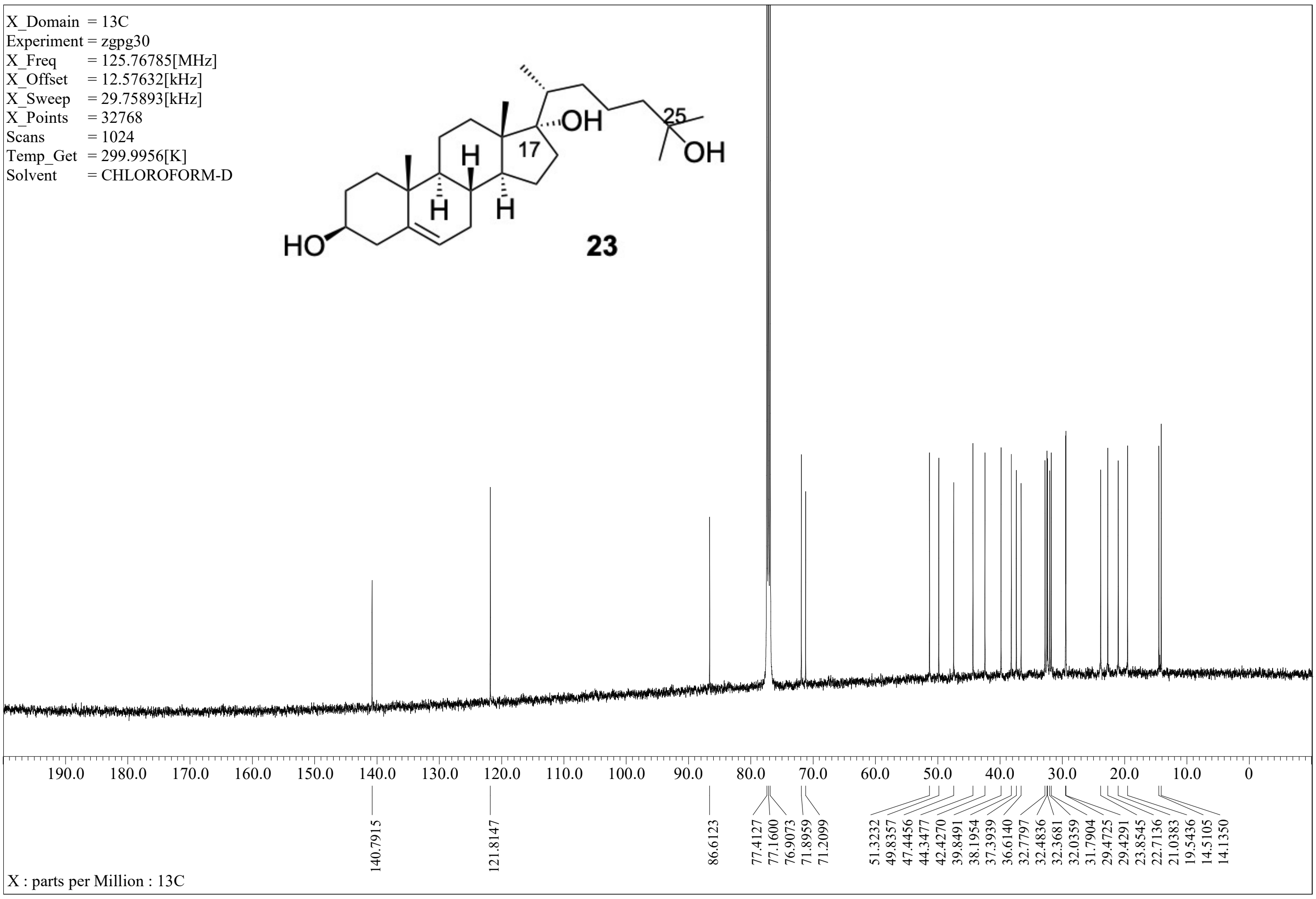
X_Domain = 13C
Experiment = zgpg30
X_Freq = 125.76785[MHz]
X_Offset = 12.57632[kHz]
X_Sweep = 29.75893[kHz]
X_Points = 32768
Scans = 1024
Temp_Get = 299.9956[K]
Solvent = CHLOROFORM-D
OH
25
17
OH
HO
23
190.0 180.0 170.0 160.0 150.0 140.0 130.0 120.0 110.0 100.0 90.0 80.0 70.0 60.0 50.0 40.0 30.0 20.0 10.0 0
140.7915
121.8147
86.6123
77.4127
77.1600
76.9073
71.8959
71.2099
51.3232
49.8357
47.4456
44.3477
42.4270
39.8491
38.1954
37.3939
36.6140
32.7797
32.4836
32.3681
32.0359
31.7904
29.4725
29.4291
23.8545
22.7136
21.0383
19.5436
14.5105
14.1350
X : parts per Million : 13C

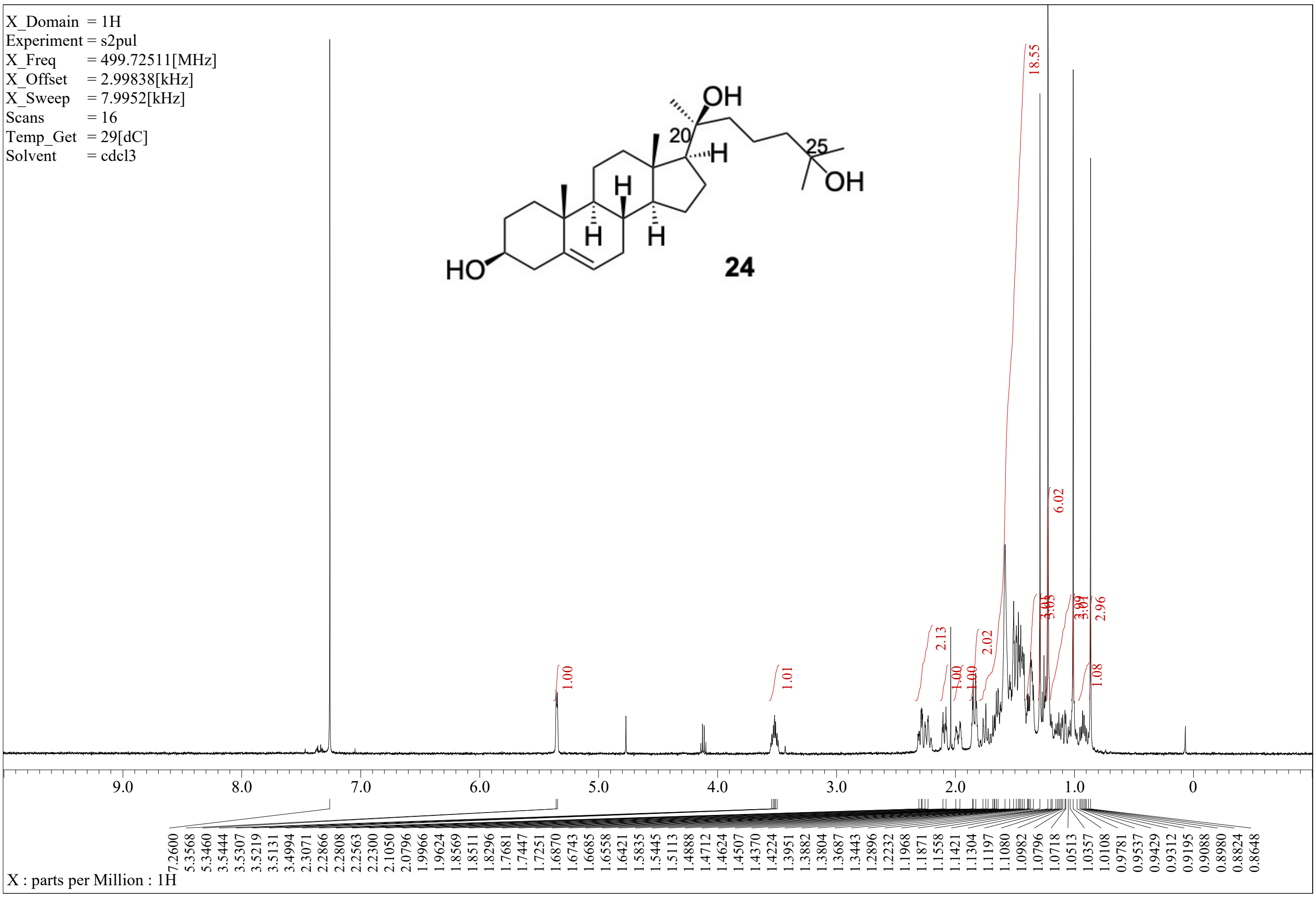
X_Domain = 1H
Experiment = s2pul
X_Freq = 499.72511[MHz]
X_Offset = 2.99838[kHz]
X_Sweep = 7.9952[kHz]
Scans = 16
Temp_Get = 29[dC]
Solvent = cdcl3
OH
20
25
OH
HO
24
18.55
6.02
2.96
2.13
2.02
1.00
1.01
1.08
9.0 8.0 7.0 6.0 5.0 4.0 3.0 2.0 1.0 0
7.2600
5.3568
5.3460
3.5444
3.5307
3.5219
3.5131
3.4994
2.3071
2.2866
2.2808
2.2563
2.2300
2.1050
2.0796
1.9966
1.9624
1.8569
1.8511
1.8296
1.7681
1.7447
1.7251
1.6870
1.6743
1.6685
1.6558
1.6421
1.5835
1.5445
1.5113
1.4888
1.4712
1.4624
1.4507
1.4370
1.4224
1.3951
1.3882
1.3804
1.3687
1.3443
1.2896
1.2232
1.1968
1.1871
1.1558
1.1421
1.1304
1.1197
1.1080
1.0982
1.0796
1.0718
1.0513
1.0357
1.0108
0.9781
0.9537
0.9429
0.9312
0.9195
0.9088
0.8980
0.8824
0.8648
X : parts per Million : 1H

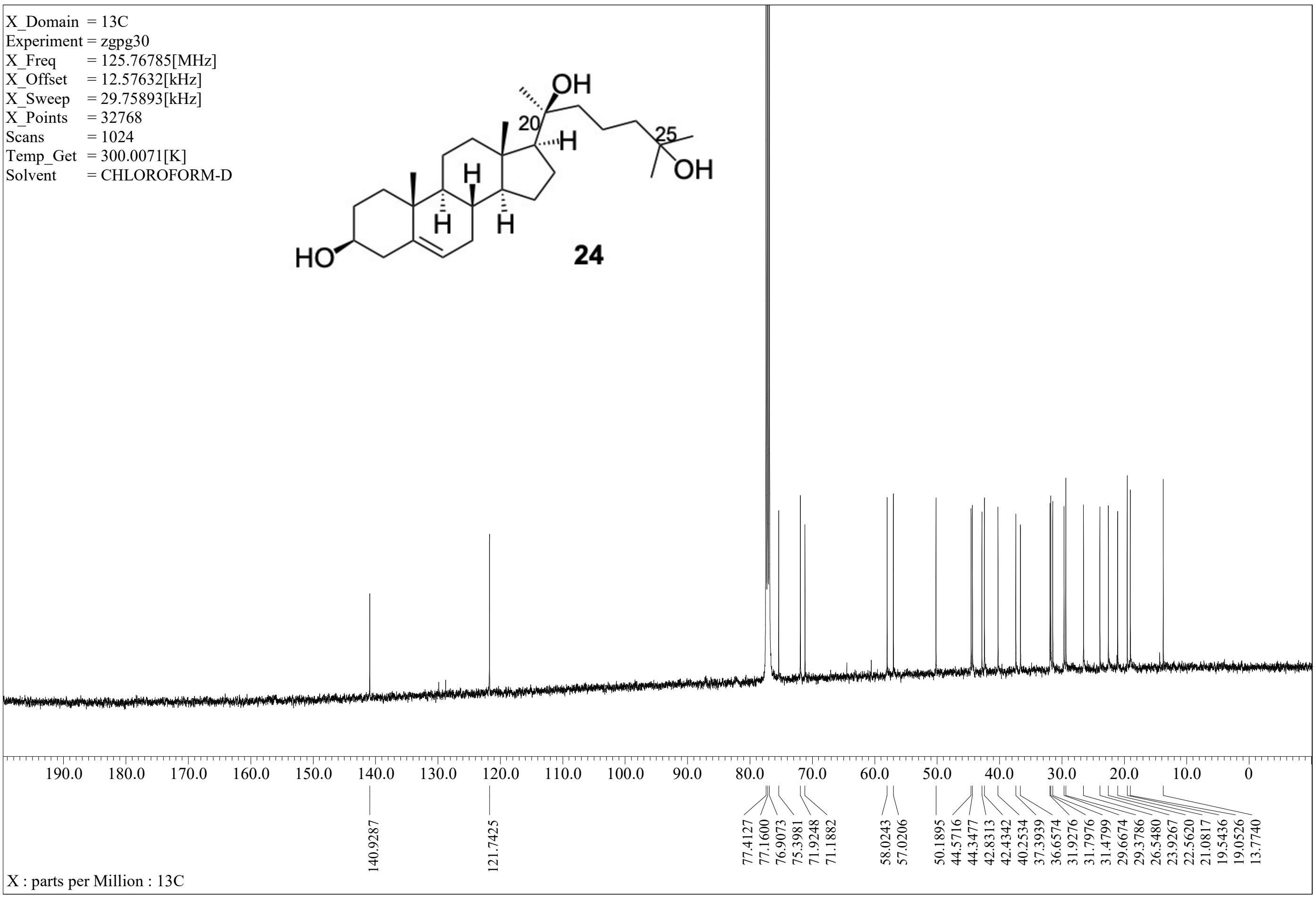
X_Domain = 13C
Experiment = zgpg30
X_Freq = 125.76785[MHz]
X_Offset = 12.57632[kHz]
X_Sweep = 29.75893[kHz]
X_Points = 32768
Scans = 1024
Temp_Get = 300.0071[K]
Solvent = CHLOROFORM-D
OH
20
H
25
OH
H
H
H
HO
24
190.0 180.0 170.0 160.0 150.0 140.0 130.0 120.0 110.0 100.0 90.0 80.0 70.0 60.0 50.0 40.0 30.0 20.0 10.0 0
140.9287
121.7425
77.4127
77.1600
76.9073
75.3981
71.9248
71.1882
58.0243
57.0206
50.1895
44.5716
44.3477
42.8313
42.4342
40.2534
37.3939
36.6574
31.9276
31.7976
31.4799
29.6674
29.3786
26.5480
23.9267
22.5620
21.0817
19.5436
19.0526
13.7740
X : parts per Million : 13C
